# The 29th Scientific Conference of the Society on NeuroImmune Pharmacology in Omaha, NE, June 8–12, 2025

**DOI:** 10.1515/nipt-2025-0004

**Published:** 2025-04-10

**Authors:** Allison M. Andrews, Linda Chang, Rosemarie Booze, Sabita Roy, Santhi Gorantla, IIker K. Sariyer, Jerel Fields, Eliseo Eugenin, Howard E. Gendelman

**Affiliations:** Department of Pathology, Immunology, and Laboratory Medicine, University of Florida, College of Medicine, Gainesville, FL, USA; Department of Diagnostic Radiology and Neurology, Nuclear Medicine, and Neurology, University of Maryland School of Medicine, Baltimore, MD, USA; Department of Psychology*,* University of South Carolina, Columbia, SC, USA; DeWitt Daughtry Family Department of Surgery, Miller School of Medicine, University of Miami, Miami, FL, USA; Department of Pharmacology and Experimental Neuroscience, 12284 University of Nebraska Medical Center, Omaha, NE, USA; Department of Microbiology, Immunology and Inflammation, Lewis Katz School of Medicine-Temple University, Philadelphia, PA, USA; Department of Psychiatry, University of California San Diego College of Medicine, Vc-health Sciences, La Jolla, CA, USA; Department of Neurobiology, The University of Texas Medical Branch (UTMB), Galveston, TX, USA

**Keywords:** neurodegenerative diseases, drug abuse, human immunodeficiency virus, viral pathogenesis, inflammation, neurobiology

## Abstract

The 29^th^ Scientific Conference of the Society on Neuroimmune Pharmacology (SNIP) in Omaha, NE, will occur from June 8^th^ to 11^th^, 2025. This four-day conference showcases world-renowned biomedical research, providing insights into the latest advancements in the intersecting fields of neuroscience, immunology, pharmacology, and virology. Presentation abstracts are organized into sections that include early career development investigators, mouse models, neurodegenerative diseases, therapeutics, substance use disorders, counseling, drug targeting, disease pathobiology, Blood-Brain Barrier integrity, educational opportunities, young investigator talks, and translational medicine. SNIP remains the sole global meeting dedicated to neuroimmune pharmacology. The focus of research centers on how the neuroimmune axis connects drug abuse, inflammation, and brain functional integrity. The conference features several plenary speakers who have made unique and significant contributions to their fields alongside renowned physician-scientists and luminaries. Symposia will include the SNIP Presidential Symposium on Pathobiology and Novel Therapies for Neurodegenerative Diseases, Ultra Long-Acting Medicines, Development and Delivery of Diagnostic and Therapeutic Biomarkers to disease regions, overcoming barriers to treating neurological disorders, neuroinflammation, and reward pathways for addiction, as well as neuron-glia interaction. All presentations are framed within the context of microbial infections, drugs of abuse, and therapeutics. Therapeutics include nanopharmacology and advances in informatics analysis of multi-omics data to decipher the complex cell and molecular interactions that underpin the function of the nervous system. SNIP member symposia and a local series of presentations will highlight outstanding talent from the University of Nebraska. Additional events include lunch with NIH program officials and a NeuroImmune Pharmacology and Therapeutics Journal dinner. The goal is to unite investigators from diverse basic, clinical, and translational fields to discuss and advance our understanding of the multifactorial impact of substance abuse, inflammation, and infections critical to human health. We aim to engage and mentor young investigators in neuroimmune pharmacology and disseminate information presented at the conference to the scientific community, the general public, and healthcare providers. Cultivating the next generation of scientists is vital to our mission. The agenda encompasses early-career investigator presentations, poster sessions, meet-the-mentors luncheons, and a special panel of junior faculty. The conference also provides an enriching environment for scientists and clinicians to share ideas, foster the next generation of scientists, and promote current disease pathobiology and therapeutics trends. Opportunities to visit the Omaha zoo will be available with guest passes. We thank Dr. Carol Swarts, the Robert Eisenberg Family, Howard Kooper, the Gendelman Family Research Endowment, Fisher Scientific, Amy Sather, and the research community for sponsoring this meeting and its exchanges.

The 29^th^ annual meeting of the SNIP will take place from June 8^th^ to 11^th^, 2025, at the Embassy Suites in downtown Omaha, Nebraska. This location is central to Omaha’s culture and provides easy transportation to and from the airport. The hotel’s conference area is fully equipped, ensuring easy movement and high accessibility for attendees. The added advantage of having the hotel and conference in the same building means attendees will have fewer concerns regarding logistics and travel. The SNIP conference leadership has been a longtime and dedicated supporter of the NIH mission to ‘support innovative training, career development, and education for the biomedical research workforce’. As a society with a diverse composition, we understand the tremendous challenges in training the next generation of scientists in our field. We acknowledge the overwhelming evidence strongly indicating that teams of individuals with diverse thinking styles are more effective in tackling complex problems, resulting in enhanced problem-solving abilities, heightened invention, and applicable solutions. Our society boasts a membership composed of individuals from all continents.

As an American scientific society, we are proud of our continued commitment to offer a place where young scientists can access mentorship, networking, and educational opportunities. An organizing committee was formed to enhance conference recruitment, participation, and trainee mentorship. For nearly 10 years, SNIP has been an instrument for developing new networking gatherings for mentoring, podium presentations highlighting young investigators’ research and educational workshops to help up-and-coming investigators find mentors and navigate scientific opportunities in and beyond academia. Predoctoral and postdoctoral travel awards are given for travel support. These awards include registration waivers, allocation of funds for travel to and from the conference, and hotel fees. Promising young scientists accepted for travel awards are also selected to present their research in the featured poster session. Up to 6 pre-doctoral and postdoctoral researchers are selected for oral presentations. We will host a mixer and small group discussions that bring together trainees with our society’s most successful and established scientists. The intention is for trainees to secure possible postdoctoral training opportunities, learn of biomedical science career paths, get career advice, or nucleate scientific collaborations. For the SNIP conference 2025, we have organized junior faculty panels to speak regarding their academic career and the challenges they’ve overcome.

SNIP scientific meetings are focused on uncovering how substance abuse renders humans more susceptible to infection and chronic diseases, with a particular focus on human immunodeficiency virus-1 (HIV-1). It is the only conference in the world devoted to neuroimmune pharmacology and the neuroimmune axis as it relates to drug abuse, inflammation, and brain infections (primarily neuroHIV). Our 2025 program features several seminal presentations, never before included in the Society’s agenda. These include plenary speakers, each with unique and significant contributions to their fields. The keynote is by distinguished Professor Robert Gallo, who co-discovered HIV as the cause of AIDS, revealed nearly all of the virus’ access gene functions, discovered interleukin-2, HTLV-I, II, and the herpes virus link to Kaposi’s sarcoma. Dr. Tsuneya Ikezu, considered a luminary in studies of neurodegenerative disease pathobiology, will give our memorial lecture. These speakers represent a diverse range of expertise showcasing the breadth of knowledge at the conference. Other highlights are Drs.

Jim Rooney (Vice President of Gilead), Charles Flexner (Professor of Medicine and Pharmacology at Johns Hopkins Medical Center), Paul Domanico (Senior Director, Clinton Health Access Initiative), and Christopher J. Wheeler (co-founder and Chief Science Officer at T-Neuro Pharma, President of StemVax Therapeutics and Senior Research Scientist at the World Brain Mapping Foundation).

Symposiums include the SNIP Presidential Symposium on “Pathobiology and Novel Therapies for Neurodegenerative Diseases,” “Ultra Long-Acting Antiretroviral Drugs,” “Development and Delivery of Diagnostic and Therapeutic Biomarkers to the Brain,” “Assessing the Blood-Brain Barrier in Neurological Disorders and Inflammation,” “Neuroinflammation in the Reward Pathway of Addiction,” “Neuron-glia Interaction in the Context of HIV and Drugs of Abuse,” “Nano-pharmacology: Nanotechnology-Based Therapeutics for Infection and Inflammation,” and “Advances in the Informatics Analysis of Multi-Omics Data to Disentangle the Complex Interactions of HIV Infection with Drugs of Abuse”. We also include a SNIP member symposium selected from the submitted abstracts and a Local Organizing Committee Symposium featuring outstanding talent from the University of Nebraska Medical Center. Additional highlights include lunch with NIH program officials and the NeuroImmune Pharmacology and Therapeutics (NIPT) Journal Dinner. SNIP has attracted and cultivated a diverse membership and attendance from national and international experts. A key bedrock of the program is through its development of young investigators. In 2024, 38 travel awards were awarded to outstanding scientists, and oral presentations were given by the top awardees. Overall, the conference provides an enriching environment for scientists and clinicians to share ideas, foster the next generation of scientists, and learn about the new trends in the field. Evidence of our sustained impact is reflected by our executive board, which comprises one-third of members who began as pre/post-doctoral trainees in SNIP and are now assistant or associate professors.

**Dissemination of Conference Results:** All speakers and poster presenters will be solicited to submit their scientific work to the society’s journal. The journal offers rapid, high-quality reviews of manuscripts as well as open-access publication. Significantly, NIPT has allowed free journal publication through 2025. This will encourage the fast dissemination of research findings presented at the conference. The editor-in-chief, Dr. Howard E. Gendelman, will promote the new submissions through face-to-face engagements, emails, and personal solicitations through special issues. In addition to the solicited individual articles, the journal will publish all SNIP conference abstracts as a standard or supplementary issue of NIPT. SNIP has a longstanding track record in the dissemination of the abstracts for the conference. Before 2022, abstracts for clinical and translational science were published elsewhere. Innovation is at the center of the SNIP conference, which fosters an uncompromising dedication to support the next generation of scientists without leaving behind our talented trainees, and a journal dedicated to the scientific outcomes of the conference. In addition to the publication in NIPT, the conference proceedings and the link to the abstract publications are posted on the SNIP website. After each conference, a survey is conducted to assess the outcomes of the meeting. Feedback from participants includes evaluation of the poster sessions, conference format, presentations, facilities, site location, suggestions for future symposium sessions, educational programs, conference venues, and potential speakers.

**Organization of Subsequent Conferences:** Planning for the 30^th^ SNIP Scientific meeting is underway. The focus of the SNIP annual scientific meeting remains on the intersection between neuropharmacology, neuroimmunology, and brain infections (primarily HIV-1 infection). The SNIP meetings committee also considers emerging trends and topics while planning the annual meeting. The President-Elect, Dr. Linda Chang, MD, will preside as chair for the 30^th^ SNIP Scientific meeting. This meeting will be held in Baltimore, MD. Dr. Chang is particularly excited to highlight the outstanding local researchers both from clinical and basic science. Potential topics include “Substance use during pregnancy,” “Brain development and aging,” and “HIV and advanced aging.” The Meetings Committee will decide the final meeting symposia by summer 2025.

**Table:** 

**Schedule for the 29**^**th**^ **Annual Society of Neuroimmune Pharmacology Conference**	
**Sunday, June 8th**	
12:00 – 5:30 PM	Registration Open (Registration desk by side entrance doors)
1:00 – 3:00 PM	**Pre-conference workshop 1:** Creation, Care, and Translation of Humanized Mouse for HIV/AIDS Research **Moderator:** Santhi Gorantla, PhD, and Howard E. Gendelman, MD, University of Nebraska Medical Center Angela Wahl, PhD, Associate Professor, Microbiology, University of Alabama at Birmingham **Next-Generation Humanized Mouse Models of HIV/AIDS Research** Jennifer Koblinski, PhD, Associate Professor, Virginia Commonwealth **Advancing Research with Humanized Mice: Unlocking the Potential of Shared Resources** Santhi Gorantla, PhD, Professor, Pharm. and Exp. Neuroscience, University of Nebraska Medical Center **Novel Humanized Mouse Models for NeuroHIV** Ramesh Akkina, PhD, Professor, Microbiology, Immunology, and Pathology, Colorado State University **Understanding HIV Evolution, Latency, and Elite Control Using Humanized Mice** **Summary Discussion:** Paul Denton, PhD, University of Nebraska-Omaha
3:00 – 3:15 PM	**Break**
3:15 – 5:30 PM	**Pre-conference workshop 2:** Mentoring and Opportunities for Early-Stage Investigators in Neuroscience (ESINs) **Moderator:** Siddappa Byrareddy, PhD. Professor and Vice-Chair of Research, University of Nebraska Medical Center, **Co-chairs:** Vasudev Rao, MD, NIH/NIMH; William Daley, PhD, NIH/NINDS and Kathleen Borgmann, PhD, NIH/NIDA **Opening remarks:** NIMH/NINDS/NIDA officials **Overview and Networking opportunities:** Vinayaka R. Prasad, PhD, Professor, Associate Chair of Academic Affairs, Core Director, ERC CFAR Developmental Core, Program Director, *Training in HIV/AIDS Pathogenesis* T32 Program, Albert Einstein College of Medicine, Bronx, NY

**Table:** 

	**Mentoring and Engagements:** Amanda Brown, PhD, Director, Johns Hopkins NeuroHIV-Comorbidities Scholars Program; Director, Johns Hopkins Internship in Brain Sciences Program **Round Table Discussion:** Yisel M. Cantres-Rosario, PhD, University of Puerto Rico, Aditya Bade, PhD, University of Nebraska Medical Center, Kathleen Borgmann, PhD, NIH/NIDA, Siddappa Byrareddy, PhD, University of Nebraska Medical Center
5:15 – 6:00 PM	**Break**
6:00 – 8:00 PM	**ECITA Poster Session and Meet and Greet**
**Monday, June 9th**	
8:00 – 9:30 AM	**Symposium 1: Welcome from the President: Presidential Symposium** **Pathobiology and Novel Therapies for Neurodegenerative Diseases** **Chair/Co-Chair:** Howard E. Gendelman, MD, and R. Lee Mosley, PhD, University of Nebraska Medical Center Howard E. Gendelman, MD, Professor, UNMC, Omaha, NE **Next generation cellular therapies for neurodegenerative disorders** Serge Prezdborski, PhD, Distinguished Professor of Neurology, Columbia University, New York **Neuropathogenesis of Degenerative Diseases of the Nervous System** Christopher Wheeler, PhD, Director T-Neuro Pharma, Santa Cruz, CA **The promise of targeting cytolytic T cells in Alzheimer’s treatment and prevention** Susmita Sil, PhD, Assistant Professor, UNMC, Omaha, NE **Monocyte Biomarkers for Early Stage Sargramostim Parkinson’s Disease Therapy**
9:30 – 10:00 AM	**Toby Eisenstein Memorial Lecture** Mary Lou Falcone, producer, visionary, and author of, *I Didn’t See it Coming:Scenes of Love, Loss and Lewy Body Dementia* **I Didn’t See it Coming: Personal Insights for Lewy Body Dementia**
10:00 – 10:15 AM	**Break**
10:15 – 11:45 AM	**Symposium 2: Ultra-long-acting antiretroviral drugs** **Chairs:** Brady Silman, PhD, Assistant Professor, University of Nebraska Medical Center and Sudipta Panja, PhD, Assistant Professor, University of Nebraska Medical Center Charles Flexner, MD, Professor, Johns Hopkins University Medical Center, Baltimore, MD **How Long-Acting Formulations Are Revolutionizing the Treatment and Prevention of HIV** Benson Edagwa, PhD, Professor, University of Nebraska Medical Center, Omaha, NE **Ultra-Long-Acting Slow Effective Release Therapies** Jim Rooney, MD, Senior Vice President, Gilead, Foster City, CA **Lenacapavir for the Treatment and Prevention of HIV infection** Paul L. Domanico, PhD, Director, Clinton Health Access Initiative, Raleigh, NC **The Global Health Sciences Department at Clinton Health Access**
11:45 – 12:15 PM	**Drs Mahendra and Adarsh Kumar Memorial Lecture** Dr. Tsuneya Ikezu, MD, PhD, Professor Mayo Clinic, Jacksonville, FL **Cargo lipids in APOE4 brain extracellular vesicles facilitate Tau propagation in Alzheimer’s disease**
12:30 – 1:30 PM	**Lunch Symposium 3 Sponsored by Fisher Scientific** **Chairs:** Jerel A. Fields, PhD, Associate Professor University of California at San Diego and Shamshudeen Moidunny, Assistant Professor, University of Miami 1. **Alzheimer’s and neuroHIV: A comparative analysis of metabolic pathway in postmortem brain tissues**. Ali Boustani, MD, University of California, San Diego School of Medicine 2. **Exploring the effects of chronic oral inoculation of actinomyces meyeri on behavioral and neurobiological changes in b6 mice**. Tabinda Salman, PhD, Medical University of South Carolina 3. **Quantitative proteomics of astrocyte-derived exosomes reveal potential novel biomarkers of HIV-associated neurocognitive impairment**. Lester J. Rosario Rodriguez, PhD, University of Puerto Rico, Medical Sciences Campus 4. **Structural brain abnormalities and neuropsychiatric symptoms in post-covid condition**. Meghann Ryan, MS, University of Maryland 5. **The effect of estrogen and fentanyl on HIV replication and immune migration across the Blood-Brain Barrier**. Maya Basic, MS, University of Florida 6. **Cocaine impairs anti-viral response in ipsc-microglia to accelerate HIV infection via sigma-1**. Tofunmi Oteju, BS, Drexel University College of Medicine 7. **Fentanyl enhances HIV infection of macrophages and microglia**. Qianhao Xiao, PhD, Temple University 8. **Optimizing HIV neurotherapy with curcumin adjuvant**. Sandip P. Godse, MS University of Tennessee Health Science Center 9. **Sri-47056 a pyrimidine structure-based compound allosterically modulates monoaminergic transporters, attenuating the dysregulation of dopamine and serotonin uptake induced by HIV-1 transactivator of transcription (TAT)** Ana Jimenez Torres, PhD, Department of Drug Discovery, Collage of Pharmacy University of South Carolina

**Table:** 

	10. **Myeloid targeted disulfide lipid nanoparticles with enhanced endosomal escape improves HIV-1 excision**. Soumya Sagar Dey, MS, University of Nebraska Medical Center 11. **Incomplete glial recovery despite ART: Insights from scrnaseq in a triple humanized glial mouse model of HIV brain infection**. Suresh Kondeti, PhD, University of Nebraska Medical Center 12. **Effects of methamphetamine (meth) on HIV integration and latency in a cellular microglia model**. Wei Ling Lim, PhD, San Diego Biomedical Research Institute
1:30 – 2:00 PM	**Dual Event NIH workshop with Program Officers; Grant Writing for Fellowships and Career Awards**
2:00 – 2:30 PM	**Plenary Talk** Jonathan Rockman, Behavior Health and Social Service Providers, Addiction disorders, Omaha, NE **Counseling the opioid crisis and withdrawal syndrome**
2:30 – 4:00 PM	**Symposium 4: Development and Delivery of Diagnostic and Therapeutic Biomarkers to the Brain** **Chairs:** Linda Chang, MD, Professor, University of Maryland School of Medicine and Piotr Walczak, MD, PhD, Professor, University of Maryland School of Medicine Piotr Walczak, MD, PhD, Professor, Dept of Diagnostic Radiology & Nuclear Medicine, University of Maryland School of Medicine **Multi-Modality Imaging for Precision Delivery of Cells and Biologics to the Brain** Assaf Gilad, PhD, Professor of Chemical Engineering, Material Sciences and Radiology, Michigan State. **From seaside to bedside: bioengineering proteins for diagnostics and treatment** Chengyan Chu, MD, Research Associate, Diagnostic Radiology-Nuclear Medicine, University of Maryland **Intra-arterial route with osmotic Blood-Brain Barrier opening for enhanced drug delivery to the brain** Wojciech Lesniak, Ph.D., Assistant Professor, Department of Radiology, UT Southwestern Medical Center **Evaluation of brain injuries and neuroinflammation with PET imaging**
4:00 – 4:15 PM	**Break**
4:15 – 5:45 PM	**Symposium 5: Assessing the Blood Brain Barrier in Neurological Disorders and Inflammation** **Chairs**: Maria Cecilia Marcondes, PhD, San Diego Biomedical Research Institute, and Yuri Persidsky, MD, PhD, Chair and Professor, Temple University Jennifer Ludicello, PhD, Assistant Professor University of California San Diego San Diego CA **The signatures of Blood-Brain Barrier disruption in HIV and the effects of Cannabis Use Patterns** Silvia Torices, PhD, Scientist, Miami Miller School of Medicine, Miami, FL **Stroke Vulnerability in the Context of HIV Infection** Richard Milner, MD, PhD, Senior Professor, San Diego Biomedical Research Institute, San Diego, CA **The protective role of microglia to the Blood-Brain Barrier in Aging** Violaine Delorme-Walker, PhD, Scientist, San Diego Biomedical Research Institute, San Diego, CA **Effects of antiretrovirals on the functional integrity of the Blood-Brain Barrier** Naveen Mekala, PhD, Post-Doctoral Fellow, Temple University, Philadelphia, PA **Alcohol and e-cigarette exposure induce release of extracellular vesicles and their plasminogen urokinase content promoting Blood-Brain Barrier injury**
6:00 – 7:30 PM	**General abstracts POSTER Session and Neuroimmune Pharmacology and Therapeutics Dinner**
**Tuesday, June 10** ^**th**^	
8:00 – 9:30 AM	**Symposium 6: Neuroinflammation in the reward pathway of addiction** **Chairs:** Allison M. Andrews PhD, Associate Professor, University of Florida, Gainesville, FL and Jay McLaughlin PhD, Professor, University of Florida, Gainesville, FL Servio H. Ramirez, PhD, Professor Department of Pathology, Immunology & Laboratory Medicine at the University of Florida College of Medicine, Gainesville, FL **Neuroinflammatory signatures in the reward pathway following brain injury suggest vulnerability to an enhanced addiction phenotype** Cassie Gipson-Reichardt, PhD, Associate Professor University of Kentucky College of Medicine **Chronic nicotine use suppresses accumbens immune signaling** Kristen McLaurin, PhD, Assistant Professor, University of Kentucky College of Pharmacy, Lexington, KY **HIV-1 and/or opioid use disorder induce functional alterations in microglia** Stephanie Daws, PhD, Assistant Professor, Temple University Lewis Katz School of Medicine, Philadelphia, PA **Targeting neuroinflammatory signaling in the brain for the reduction of opioid-induced phenotypes**
9:30 – 10:15	**Symposium 7: Translational Investigators** **Chair:** Marcus Kaul, PhD, Professor of Biomedical Sciences, University of California Riverside, CA Yisel Cantres, PhD, Assistant Professor, University of Puerto Rico, PR Myosotys Rodriguez Martinez, PhD, Assistant Professor, Immunology-Nanomedicine, Florida International, Miami, FL Kimberly Williams PhD, Assistant Professor Environmental and Health Sciences Program, Spelman College, Atlanta, GA Pravin Yeapuri PhD, Instructor, University of Nebraska Medical Center

**Table:** 

10:15 – 10:30	**Break**
10:30 – 12:00	**Symposium 8: Neuron-glia interaction in the context of HIV and/or drugs of use/misuse** **Chairs**: Shao-Jun Tang, PhD, Stony Brook Medicine Endowed Professorship in Anesthesiology Vice Chair for Research, Renaissance School of Medicine at Stony Brook University, Stony Brook, NY, and Pamela Knapp, PhD, Professor, School of Medicine, Anatomy and Neurobiology, Virginia Commonwealth University Sheetal Sreeram, MS, Graduate Student, Case Western Reserve University School of Medicine, Cleveland, OH **HIV-1 Infection of Brain Organoids and METH Exposure Dysregulate Homeostatic Microglia-Neuron-Astrocyte Interactions by Unique Pathways** Viktor Yarotskyy, PhD, Assistant Professor, Virginia Commonwealth University, Richmond, VA **Multiple receptor-mediated effects of fentanyl and xylazine on neuronal activity of striatal medium spiny neurons** Ming-Lei Guo, PhD, Associate Professor, Eastern Virginia Medical School, Norfolk, VA **HIV-TAT dysregulates microglial lipid metabolism through SREBPr-124 axis: implication of lipid droplet accumulation microglia in neuroHIV** Shao-Jun Tang, PhD, Professor, Renaissance School of Medicine at Stony Brook University, Stony Brook, NY **Interactions between neurons and astrocytes during the pathogenesis of HIV-associated pain**
12:00 PM	**Lunch on your own or Meet the Mentor Lunch**
1:00 – 2:30 PM	**Symposium 9: Nanopharmacology: Nanotechnology-based therapeutics for Infection and Inflammation** **Chairs**: Supriya D. Mahajan PhD MPH**,** Associate Professor and Jessica L. Reynolds PhD Associate Professor Jacobs School of Medicine University at Buffalo, Buffalo NY Supriya D. Mahajan PhD**,** Associate Professor, Department of Medicine, Jacobs School of Medicine & Biomedical Sciences, University at Buffalo, Buffalo, NY **Nanotechnology approach to target Neuroinflammation** Alexander Khmaladze, PhD, Associate Professor, Department of Physics, SUNY Albany, Albany, NY **3D Nano-Imaging at the Sub-Cellular, Cellular and Multicellular Levels** Hilliard Kutscher, PhD, Senior Scientist, POP Biotechnologies, Buffalo, NY **Laser ablation – a unique methodology to nanoformulate HIV antivirals** Anna V. Sharikova PhD Assistant Professor in the Physics department at University at Albany **Raman hyperspectral imaging and analysis of complex biological systems**
2:30 PM	**Omaha Zoo Visit and Carpool or Free afternoon**
6:30 – 8:30 pm	**Banquet** **Speaker:** Robert Gallo, MD Distinguished Professor University of South Florida and Chair, Scientific Leadership Board of the GVN **Viral Immunology Past, Present and Future**
**Wednesday, June 11** ^**th**^	
8:00 – 9:30 AM	**Symposia 10: Advances in the Informatics analyses of Multi-Omics Data to Disentangle the Complex Interactions of HIV Infection with Drugs of use/misuse** **Chairs:** Jay Rappaport, PhD, Director of the Tulane National Primate Research Center, New Orleans, LA, and Norman J. Haughey, PhD., Professor, Tulane University School of Medicine, New Orleans, LA Uma Maheswari Deshetty, PhD, Post-Doc University of Nebraska Medical Center, Omaha, NE **Morphine potentiates HIV Tat-mediated neuroinflammation and neurodegeneration** Sabita Roy, PhD, Professor and Vice Chair Department of Surgery, University of Miami, Miami, FL **Single-cell transcriptomics reveals neuron- and glia-specific transcriptional changes in the adolescent midbrain following neonatal morphine exposure, which are ameliorated with probiotic intervention** Qingsheng Li, PhD., Willa Cather Professor Biological Sciences, University of Nebraska Lincoln **Exploring the biology of HIV-1 latency in the CSN as a pathway to developing a cure** Dionna Williams, PhD, Associate Professor, Emory University, Atlanta, GA **Cannabidiol Decreases HIV/SIV Infection, Reduces the Latent Viral Reservoir, and Suppresses Inflammatory Cytokines in Rhesus Macaques and in vitro studies with Primary Human Cells**
**10:00 – 10:30 AM**	**Bill Narayan Memorial Lecture** Howard Fox, PhD, Professor, University of Nebraska Medical Center, Omaha, NE **Omics unleashed: revolutionizing neuroimmune pharmacology with data-driven science**
**10:30** – **10:45 AM**	**Break**
**10:45 – 12:00 Noon**	**Symposium 11: SNIP member symposium** **Chair:** Richard J. Noel, PhD., Professor of Biochemistry, Department of Basic Sciences, Ponce Health Sciences University Selected talks from general abstracts
**12:00** – **1:00 PM**	**SNIP Business Meeting for members** Lunch on your own

**Table:** 

**1:30** – **3:00 pm**	**Symposium 12: Local organizing committee symposium** **Chair:** Prasanta Dash, PhD, Associate Professor, Pharmacology and Experimental Neuroscience, University of Nebraska Medical Center, Omaha, NE Prasanta K. Dash, PhD., Assistant Professor, University of Nebraska Medical Center **Accelerated Dysregulation of Neuroimmune Markers in HIV-infected and Aged Humanized Mice** Han-Jun Wang, MD., Endowed Professor in the Department of Anesthesiology, UNMC **Neural Mechanisms underlying Cardiac Dysfunction and Cardiac Remodeling in Chronic Heart Failure** Sowmya Yelamanchili, PhD., Associate Professor, Department of Anesthesiology, UNMC **Pathogenesis and therapeutic insights into degenerative and substance use disorders** Chen Zhang, PhD., Post-doctoral fellow, Pharmacology and Experimental Neuroscience, UNMC **Molecular Signatures in Rebound Viruses from Antiretroviral Drug and CRISPR Treated HIV-1-Infected Humanized Mice** Arpan Acharya, PhD., Assistant Professor, Pharmacology and Experimental Neuroscience, UNMC **Aging and Reservoirs in Persons Living with HIV (PLWH)**
3:00 PM	**End of Conference**

## Pre-Conference Workshops

**1. Creation, Care, and Translation of Humanized Mouse for Research**. This workshop brings together humanized mouse model experts to discuss their development, care, and applications in HIV/AIDS research. Humanized mice, engineered with functional human cells and tissues, are commonly used pre-clinical models for studying human diseases, immune responses, and testing therapies. These models are used most notably in infectious diseases, cancer, and immune disorders. All will be discussed through interactive exchanges with experts.

The key presentations

– “Next-Generation Humanized Mouse Models of HIV/AIDS Research”
  Speaker: Angela Wahl, PhD (University of Alabama at Birmingham)
– “Advancing Research with Humanized Mice: Unlocking the Potential of Shared Resources”
  Speaker: Jennifer Koblinski, PhD (Virginia Commonwealth University)
– “Novel Humanized Mouse Models for NeuroHIV”
  Speaker: Santhi Gorantla, PhD (University of Nebraska Medical Center)
– “Understanding HIV Evolution, Latency, and Elite Control Using Humanized Mice”
  Speaker: Ramesh Akkina, PhD (Colorado State University)

**2. Mentoring and Opportunities for Early-Stage Investigators in Neuroscience**

This symposium is intended for early-stage investigators (ESIs) focusing on neuroscience-related research, including neurodegenerative diseases. The participating ESIs—students, postdocs, and junior faculty—will have opportunities to enhance their career excellence. The event will feature NIH staff, senior faculty, and ESIs who will provide insights on career development and offer a venue for forming new collaborations.

The key highlights and objectives of this workshop.

– Support early-stage investigators in neuroscience with mentorship, training, and funding opportunities.
– Enhance research capacity by fostering collaborations between junior researchers and senior mentors in neuroscience.
– Provide structured career development resources, including grant writing, networking, and leadership skills.
– Facilitate interdisciplinary collaborations, particularly in neurovirology, gut-brain connections, neuroimmunology, and therapeutics/animal models.

## Symposium 1: Welcome from the President: Presidential Symposium: Pathobiology and Novel Therapies for Neurodegenerative Diseases

### Next-cellular therapies for neurodegenerative disorders

Gendelman, H.E, MD, Department of Pharmacology and Experimental Neuroscience, University of Nebraska Medical Center, Omaha, NE

Based on strategies for cancer treatment, chimeric antigen receptor (CAR) T-cell platforms are being developed for treating neurodegenerative diseases. CAR T-cells utilize antigen-recognition regions from the variable domains of antibodies. A cDNA encoding the variable regions of antibodies generated in our laboratories was constructed to encode a single-chain peptide of the variable fragments to retain antigen reactivity. This expression results in the transmembrane orientation of external antigen recognition. Ligation of cognate antigens can initiate CARs that target misfolded proteins associated with their respective disorders. These disorders include amyloid β (Aβ) for Alzheimer’s disease, superoxide dismutase 1 (SOD1) or Tar DNA binding protein-43 (TDP-43) for amyotrophic lateral sclerosis, and modified α-syn, such as nitrated or phosphorylated α-syn, for Parkinson’s disease. This provided an appropriate recognition target. Strategies for this platform include Treg isolation, deletion of an endogenous T-cell receptor, transduction of the CAR construct, and cell expansion developed in our laboratories, which will be discussed during the president’s symposia.

### Present and future insights into the pathobiology of neurodegenerative diseases

Przedborski, S. MD, PhD Professor and Co-Director, Motor Neuron Center; Vice Chair of Research, Department of Neurology; Director, Columbia Translational Neuroscience Columbia University

Dr. Przedborski’s academic interests focus on the molecular and cellular biology of Parkinson’s disease and Amyotrophic Lateral Sclerosis. His ongoing research studies cell-autonomous and non-cell-autonomous neurodegeneration mechanisms using toxic and genetic experimental models. A focus area of laboratory research is how alterations in mitochondrial dynamics and mitophagy provoke the degeneration of neuronal subpopulations. A second main line of research is to what extent and by which mechanisms microglia and astrocytes participate in the pathobiology of neurodegeneration.

### The Promise of targeting cytolytic T cells in Alzheimer’s treatment and prevention

Wheeler, C.J., PhD, Research & Development, T-Neuro Pharma, Aptos, CA, 95001

We recently developed a CD8 T cell-based mouse model that recapitulates definitive hallmarks of Alzheimer’s disease (AD), including amyloid plaque and neurofibrillary tangle deposition, robust neurodegeneration, and profound cognitive decline following an AD-like pattern of progression. Functional impairment of the T cells in this model arrested AD-like pathology, and blood levels of analogous antigen-specific CD8 T cells were explicitly associated with human AD (DOI: 10.1073/pnas.2401420121). Because our findings established unique necessity and sufficiency of self-reactive CD8 T cells in AD-like pathophysiology, and further predicted previously unknown properties of the clinical disease, they support targeting CD8 T cells to improve diagnosis, treatment, and possibly prevention of sporadic human AD. In direct support of blood levels of analogues of the T cells that initiated AD pathophysiology in mice tracked AD and pre-AD status in human patients with >97% specificity in receiver operating characteristic (ROC) analysis. More indirectly, drugs that inhibit T cells have recently been reported to significantly reduce dementia risk in patients, highlighting the potential of targeting T cells in treating and/or preventing dementia including AD. Multiple clinical trials targeting T cells are now underway, which should help test the hypothesis that these cells are critically involved in AD initiation and progression. These studies could also help clarify AD’s biological and mechanistic basis, further promoting its effective clinical management.

Supported by T-Neuro Pharma (current); NIH/NIA (past); Joseph Drown Foundation (past); Maxine Dunitz Neurosurgical Institute (past)

### Monocyte Biomarkers for Early Stage Sargramostim Parkinson’s Disease Therapy

Sil, SS, PhD^1^, Akhter, SA, MS^1^, Kumar, MK, BS^1^, Du, XD, MS^1^, Saha, AS, BS^1^, Oludipe, DO, BS^1^, Shetty, TS, BS^1^, Santamaria, PS, MD^2^, Mosley, RLM, PhD^1^, Gendelman, HG, MD^1^; ^1^Pharmacology & Experimental Neuroscience, University of Nebraska Medical Center, Omaha, NE. ^2^Neurology Consultants of Nebraska, PC and Nebraska Medicine, Omaha, NE

Dysregulation of innate and adaptive immunity signifies both the development and progression of Parkinson’s disease (PD). Deficits in innate immunity in PD are marked by impairments in monocyte activation, function, and pro-inflammatory factors, which affect PD pathobiology. We have reported the clinical utility and safety of granulocyte-monocyte colony-stimulating factor, Sargramostim 1a and 1 b, in clinical trials for PD over a short period. Subsequently, Phase 1c was conducted to assess the role of a lower dose of Sargramostim regarding safety and tolerability and to evaluate monocyte biomarkers associated with immune transformative therapy for PD over 12 months. Eight of ten patients at the 12-month mark exhibited a significant reduction in UPDRS III scores during Sargramostim therapy without any recorded adverse events. PD patients were divided into three subgroups based on treatment-related changes in their UPDRS III scores, categorized as potent, modest, and stable, with score reductions of 7–13, 3–7, and 0–3, respectively. Significant improvements in response to Sargramostim therapy were correlated with autophagy and anti-inflammatory and anti-oxidant markers such as ATG7, HMOX1, RELA, and TLR8. The modest group was associated with RELA and LRRK2, while the stable group was linked to GABARAPL2. RNA sequencing and transcriptomic analysis identified additional anti-inflammatory, anti-oxidant, calcium-binding proteins, and epigenetic regulators that correlated with improved motor functions. These monocyte-based biomarkers will facilitate the tracking of clinical drug-related improvements and will be validated in an upcoming Phase 2 clinical trial. Partner Therapeutics, Inc. supported this project.

### Toby Eisenstein Memorial Lecture

Mary Lou Falcone is the author of *I DIDN’T SEE IT COMING: Scenes of Love, Loss, and Lewy Body Dementia*.

#### I Didn’t See it Coming: Personal Insights from Lewy Body Dementia

Ms. Falcone is internationally known as a classical music publicist/strategist who for 50 years has helped guide the careers of celebrated artists – Van Cliburn, Gustavo Dudamel, Renée Fleming, Sir Georg Solti, James Taylor – and advised many institutions including Carnegie Hall, Chicago Symphony, Los Angeles Philharmonic, Philadelphia Orchestra, New York Philharmonic, Vienna Philharmonic. Combining communication skills with her background as a performer and educator, she now adds another layer: advocate for Lewy Body Dementia (LBD) awareness. Her late husband, the illustrator/painter Nicky Zann, who died from LBD in 2020, catalyzed her book. She is also an Executive Producer of a new documentary film about LBD entitled, *Facing the Wind*.

## Symposium 2: Ultra long-acting antiretroviral drugs

### How Long-Acting Formulations Are Revolutionizing the Treatment and Prevention of HIV

Flexner, C., MD, Johns Hopkins University

Despite having near-perfect single tablet regimens, adherence to daily oral HIV treatment and prevention is unacceptably low in many settings. Long-acting and extended-release drugs and formulations hold promise for solving this problem and improving outcomes, facilitating the achievement of targets for controlling this epidemic. The first three LA/ER formulations for HIV treatment and prevention are now approved and available, but are underutilized in low- and middle-income countries, mainly because of access issues. There is a need for products with less frequent dosing, greater patient convenience, reduced risk of virologic failure, and regimens that suppress hepatitis B virus infection. Novel products should be accessible in resource-limited settings and for vulnerable populations, including children, adolescents, and pregnant women. Long-acting drug delivery also has the potential to transform the treatment and prevention of other infections including tuberculosis, malaria, and viral hepatitis. This presentation will provide an overview of the current state of development of LA formulations for HIV, and review recent advances in formulation science that will help make better formulations and platforms for drug delivery.

### Ultra-Long-Acting Slow Effective Release Therapies

Edagwa, B. PhD, Professor, ^1^Department of Pharmacology and Experimental Neuroscience, University of Nebraska Medical Center, Omaha, NE.

To ensure efficacy, existing antiretroviral therapy (ART) for prevention and treatment of HIV-1 and hepatitis B (HBV) infections requires life-long adherence to medicines. Some treatments’ limitations include shorter drug half-lives, variable pharmacokinetics profiles, and inadequate access to cellular and tissue reservoirs of infection. Pill fatigue and adverse drug reactions can negatively impact adherence. We have demonstrated that improved drug half-lives, tissue biodistribution and maintenance of optimal therapeutic drug levels at restricted sites of infection could be realized through targeted long-acting slow effective release drug delivery systems and by optimizing drug metabolism. For opioid use disorder treatment, new strategies that are well tolerated and increase compliance must be developed. The presentation will highlight the progress our research program has made towards ultra-long-acting therapies with dosing interval goals of once/6–12 months to maximize the effectiveness of HIV, HBV and opioid use disorder treatments.

### Lenacapavir for the Treatment and Prevention of HIV infection

Rooney, J.F., MD, Vice President Medical Affairs, Gilead Sciences, Foster City, CA

Lenacapavir is a novel HIV-1 capsid inhibitor approved in combination with other antiretroviral agents for the treatment of HIV-1 infection in highly treatment-experienced patients who are failing their existing treatment regimens. It can be administered orally or by subcutaneous injection. Several development programs are ongoing looking at lenacapavir for both HIV treatment and HIV prevention in different populations. Several of these programs evaluate lenacapavir as part of long-acting treatment or prevention regimens that can be administered orally or by injection when given weekly, monthly, or with dosing intervals of up to one year. In this talk we will review the various programs, their current stage of development, and potential timelines for product availability.

### The Global Health Sciences Department at Clinton Health Access

Domanico, P.L., PhD., Director, Clinton Health Access Initiative, Raleigh, NC

Dr. Paul L. Domanico, Ph.D., led the Global Health Sciences department at Clinton Health Access (CHAI) to successfully deliver a diverse portfolio of new medicines, diagnostics, and technologies, provided leadership in clinical science and implementation research, and ensured that healthcare is dramatically improved and sustained for people living in low-resource settings. As CHAI’s Global Health Research Fellow, he aims to expand CHAI’s partnerships with universities, industry, governments, NGOs, civil society, donors, and others to identify, nurture, fund, and advance global health science and technology innovations that promise to transform the lives of the people we serve. Dr. Domanico will share case studies across the healthcare continuum, from drug discovery to influencing national health policies. The examples will highlight the importance of inclusive, sustained partnerships and decision-making, robust science, and novel business models to accelerate a global healthcare innovation portfolio.

### Drs Mahendra and Adarsh Kumar Memorial Lecture

Dr. Tsuneya Ikezu, MD, PhD, Professor Mayo Clinic, Jacksonville, FL

Cargo lipids in APOE4 brain derived extracellular vesicles facilitate tau propagation in Alzheimer’s disease.

Alzheimer’s disease (AD) is a neurodegenerative disorder with the APOE ε4 allele as a key genetic risk factor. Brain-derived extracellular vesicles (BDEVs) from AD brains carry tau filaments and promote tau transmission, although the influence of APOE ε4 allele on BDEV-mediated disease progression is untested. We isolated BDEVs from ε3/3 and ε4/4 AD brains and performed biological, lipidomic, and proteomic analyses. ε4/4 BDEVs enhanced tau propagation in aged human MAPT knockin (TauKI) mice, and suppressing neuronal uptake and excitability in induced pluripotent stem cell-derived neurons *in vitro*. Lipidomic analysis revealed increased levels of the pro-inflammatory free fatty acid (FFA)18:2 in ε4/4 BDEVs, correlating with tau pathology. Proteomic analysis showed associations between FFA 18:2 and cell adhesion molecules, particularly neural cell adhesion molecule 1. Targeting the molecule reduced tau accumulation in both ε4/4 AD BDEV-injected and TauKI and in PS19 tau mice, suggesting a potential therapeutic approach for AD.

## Lunch Symposium 3: Sponsored by Fisher Scientific

1. Alzheimer’s and neuroHIV: A comparative analysis of metabolic pathway in postmortem brain tissues. Boustani, AB, MD^1^, Laird, AEL, BS^1^, Ford, MKF, BS^1^, Walter, KCW, BS^1^, Shu, LS^1^, Spencer, MS^1^, Avalos, BA, PhD^1^, Fields, JAF, PhD^1^; ^1^Department of Psychiatry , UCSD, San Diego, CA 92122.
  Introduction: The prevalence of people with HIV (PWH) over the age of 55 is rapidly increasing; however, the effects of aging on HIV neuropathogenesis remain unclear. This study examines changes in glucose metabolism pathways in post-mortem brain tissues from individuals with Alzheimer’s disease (AD) and HIV-associated neurocognitive impairment (NCI). Material and methods: Postmortem brain tissues from the frontal cortex were acquired from the National NeuroAIDS Tissue Consortium (n=24) and the San Diego Alzheimer’s Disease Research Center (n=40). Specimens were homogenized by sonication, then resolved by SDS page, and immunoblotted with antibodies against metabolic proteins. The progression of AD and HIV-associated NCI stratified densitometry data. Results: Glucose transporter levels remained unchanged in both conditions, while monocarboxylate transporter (MCT) levels varied; specifically, monomeric MCT1 levels increased, and MCT2 levels decreased in PWH with NCI, whereas MCT4 levels were unaffected. Proteolytic fragments of the insulin receptor changed across the course of HIV-associated NCI, accompanied by a significant decrease in insulin-degrading enzyme levels in PWH with NCI. Glycolytic enzymes hexokinase and pyruvate kinase M2 showed no change in response to disease severity. Notably, truncated variants of the transcription coactivator PGC-1α increased in PWH with NCI. Discussion: These results highlight shared and distinct mechanisms of metabolic dysfunction in AD and HIV-NCI, providing potential targets for therapeutic interventions.
2. Exploring the effects of chronic oral inoculation of actinomyces meyeri on behavioral and neurobiological changes in B6 mice. Salman, T, PhD^1^, Luo, Z, PhD^1^, Johnson, D, MS^1^, Fitting, S, PhD^2^, Jiang, W, MD^1^; ^1^Department of Pharmacology and Immunology, Medical University of South Carolina, Charleston, SC 29425 ^2^Department of Psychology and Neuroscience, University of North Carolina at Chapel Hil, Chapel Hill, NC 27599.
  Neuropathological changes can be associated with microbial dysbiosis. To date, there are limited studies on cannabis-use altered oral microbiome as well as its impact on neuropathogenesis. Previously, we have identified oral enrichment of Actinomyces meyeri specifically for chronic cannabis smoking in humans. Here, we investigated the impact of A. meyeri on neuropathology in B6 mice. The mice were orally inoculated with A. meyeri (Am), A. odontolyticus (Ao), and N. elongata (Ne) (5x10^7 CFU/time) twice/week for 6 months. Elevated plus-maze (EPM) and open field tests (OFT) were performed to monitor anxiety in mice. Brains were analyzed for neuroinflammatory markers, i.e., IL-1B, IL-6, TNF through qPCR. Brain CD11b+ cells were isolated using MACS and evaluated CD206, iNOS, and Dectin-1 using flow cytometry. RNAScope insitu hybridization was applied with a unique Am 16S RNAscope probe, Iba1, and TMEM119. A. meyeri exposure showed anxiogenic effects in mice by spending more time in the periphery (OFT) and a trending decrease in time spent in the open arm of EPM, increased %Dectin-1 and decreased %CD206 in CD45intermediateCD11b+ brain cell population, and increased IL-1B (n=5) compared to microbiome controls (p<0.05, one-way ANOVA). Using RNAScope, we determined no translocation of A. meyeri and non-region-specific activation of resident microglia but not infiltrated macrophages in the brain (n=4). Findings reveal that chronic cannabis-smoking enriched oral A. meyeri can play a significant role in various neurobiological processes. Supported by R01DA055523.
3. Quantitative proteomics of astrocyte-derived exosomes reveal potential novel biomarkers of HIV-associated neurocognitive impairment. Rosario-Rodriguez, LJ, PhD^1^, Cantres-Rosario, YM, PhD^2^, Hernandez-Alejandro, KS, BS^1^, Tosado-Rodriguez, EL, PhD^3^, Cantres-Rosario, Y, MS^4^, Rodriguez De Jesus, AE, MS^4^, Rodriguez, E, BS^1^, Roche Lima, A, PhD^3^, Melendez, LM, PhD^2^, Wojna, V, MD^1^; ^1^Department of Internal Medicine/Neurology Division, School of Medicine/University of Puerto Rico-Medical Sciences Campus, San Juan, PR 00935 ^2^Department of Microbiology and Medical Zoology, School of Medicine/University of Puerto Rico-Medical Sciences Campus, San Juan, PR 00935 ^3^Bioinformatics and Health Informatics, University of Puerto Rico-Medical Sciences Campus, San Juan, PR 00935 ^4^Translational Proteomics Center, University of Puerto Rico-Medical Sciences Campus, San Juan, PR 00935.
  Astrocytes play a crucial role in HIV-associated neurocognitive disorders (HAND), acting as viral reservoirs and exhibiting impaired autophagy, inflammatory signaling, and mitochondrial dysfunction. Astrocyte-derived exosomes (ADEs) can contribute to neuronal damage and neuroinflammation. In previous studies we found elevated ADEs in the plasma of people living with HIV (PLWH), correlating with reactive oxygen species in HAND. We hypothesized that ADEs from HAND patients contained proteins associated with neuroinflammation and neurodegeneration. Plasma was isolated from HIV-negative donors, PLWH with Normal Cognition (HIV-N), and PLWH Cognitive Impaired (HIV-CI). Exosomes were isolated by ultracentrifugation and ADEs were immunoprecipitated using an anti-GLAST-1 antibody. Quantitative proteomics was performed using TMT Labeling. Results showed that HIV-CI patients had decreased levels of Isoform 2 of Queuine tRNA (Q-tRNA)-ribosyltransferase catalytic subunit 1 (QTRT1) compared to HIV-N patients. Moreover, HIV-CI women had increased levels of Testis-specific Y-encoded-like protein 2 (TSPYL2) and STE20-like serine/threonine-protein kinase (SLK) compared to HIV-CI men. Previous research showed that Qtrt1 knockout mice experienced learning and memory deficits, with females being more affected. Mutations in Tspyl2 are linked to neurodevelopmental disorders involving intellectual disability. SLK maintains inhibitory synapses and is linked to protection against HIV infection in females. We identified proteins in ADEs that represent potential novel biomarkers of HAND.
  Supported by NINDS/R01NS099036/R21NS131061/K22NS118975/K00NS113455, NIGMS/ U54GM133807/P20GM103475, NIHMD/U54MD007600.
4. Structural brain abnormalities and neuropsychiatric symptoms in post-covid condition. Ryan, M, MS^1^, Thomas, J, BS^1^, Wang, J^1^, Liang, H, PhD^1^, Cunningham, E, BS^1^, Ernst, T, PhD^1^, Chang, L, MD^1^; ^1^Department of Diagnostic Radiology and Nuclear Medicine, University of Maryland School of Medicine, Baltimore, MD 21201.
  INTRODUCTION: Post-COVID condition (PCC) may affect up to 10% of US adults and includes neuropsychiatric symptoms. We evaluated whether morphometric differences are found in PCC, if they predict neurobehavioral performance, and if they persist over time. METHODS: 29 PCC and 25 matched Controls completed the NIH Toolbox (NIHTB), PROMIS, and structural T1 MRI on a 3T MR scanner. Seven Controls and 11 PCC returned 2 years later for follow up. Imaging data were processed in Freesurfer using the Desikan-Kiliany atlas for morphometry in 34 cortical and 9 subcortical regions per hemisphere. Baseline group differences were assessed using ANCOVAs with age, sex, and intracranial volume (ICV) as covariates, and age-by-group as an interaction term. Linear mixed effects models evaluated group differences over time with age, sex, group, ICV, and visit as covariates, visit-by-group as an interaction, and subject as a random effect. RESULTS: At Baseline, cortical regions were thicker (n=4) and larger (n=7 areas; n=10 volumes), and subcortical volumes (n=4) were also larger in PCC than Controls. On the NIHTB, PCC reported poorer psychological well-being and motor function than Controls. The two groups performed similarly on the Cognitive Battery. Larger volumes and thicknesses predicted poorer psychological well-being. At follow-up, most regional differences tended to normalize. CONCLUSION: Our findings suggest that, in PCC, neuroinflammatory effects may occur initially after acute infection and underlie neuropsychological complaints. However, these effects may normalize over time.
  Supported by R21NS121615.
5. The effect of estrogen and fentanyl on HIV replication and immune migration across the Blood-Brain Barrier. Basic, M.B., MS^1^, Cruz, P.C., PhD^1^, Andrews, A.A., PhD^1^; ^1^Pathology, Immunology and Laboratory Medicine, University of Florida, Gainesville, FL 32610.
  While anti-retroviral therapy has dramatically reduced HIV-related mortality, people living with HIV experience increased rates of neurocognitive disorders. Unresolved viral replication, blood-brain barrier (BBB) hyperpermeability and neuroinflammation are thought to be underlying factors. Sex differences in HIV acquisition and pathology have been long been observed. Yet, the specific contribution of endogenous or exogenous hormones on HIV replication and pathogenesis remains understudied. Moreover, drugs of misuse are known to exacerbate HIV progression and have sex-dependent effects. However, whether hormones and drugs of misuse independently or synergistically contribute to HIV sex differences remains unknown. Thus, we hypothesized that the combination of 17β-estradiol (E2) and fentanyl will synergistically increase HIV replication and immune migration across the BBB. To test our hypothesis, monocytes and macrophages were treated with 10–600 pg/mL of E2, fentanyl, and the combination. Monocytes were then exposed to HIV, labeled and added to a model of the BBB. The migration of monocytes across the BBB showed differential rates of migrated monocytes treated with E2 and fentanyl. To evaluate whether E2 and/or fentanyl impacted HIV replication, supernatants from macrophages infected with HIV were analyzed via a p24 ELISA. Results showed increased p24 production from the combination of E2 and fentanyl. Overall, our findings contribute to our understanding of the impact of sex differences and combinatory effects with drugs of misuse on HIV progression and viral pathogenesis.Supported by R01DA058536.
6. Cocaine impairs anti-viral response in ipsc-microglia to accelerate HIV infection via sigma-1. Oteju, O^1^, Xu, X^2^, Matt, SM^1^, Emanuel, K^2^, Niu, M^2^, Fox, H^2^, Gaskill, PJ^1^; ^1^Department of Pharmacology and Physiology, Drexel University College of Medicine, Philadelphia, PA 19107 ^2^Department of Neurological Sciences, University of Nebraska Medical Center, Omaha, NE 68198.
  Cocaine (Coc) use is highly prevalent in HIV-infected populations, but the mechanisms by which Coc impacts HIV infection are unclear. We showed that dopamine levels induced by stimulant use increase HIV replication in macrophages and microglia. However, it is uncertain whether Coc acts solely via dopamine or if it has other distinct effects on HIV. To assess this, we inoculated human-induced pluripotent stem cell-derived microglia (iMg) with HIV +/- Coc. We evaluated changes in the percentage of infected iMg as well as p24Gag secretion using high-content imaging and AlphaLISA. Coc increased p24 secretion and the number of infected iMg. These effects persisted even in the presence of antiretroviral therapy. Inhibition of dopamine receptors did not diminish the impact of Coc, but inhibition of sigma-1 receptor (S1R) did block the effect. S1R is a chaperone protein targeted by Coc, and S1R agonists independently increased p24 secretion, suggesting that Coc acts through S1R rather than dopaminergic pathways. Immunofluorescence staining showed increased S1R in p24- cells and reduced S1R in p24+ cells in HIV+Coc cultures and showed that HIV+Coc promotes S1R movement to the nuclear envelope and endoplasmic reticulum. Single-cell RNA sequencing revealed Coc reduces host antiviral and innate response genes e.g. restriction factors, unfolded protein response, etc., suggesting Coc reduces the host response against viral replication. Future studies in mixed culture systems will assess changes in HIV infection dynamics, neuronal/glial health, and function in response Coc.
  Supported by National Institutes of Drug Abuse (R01 DA057337, R61 DA058501).
7. Fentanyl enhances HIV infection of macrophages and microglia. Xiao, QH, PhD^1^, Majid, S^1^, Wang, X, PhD^2^, Khan, S, MS^1^, Andrews, AM, PhD^3^, Ho, WZ, MD, PhD^1^; ^1^Department of Pathology and Laboratory Medicine, Temple University Lewis Katz School of Medicine, Philadelphia, PA 19140 ^2^Center for Substance Abuse Research, Temple University Lewis Katz School of Medicine, Philadelphia, PA 19140 ^3^Department of Pathology, Immunology & Laboratory Medicine, University of Florida, Gainesville, FL 32611.
  Background Illicit fentanyl use has become increasingly prevalent in the United States, particularly among opioid users, and is associated with higher rates of HIV-1 outbreaks in regions with high fentanyl availability. While opioids and their receptors are known to contribute to the immunopathogenesis of NeuroAIDS, fentanyl’s impact on HIV-1 infection in the CNS remains unclear. This study investigates whether fentanyl compromises innate immunity and facilitates HIV-1 infection in human microglia and macrophages. Methods Primary monocyte-derived macrophages (MDMs) and iPSC-derived microglia (iMg) were treated with fentanyl at clinically relevant levels before HIV-1 infection. Viral replication was measured via ELISA (p24) and RT-PCR (Gag). The effects of fentanyl on HIV restriction factors, inflammatory cytokines, and viral entry receptors were analyzed via RT-PCR, Flow Cytometry, and Western Blot. Results Fentanyl significantly enhanced HIV-1 infection in MDMs and iMg, and induced viral replication in chronically infected cells. Mechanistically, fentanyl inhibited IFN-α and HIV-1 restriction factors (APOBEC3G, SAMHD1, IFIT1) while upregulating HIV-1 entry receptors (CD4, CCR5, CXCR4), inflammatory cytokines (IL-1β, TNF-α, IL-6, IL-8), and inflammasome components (NLRP1, NLRP3, IL-18). Conclusion Fentanyl enhances HIV-1 replication in MDMs and iMg by suppressing antiviral defenses and increasing inflammation. These findings suggest the possibility that fentanyl use is a facilitating factor for HIV-1 infection of the CNS and contributes to development of NeuroAIDS. Supported NIH grants (R01 DA051893, DA058536 and MH134402).
8. Optimizing HIV neurotherapy with curcumin adjuvant. Godse, S, MS^1^, Zhou, L, PhD^1^, Sinha, N, BS^1^, Kumar, S, PhD^1^; ^1^Department of Pharmaceutical Sciences, The University of Tennessee Health Science Center, MEMPHIS, TN 38163.
  To address the critical challenge of delivering antiretroviral therapy (ART) across the blood-brain barrier (BBB) for effective HIV treatment, this study investigates curcumin (CUR) as an adjuvant to enhance the brain delivery of elvitegravir (EVG). This research aimed to develop novel therapeutics to optimize ART delivery to CNS reservoirs for neuroHIV management. Our prior findings demonstrated that CUR enhanced EVG intracellular concentrations, improved total antioxidant capacity, and reduced inflammation in U1 macrophages. In vivo studies using BALB/c mice confirmed that CUR, particularly via intranasal (IN) administration, reduced off-target distribution, significantly increased brain drug concentrations, and preserved neural homeostasis. Building on these findings, we evaluated the effects of CUR as an adjuvant in EVG treatment on cognitive and motor functions in the EcoHIV mouse model. The combination of EVG and CUR significantly improved HIV-associated motor deficits, as assessed by the CatWalk test, and partially restored cognitive impairments, including non-spatial short-term memory (NOR) and spatial learning (MWM). Cytokine analysis in EcoHIV mouse brain and plasma revealed that the EVG+CUR combination, particularly via IN route, effectively reduced systemic and CNS inflammation, mitigated HIV-associated DNA damage in brain tissue, and preserved neural homeostasis. This therapeutic approach highlights CUR’s potential as an adjuvant to enhance ART efficacy, offering a promising strategy for managing HIV-associated neurocognitive disorders hallmark of neuroHIV.
  Supported by NIH 1R21MH125670- and AG081140.
9. Sri-47056 a pyrimidine structure-based compound allosterically modulates monoaminergic transporters, attenuating the dysregulation of dopamine and serotonin uptake induced by HIV-1 transactivator of transcription (TAT). Jimenez, AC, PhD^1^, Moukha-Chafiq , O, PhD^2^, Nguyen , T, PhD^2^, Subramaniam, A, PhD^2^, Augelli-Szafran, C, PhD^2^, Chang-Guo , Z, PhD^3^, Zhu , J, MD, PhD^1^; ^1^Department of Drug Discovery, Collage of Pharmacy , University of South Carolina, Columbia, SC 29208^2^Department of Chemistry, Southern Research Institute, Birmingham, AL 35205^3^Department of Pharmaceutical Sciences, College of Pharmacy, University of Kentucky, Lexington, KY 40536.
  We have demonstrated the allosteric interaction of HIV-Tat protein with the dopamine transporter (DAT), norepinephrine (NET), and serotonin transporter (SERT) as the main mechanism to disrupt the monoaminergic transmission in HIV infection, which can be attenuated by a novel allosteric modulator, SRI-32743. This study determined the pharmacological profile of SRI-47056, a novel analog of SRI-32743, with improved solubility and metabolic stability and stabilized dopamine and serotonin levels in iTat-tg mice. SRI-47056 displayed IC50 values to inhibit [3H]dopamine uptake via hDAT of 0.96 ± 0.05 µM; hNET 0.24 ± 0.03 µM and, hSERT 3.79 ± 1.44 µM, showing a partial inhibition of dopamine uptake (Emax value 32.33 to 55.58 %). SRI-47056 partially inhibited [3H]5-HT uptake (IC50=5.40 ± 1.09, Emax=65.85 ± 13.17) and [3H]norepinephrine uptake (IC50=0.06 ± 0.1, Emax=48 ± 12.7). SRI-47056 diminished the cocaine-mediated dissociation of [3H]WIN35,428 binding in hDAT (K-1=0.133 ± 0.036 min-1 and K-1=0.086 ± 0.043 min-1, respectively) and [3H]nisoxetine binding in hNET (K-1=0.087 ± 0.027 min-1 and K-1=0.031 ± 0.003 min-1, respectively) relative to cocaine alone (0.309 ± 0.039 min-1, 0.143 ± 0.011, respectively). SRI-47056 attenuated Tat (8.7 or 17.5 nM)-induced decrease in [3H]DA uptake via hDAT and hNET and [3H]5-HT uptake. These findings suggest that attenuating Tat binding to the monoamine transporters through novel allosteric modulators with minimal effects on physiological changes in monoaminergic transmission preserve neurocognitive function in HIV infected individuals.
  Supported by NIH grants DA035714, DA047924, and DA057866.
10. Myeloid targeted disulfide lipid nanoparticles with enhanced endosomal escape improves HIV-1 excision. Dey, SS, MS^1^, Panja, S, PhD^1^, Chaudhary, BN, MS^1^, Ali, MU, BS^1^, Gorantla, S, PhD^1^, Gendelman, HE, MD^1^; ^1^Department of Pharmacology and Experimental Neuroscience, University of Nebraska Medical Centre, Omaha, NE 68198.
  A cure for HIV remains elusive as the elimination of latent proviral DNA by viral vectors has failed due to vector immunogenicity and poor targeting. In contrast, lipid nanoparticles (LNPs) are promising nanocarriers targeting viral reservoirs, offering greater cargo-carrying capacity and reduced immune responses. However, limitations in endosomal escape remain a challenge. Disulfide-containing ionizable lipid-based LNPs were developed to overcome this issue with enhanced endosomal escape in primary myeloid cells (macrophage, microglia, and dendritic cells) and improved cytosolic delivery. We generated a library of 50 ionizable lipids containing disulfide linkages. Our lead candidate (BA-LNP) was compared against FDA-approved ionizable lipids, MC3 and ALC-0315 for transfection efficacy, toxicity in primary monocyte-derived macrophages (MDMs) and biodistribution in NSG humanized mice. Our lead LNP formulation was nontoxic and demonstrated a 20-fold higher mRNA transfection efficiency in MDMs than MC3 and ALC-0315, which are limited by poor endosomal escape in myeloid cells. The BA-LNP biodistribution was observed explicitly in the humanized mice’s lymphoid and pulmonary tissues. CCR5 targeted BA-LNP achieved over 80% excision of HIV-1 proviral DNA compared to non-targeted LNPs in MDMs, a clear advancement over conventional formulations. In conclusion, Disulfide-based LNPs offer a significant advantage in overcoming endosomal escape barriers in MDMs, facilitating effective CRISPR-Cas9 delivery to HIV-1 reservoirs.
11. Incomplete glial recovery despite ART: Insights from scrnaseq in a triple humanized glial mouse model of HIV brain infection. Kondeti, S, PhD^1^, Makarov, E, MS^1^, Theile, M, BS^1^, Dutta, D, PhD^1^, Gorantla, S, PhD^1^; ^1^Pharmacology and Experimental Neuroscience, University of Nebraska Medical center, Omaha, NE 68105.
  Central nervous system (CNS) remains a sanctuary for HIV reservoirs even with effective antiretroviral therapy (ART), complicating recovery of CNS homeostasis. Understanding CNS HIV reservoirs and their role in neuroinflammation is limited by the lack of suitable models. We developed a novel triple humanized (Tri-HU) mouse model with human immune system, microglia, and astrocytes to examine CNS viral reservoirs and glial activation. Human microglia, astrocytes, and lymphocytes were isolated from Tri-HU mouse brains (uninfected, HIV-infected, HIV+ART). Single-cell RNA sequencing (scRNA-seq) assessed HIV copies and glial activation. Trajectory analysis compared glial activation across infection stages. HIV in different brain cells was analyzed by read alignment to the HIV genome. scRNA-seq revealed that uninfected brains were rich in homeostatic microglia. In contrast, HIV infection caused a dramatic shift, reducing homeostatic microglia to ∼5% and increasing inflammatory microglia to ∼95%. ART treatment significantly reversed these changes, restoring homeostatic microglia to ∼87.3% and reducing inflammatory microglia to ∼12.7%. Distinct activation states were observed across groups, among them we identified complete and defective HIV transcripts. Interestingly, human astrocytes harbored a significant amount of defective HIV transcripts. ART reduces HIV and inflammation in the CNS but does not eliminate infection, leading to persistent neuroimmune disruptions. The Tri-HU model provides a platform for studying glial infection, inflammation, and HAND, supporting therapy development. Supported by 1R21MH131220-01, NIH/NIDA 1R01DA054535-01, NIH/NIMH 1 R01 MH128009-01.
12. Effects of methamphetamine (meth) on HIV integration and latency in a cellular microglia model. Lim, WL, PhD^1^, Delorme-Walker, V, PhD^1^, Au, K, BS^1^, Lusic, M, PhD^2^, Fox, H, MD, PhD^3^, Marcondes, MCG, PhD^1^; ^1^Neuroimmunology, San Diego Biomedical Research Institute, San Diego, CA 92121 ^2^Center for Integrative Infectious Diseases (CIID), Heidelberg University Hospital, Heidelberg, 69120 ^3^Department of Neurological Sciences, University of Nebraska Medical Center, Omaha, NE 68198.
  Meth use is commonly associated with exposure to HIV and is prevalent among people living with HIV. Meth use aggravates the adverse effects of HIV in the CNS, where latent HIV reservoir cells present a challenge to eradicate infection. The complex mechanisms by which Meth impacts HIV target cells are further complicated by the dopaminergic neurotransmission involved in substance use and the fact that microglia and other HIV target cells express dopamine (DA) receptors. Understanding the contribution of DA to the dynamics of proviral integration and chromatin organization associated with HIV latency in CNS cellular reservoirs is critical to address the challenges of cure under conditions of substance use. Here, we used a C20 microglia cell line infected with vesicular stomatitis virus glycoprotein (VSV-G) pseudotyped with a dual fluorescence HIV reporter (HIVGKO) that allows visualization of active and latent infection, to understand the effects of Meth and DA on latency and its characteristics. The percentage of active and latently infected C20 cells, HIV integration and secreted p24 levels were altered by DA treatment in a dose-dependent manner. DA also modified the expression and genomic distribution of CTCF and active chromatin marker H3K36me3, previously linked to HIV integration and enriched at susceptible sites found in topologically associating domain (TAD) boundaries. The results suggest that DA may impact epigenetic characteristics underlying inflammation and chromatin susceptibilities constituting the principles guiding HIV integration. Supported by NIDA R61DA059924 to MCGM/HF.

### Plenary Talk

Jonathan Rockman, Behavior Health and Social Service Providers, Addiction disorders, Omaha, NE

#### Counseling the opioid crisis and withdrawal syndrome

Mr. Rockman will speak on the opioid epidemic. This includes prescription pain relievers to illegal drugs like heroin. We will talk about counseling experiences with a focus on the rise in abuse of opioids. We will also speak about experiences in withdrawal symptoms and treatments to help manage opioid withdrawal and alcoholism and in combating this crisis at an individual level.

## Symposium 4: Development and Delivery of Diagnostic and Therapeutic Biomarkers to the Brain

### Multi-modality imaging for precision delivery of cells and biologics to the brain

Walczak, P, MD, PhD^1^; ^1^Program on Image-Guided Neurointerventions, School of Medicine, University of Maryland Baltimore, Baltimore, MD

We are witnessing unprecedented advancements in precision therapeutics, spanning small molecules, antibodies, gene therapeutics, and cell-based therapies. Among these, biologics have demonstrated exceptional potency, yet their large size poses significant delivery challenges, particularly for difficult-to-reach organs such as the brain. Systemic administration is the preferred route due to its ease of use, but blood-organ barriers severely limit drug penetration, with only a fraction of a percent reaching the brain. My research focuses on overcoming these delivery challenges through intra-arterial administration and multi-modality, multi-scale imaging to optimize therapeutic access. Interventional imaging during IA infusion enables real-time verification of biodistribution, with MRI providing global accumulation data, bioluminescence imaging assessing cell viability, and intravital microscopy visualizing cellular diapedesis into the brain parenchyma. While IA administration enhances targeting, its complexity limits feasibility for repeated dosing. To address this, we leverage genetic engineering to develop cell-based factories capable of producing and releasing therapeutics locally. This approach has been applied to HIV therapy, where engineered cells produce HIV-neutralizing antibodies and neuroprotection in stroke and TBI, where engineered cells produce P2X7-targeting nanobodies to modulate inflammation. By integrating precision delivery, advanced imaging, and bioengineering, our research aims to enhance the therapeutic potential of biologics for brain disorders. Supported by R01DA056739.

### From seaside to bedside: bioengineering proteins for diagnostics and treatment

Gilad, AA, PhD^1^; ^1^Dept. Chemical Engineering, Michigan State University, East Lansing, MI

Recent advancements in DNA technologies present promising opportunities for developing innovative tools in treatment and diagnostics. These new tools integrate synthetic and semi-synthetic genes, effectively providing instructions for protein production. Remarkably, these proteins can monitor cell health without invasive procedures and influence cell behavior from a distance. Inspired by marine organisms, we are exploring technologies based on split proteins that can monitor changes in neurotransmitters by emitting light. Moreover, this light can be harnessed to activate a gene sequence, allowing for the modulation of cells at the molecular level. To ensure harmony with cellular signaling pathways, we have developed synthetic enzymes—referred to as magnetozymes—that can be activated remotely using an electromagnetic field when expressed in cells. Within the magnetozyme core, an ‘antenna-like’ three-phenylalanine motif, which we have cloned and characterized from an Actinopterygii protein, is essential for the magnetoreception response. These genes can not only serve as part of a reporter system but can also initiate enzymatic reactions. Collectively, this toolkit of semisynthetic genes can be utilized to explore and potentially enhance the interactions between the immune and nervous systems. This work may assist in optimizing the use of pharmacological agents and paving the way for future cures. We believe that these modest advancements can significantly contribute to the field and improve outcomes in healthcare.

Supported by NIH R01-EB031008; R01-EB030565; R01-EB031936

### Intra-arterial route with osmotic blood-brain barrier opening for enhanced drug delivery to the brain

Chu, CC, MD^1^, Lesniak, LW, PhD^2^, Janowski, MJ, MD, PhD^1^, Walczak, P, MD, PhD^1^; ^1^Dept of Diagnostic Radiology & Nuclear Medicine, University of Maryland School of Medicine, Baltimore, MD 21201 ^2^Department of Radiology, UT Southwestern Medical Center, Dallas, TX 75390.

The blood-brain barrier (BBB) is a highly selective permeability barrier, preventing the entry of harmful substances from the peripheral circulation into the brain. However, the BBB is also a major obstacle for effectively delivering therapeutics into the brain. We first developed a predictable, reproducible and safe mannitol-based BBB opening (BBBO) technique in mice under real-time MRI guidance. This experimental platform was then exploited for targeted intra-arterial delivery of therapeutics including antibodies and nanobodies to the brain. PET imaging revealed a significant advantage in brain accumulation for antibodies and nanobodies after IA delivery in contrast to IV administration. Prior IA BBBO further enhanced this superiority. Dynamic intravital imaging at a microscopic level was performed further to investigate the mechanism of BBBO and drug extravasation. As a result, the antibody leakage upon BBBO was visualized during IA infusion. The antibody penetrated the brain parenchyma rather than being captured at the level of capillary vessels. Overall, our study provided an important platform for exploring the biology of BBB and demonstrated the distinct advantage of IA route with BBBO for robust brain uptake of therapeutics. Supported by NIH R01DA056739, R01NS120929 and 2024-MSCRFD-6348

### Evaluation of brain injuries and neuroinflammation with PET imaging

Lesniak, W., PhD^1^, Martin G. Pomper, MGP, MD, PhD^1^, Jennifer M. Coughlin, JMC, MD^1^; ^1^Department of Radiology, UT Southwestern Medical Center, Dallas, TX

Traumatic brain injuries (TBI), ischemic strokes, and chronic neurodegenerative disorders are associated with the activation of glial cells promoting neuroinflammation. Positron emission tomography (PET) plays a critical role in understanding central nervous system pathologies. Translocator protein (TSPO) is a well-validated and widely used biomarker of neuroinflammation. We have used [11C]-N,N-diethyl-2-[2-(4-methoxyphenyl)-5,7-dimethyl-pyrazolo[1,5-a]pyrimidin-3-yl]-acetamide ([11C]DPA-713) PET radiotracer to investigate whether localized brain injury, as indicated by elevated TSPO levels, is linked to sports-related TBI.[1] NFL players and non-collision sport athletes (swimmers) were evaluated in terms of regional [11C]DPA-713 distribution volume measured by PET, regional brain volumes via MRI, and neuropsychological assessments. The study revealed that former NFL players exhibited significantly higher TSPO levels in multiple brain regions compared to swimmer, notably in the cingulate and frontal cortices, and hippocampus. NFL players performed worse in learning and memory tasks. This study indicates ongoing neuroimmune activation in NFL players long after retirement from the sport and highlights the need for further studies to explore the relationship between neuroinflammatory and neuropsychological symptoms. However, due to the lack of cell-type specificity of TSPO, PET imaging of CSF1R with [11C]CPPC may provide better understanding of neuroimmune responses at the cellular level in TBI.[2] 1- JAMA Netw Open, 2023;6(10):e2340580 2- EJNMMI Res, 2022;12(1):64. Supported by NS100847, 5R21MH082277, 5R01MH092443, R01EB012547, ES007062, 5T32EB006351, P50AG005146, the Lupus Foundation for America, NFL

## Symposium 5: Assessing the Blood Brain Barrier in Neurological Disorders and Inflammation

### Signatures of Blood Brain Barrier Disruption in People with HIV and their Associations with Neurobehavioral Outcomes and Cannabis Use Patterns

Ludicello, J., PhD,^1^ ^1^University of California San Diego

People living with HIV (PLWH) remain vulnerable to central nervous system complications despite antiretroviral therapy (ART) that suppresses viral replication. While many etiologies of these complications exist, damage to the blood-brain-barrier has been implicated but not thoroughly characterized. The prevalence of PLWH who use cannabis is greater than that of the general population and recent evidence by our group and others indicates that cannabis may protect PWH from BBB damage by reducing inflammation. Here we will present data across a series of studies aimed at characterizing BBB dysfunction in PWH using cerebrospinal fluid (CSF) and blood biomarkers (e.g., VCAM-1, tight junction proteins, CSF-serum albumin ratio), and investigating the complex relationships between BBB dysfunction, neurobehavioral consequences (e.g., neurocognitive impairment [NCI]), and cannabis use. Our current findings highlight BBB alterations in PWH that are linked to NCI, with evidence of both beneficial and adverse effects of cannabis use depending on patterns of use (e.g., frequency, amount, recency). We will also present new preliminary data from an active study examining the effects of HIV and cannabis use on BBB disruption using advanced BBB neuroimaging (dynamic contrast-enhanced MRI, restriction spectrum imaging). Findings may provide insight into the mechanisms of BBB dysfunction in PWH as well as thresholds at which cannabis use may confer adverse or beneficial effects, with the goal of ultimately informing therapeutic strategies to ameliorate BBB dysfunction and associated neurobehavioral consequences in PWH.

### Stroke vulnerability in the context of HIV-infection

Torices, S., PhD, Miami Miller School of Medicine, Miami, FL

HIV infects CD4+ cells in the bloodstream, enabling the virus to bypass the blood-brain barrier (BBB) and enter the brain, where it accumulates. This leads to neuroinflammation and the onset of cerebrovascular conditions like ischemic stroke. Among the various cells in the central nervous system (CNS), pericytes have been identified as significant targets for HIV infection. Disruption of the BBB’s structure and function is a hallmark of brain infection caused by HIV. Importantly, BBB damage in HIV infection has been linked to changes in the expression of tight junction (TJ) proteins, including occludin. In a model of ischemic stroke using occluding deficient mice, we explored the functional role of occludin in cerebrovascular health during HIV infection. Our findings show that occludin regulates HIV infection by promoting the expression of antiviral interferon-stimulated genes (ISGs) and genes involved in the RIG-I signaling pathway, impacting mitochondrial bioenergetics and apoptosis in human brain pericytes. We also present new evidence showing that the absence of occludin worsens ischemic stroke outcomes in HIV-infected brains. Together, our results position occludin as a crucial regulator of both innate immune responses and HIV infection. Additionally, our study highlights occludin as a key factor in managing cerebrovascular diseases such as ischemic stroke, suggesting it as a potential therapeutic target for these conditions.

### The protective role of microglia to the Blood Brain Barrier in Aging

Milner, R., MD, PhD, San Diego Bimedical Research Institute, San Diego, CA

We have shown that chronic mild hypoxia (CMH) accelerates clinical recovery in an animal model of Multiple Sclerosis (MS), leading to long-term stable reductions in disease severity. Significantly, this protective effect of CMH was attributed to enhanced BBB integrity and accelerated cell death of infiltrated immune cells. Ongoing studies are exploring ways of using this information to generate new approaches for treating MS. We will be showing the impact of hypoxia on transient vascular breakdown, and a novel protective role for microglia in preventing hypoxia-associated transient vascular disruption, both in the brain and in the spinal cord.

### Effects of modern era antiretrovirals on the functional integrity of the Blood Brain Barrier in Methamphetamine users and implications to pharmacokinetics

Delorme-Walker, V, PhD^1^, Lim, A, PhD^1^, Au, K, BS^1^, Furihata, T, PhD^2^, Siuzdak, G, PhD^5^, Webb, W, PhD^5^, Montoya, J, MD, PhD^3^, Ludicello, J, PhD^4^, Grelotti, D, MD, PhD^3^, Milner, R, MD, PhD^1^, Marcondes, MCG, PhD^1^; ^1^Neuroimmunology Program, San Diego Biomedical Research Institute, San Diego, CA, ^2^Laboratory of Clinical Pharmacy & Experimental Therapeutics, School of Pharmacy, Tokyo University of Pharmacy and Life Sciences., Tokyo. ^3^Owen Clinic, University of California San Diego, San Diego, CA. ^4^HIV Neurobehavioral Research Program, University of California San Diego, San Diego, CA. ^5^Center for Metabolomics and Mass Spectrometry, Scripps Research, La Jolla, CA.

The suppression of HIV critical to control viral spread remains a challenge among Methamphetamine (Meth) users, who are more likely than non-users to have detectable viral load. As neurological consequences of HIV remain in ART suppression, it is critical to understand effects of ART combined with Meth on underlying factors such as Blood-Brain Barrier (BBB) disruption. In vitro BBB models with cerebrovascular endothelial cells, astrocytes and pericytes, and a HIV-1 latent cell environment, were exposed to Meth and/or ART, and assessed to permeability to dextran, expression of tight junction proteins, transendothelial electrical resistance and transport of ART drugs using LC-MS/MS. We tested compounds in oral Biktarvy (bictegravir, emtricitabine and tenofovir alafenamide) and in the long-acting injectable formulation Cabenuva (cabotegravir and rilpivirine), at plasma or CSF concentrations. Results: Biktarvy drugs caused dose-dependent increase in permeability without HIV, with predicted higher concentrations across the BBB. Cabenuva compounds also increased permeability in controls, but improved integrity in the context of HIV and Meth, with predicted lower amounts crossing the endothelial barriers. Conclusion: Current ART formulations may be damaging to the BBB in controls, yet benefits to integrity were observed in conditions emulating HIV and Meth. The findings suggest that 1) the use of these drugs in prophylaxis may have implications to neuropathogenesis, and 2) despite ART benefits to the BBB, access to the brain may be impaired by HIV and Meth.Supported by NIDA 1R01DA059344

### Alcohol and e-cigarette exposure induce release of extracellular vesicles and their plasminogen urokinase content promoting blood brain barrier injury (BBB)

Mekala, N., PhD, Togre, N., Rom, S., Sriram, U., Persidsky, Y., Department of Pathology and Laboratory Medicine, Lewis Katz School of Medicine, Temple University, Philadelphia, PA 19140, USA.

Previously, we demonstrated that chronic exposure to alcohol (EtOH) and e-Cig stimulates P2X7 receptor (P2X7r) leading to extracellular vesicle (EV) release from human pulmonary alveolar epithelial cells (PAEC). The amount and content of EVs depended on the stimulants. Here, we assessed EV content by proteomic analysis. We evaluated EV paracrine signaling in human brain microvascular endothelial cells (BMVEC), which was correlated with changes in isolated brain microvessels from mice chronically exposed to alcohol (EtOH). EVs were isolated from media of PAEC exposed to EtOH (100mM), aldehyde ALD (100µM), and e-Cig conditioned media (1.8% nicotine). Proteomic analysis demonstrated a significant increase in several proteins, notably in plasminogen urokinase activator (PLAU), known to break the extracellular matrix (ECM). Paracrine signaling capability of EVs was shown in BMVEC by decreased trans-endothelial resistance (TER, measuring barrier function) and intracellular Ca^2+^ release. Pretreatment of PAEC with P2X7r inhibitor led to decreased PLAU content in EVs, TER amelioration and diminished Ca^2+^ release in BMVEC. A significant increase of PLAU, MMP 2, MMP9 gene expression and lower TIMP1 expression in microvessels confirmed BBB damage *in vivo*. Animal treatment with P2X7r inhibitor resulted in normalization of PLAU, MMP2, MMP9 and TIMP1 gene expression in isolated brain microvessels. *In toto* PLAU transported by PAEC derived EVs can act as paracrine signal and negatively affect the BBB integrity confirming lung-brain cross talk during EtOH and e-Cig exposure and regulatory role of P2X7r.

## Symposium 6: Neuroinflammation in the reward pathway of addiction

### Neuroinflammatory signatures in the reward pathway following brain injury suggest vulnerability to an enhanced addiction phenotype.

Ramirez, S.H., PhD^1^; ^1^Department of Pathology, Immunology and Laboratory Medicine, University of Florida, College of Medicine, Gainesville, FL 32610.

Recent epidemiological data suggests that individuals sustaining adolescent brain injuries may be at greater risk for substance use/misuse problems. To better understand the above pathological relationship, we used preclinical animal models and showed that brain injury during adolescence exacerbated the reinforcing properties of psychostimulants and opioids in adulthood. Importantly, our results revealed unique inflammatory outcomes in the mesolimbic and corticostriatal pathways during the chronic phase that follows blunt force trauma. Overall, these analyses offer key insights into the link between traumatic brain injuries during adolescence and addiction liability later in life.

Supported by Shriners Hospitals for Children, The Pennsylvania Department of Health.

### Chronic nicotine use suppresses accumbens immune signaling

Gipson, C.D., PhD^1^, White, A., BS^1^, Richie, D.L., PhD^1^, Corley, C.L., PhD^1^, Scofield, M.D, PhD^2^; ^1^Department of Pharmacology and Nutritional Sciences, University of Kentucky, Lexington, KY 40536^2^Department of Anesthesiology, Medical University of South Carolina, Charleston, SC 29425.

Smoking is a leading preventable cause of death, and nicotine is the primary substance responsible for maintaining smoking behavior. We have repeatedly shown that nicotine self-administration (SA) modulates glutamate signaling within a key region of the brain reward pathway, the nucleus accumbens core (NAcore), and dysregulations in NAcore glutamate signaling are critical in driving nicotine-related behavior. The neuroimmune system plays a vital role in regulating glutamate signaling; thus, neuroimmune and glutamate signaling are intricately linked. However, nothing is known regarding how chronic nicotine use may impact the neuroimmune-glutamate penta-partite synapse within the NAcore. Thus, this study determined the consequences of volitional nicotine use via a rat SA paradigm, impacts NAcore cytokine and chemokine expression, and changes in microglia morphometrics (the brain’s resident immune cell). We show that in female rats, chronic nicotine SA (15 sessions) suppressed pro-inflammatory cytokines, with TNFalpha being the most suppressed, but increased expression of the fractalkine (CX3CL1), a neuronal “help me” signal. Further, nicotine SA increased microglial cellular complexity, indicating a suppression of their ability to react to injurious stimuli. Together, our results suggest that volitional nicotine use results in neuroimmune suppression, which could regulate NAcore glutamate dyshomeostasis underlying nicotine use motivation. Ongoing studies are evaluating the necessity of microglia in driving nicotine use via chemogenetics using CX3CR1-cre rats. Supported by NIDA 046526, NIDA 061626.

### HIV-1 and/or opioid use disorder induce functional alterations in microglia

McLaurin, KA, PhD^1^, Nelson, KJ, BS^1^, Veenstra, BJ, BS^1^, Grayson, AM, BS^1^, Elder, TR, BS^1^, Turner, JR, PhD^1^; ^1^College of Pharmacy, University of Kentucky, Lexington, KY 40503.

Independently, HIV-1 and opioid use disorder (OUD) induce profound neurocognitive impairments (NCI); the prevalence and severity of which are exacerbated following comorbid HIV-1 and OUD. Microglia, the innate immune cells in the central nervous system, play a critical role in maintaining brain homeostasis via surveillance, phagocytosis and modulation of neural circuits. Given that microglia are productively infected by HIV-1 and contain opioid receptors, functional alterations in microglia are uniquely positioned to underlie HIV-1 and/or opioid-induced NCI. Three complementary experiments have been undertaken to systematically evaluate how HIV-1 and/or OUD alter proliferation and phagocytosis in microglia. First, in vitro techniques (n=4/group) were utilized to assess microglia proliferation following chimeric HIV (EcoHIV) exposure. Preliminary results support that HIV sex-dependently alters microglia proliferation and associated ligands (e.g., CSF1R, DAP12) evidenced via polymerase chain reaction and in situ hybridization. Second, the dose-dependency of oxycodone (0, 2, 5, or 10 mg/kg), a prescription opioid, on microglial function in the prefrontal cortex (n=6/group) is being examined. Finally, ongoing in vivo experiments are being conducted to evaluate how EcoHIV inoculation following a history of oxycodone self-administration alters microglia function (n=12/group). Collectively, these studies will establish HIV-1 and/or OUD induce functional alterations in microglia heralding future studies evaluating this as a pathophysiological mechanism underlying NCI. Supported by DA056288, GM130456.

### Targeting neuroinflammatory signaling in the brain for the reduction of opioid-induced phenotypes

Daws, S, PhD^1^, Floris, G, PhD^1^, Dabrowski, K, PhD^1^, Zanda, M, PhD^1^; ^1^Center for Substance Abuse Research, Temple University, Philadelphia, PA 19422.

Deaths associated with opioid misuse remain a significant public health concern and research into molecular mechanisms of opioid seeking is essential for identification of new treatment strategies to reduce opioid use. Transcriptomic studies from human postmortem tissue have highlighted dysregulation of inflammation-related pathways in the nucleus accumbens (NAc), a brain region critical for drug reward and reinforcement, as key neuroadaptations that result from chronic opioid use, suggesting further investigation into the contribution of neuroimmune activation to opioid-induced phenotypes is warranted. We hypothesized that immune stimulation before opioid exposure may recapitulate human opioid use disorder (OUD) transcriptome changes in the NAc and alter behavioral responses to the opioid heroin. We tested this hypothesis by treating rats chronically with the immunostimulant lipopolysaccharide (LPS) before heroin exposure. History of LPS amplified heroin-induced locomotor sensitization, demonstrating that immune activation regulates behavior responses to heroin. Using RNA-sequencing, we profiled the NAc in rats that received LPS and heroin. We identified significant overlap of gene expression patterns in rats treated with LPS/heroin compared to published human NAc OUD datasets. We conclude that immune activation before opioid exposure recapitulates a unique signature of the NAc OUD transcriptome that can be further explored in preclinical models. Supported by NIDA/P30DA013429.

## Symposium 7: Translational Science

Translational science is a new field that seeks to improve health by translating recent scientific discoveries into practical applications. It is a central component of medical science and biology.

Presentations will include:

– Translational science for barriers to research progress
– New technologies to overcome developmental barriers
– Facilitate research activities in making the process more efficient
– Developing critical collaboration between researchers and beyond

## Symposium 8: Neuroglia interaction in the context of HIV and/or drugs of use/misuse

### HIV-1 Infection of Brain Organoids and METH Exposure Dysregulate Homeostatic Microglia-Neuron-Astrocyte Interactions by Unique Pathways

Sreeram, S., MS^1^, Chen, Y, PhD^2^, Leskov K.S., PhD^1^, Eum, J.^2^, Bury, L., PhD^2^, Ye, F., PhD^1^, Garcia Mesa, Y., PhD^1^, Wynshaw-Boris, A., PhD^2^, Karn, J., PhD^1 1^Department of Molecular Biology and Microbiology, Case Western Reserve University, Cleveland, Ohio; ^2^Department of Genetics and Genome Sciences, Case Western Reserve University, Cleveland, Ohio

We developed a unique human iPSC-derived 3D brain organoid model to study interactions between microglia and other brain cells. The organoids were developed by coculturing neural progenitors (NPCs) with tdTomato-tagged CD34+ microglia precursors. Once microglia had developed by day 15, the organoids were infected with macrophage R5-tropic NL-AD8 HIV-1. In parallel experiments, organoids at Day 25 were treated with water or METH (100 nM, 72 hours). Single-cell transcriptomics (scRNA-seq) revealed gene expression changes and the impact of HIV infection or METH on neuron-astrocyte-microglia interactions. HIV-1 productively infected and spread within the tdTomato+ microglia population, upregulating interferon genes while reducing homeostatic genes. Interferon signals induced by HIV infection upregulated MHC antigen presentation, stress and repair genes in neurons. By contrast, METH altered cytoskeletal, ion-transport pathways, lipid metabolism genes in neurons and astrocytes. Ligand-receptor analysis in METH organoids revealed fibroblast growth factors, neurexins, and neuroligins modulate ion channels and synaptic connections in neurons. At the same time, amyloid genes, CX3CL1, and S100 ligands enhanced chemokine gene expression in microglia. Our model shows cerebral organoids with microglia are a valuable tool to investigate the effects of HIV infection and METH. While HIV-induced reactive microglia, METH-induced neuroinflammation, astrogliosis, and neuronal excitability, priming them towards neurodegeneration that could be exacerbated in the presence of HIV.

### Multiple receptor-mediated effects of fentanyl and xylazine on neural activity of striatal medium spiny neurons

Yarotskyy, V., PhD^1^, Nass, SR, PhD^1^, Hahn, YK, PhD^2^, Contois, L, PhD^1^, McQuiston, AR, PhD^2^, Knapp, PE, PhD^2^, Hauser, KF, PhD^1^; ^1^Department of Pharmacology and Toxicology, School of Medicine Virginia Commonwealth University, Richmond, VA ^2^Department of Anatomy and Neurobiology, School of Medicine Virginia Commonwealth University, Richmond, VA

The use of mixed fentanyl, a µ-opioid receptor (MOR) agonist, and xylazine, an α2-adrenoceptor (α2AR) agonist, results in multiple comorbidities with poorly understood mechanisms. We used electrophysiological recordings from striatal medium spiny neurons (MSNs) to assess the alterations in MSN firing caused by fentanyl and xylazine. In neuronal and mixed-glial co-cultures, 100 nM fentanyl reduced the spontaneous firing frequency. Overnight exposure to fentanyl reduced severely the fraction of MSNs with spontaneous action potentials. This effect was not sensitive to MOR antagonist naloxone (10 µM) but entirely negated by co-administering the α1 adrenoceptor (α1AR) inverse agonist prazosin (100 nM) and partially reversed by α1A/C adrenoceptor antagonist RS 100329 (300 nM). In striatal brain slices, acutely applied fentanyl caused nominal changes in firing rates of dopamine type 1 or type 2 receptor- (D1 or D2) -expressing MSNs, but promoted the firing adaptation in D2 MSNs only. Prolonged (2–5 h) fentanyl application reduced firing rates in both D1 and D2 MSNs. At the same time, xylazine alone (10 µM) reduced firing rates in D2 MSN at strong stimuli, fentanyl and xylazine mixture sensitized and further reduced them by likely xylazine interactions with α2AARs. Immunocytochemical and in situ hybridization data confirmed the presence of α1ARs in some astroglia and neurons subpopulations and the presence of α2ARs in the striatum. We propose that fentanyl and xylazine alter MSN activity via a complex mechanism involving their interactions with MOR, α1ARs, and α2ARs in neurons and glia. Supported by NIH/NIDA R01 DA060724, R01 DA057346, R01 DA045588, R21 DA057153, F32 DA053163, and K99 DA059324

### HIV-TAT dysregulates microglial lipid metabolism through SREBPr-124 axis: implication of lipid droplet accumulation in microglia in neuroHIV

Guo, Minglei, PhD^1^, Cheng, Yan^1^, Jung, J^1^, Shuboni-Mulligan, DD^1^, Chen, JF^2^, Hu, W^3^; ^1^Biomedical and translational sciences, Eastern Virginia Medical School/VHS, NORFOLK, VA ^2^Center for Craniofacial Molecular Biology, USC, Los Angeles, CA ^3^Department of Anatomy and Neurobiology, Virginia Commonwealth University, Richmond, VA.

Chronic HIV infection can dysregulate lipid/cholesterol metabolism in the peripheral system, contributing to the higher incidences of diabetes and atherosclerosis in HIV-1 infected individuals. Recently, accumulating evidence indicates that HIV proteins can also dysregulate lipid/cholesterol metabolism in the brain and such dysregulation could be linked with the pathogenesis of HIV-associated neurological disorders (HAND)/NeuroHIV. To further characterize the association between lipid/cholesterol metabolism and HAND, we employed HIV-inducible transactivator of transcription (iTAT) and control mice to compare their brain lipid profiles. Our results reveal that HIV-iTAT mice possess dysregulated lipid profiles and have increased lipid droplets (LDs) accumulation microglia (LDAM) in the brains. HIV protein TAT can upregulate LD formation by enhancing lipid/cholesterol synthesis in vitro. Mechanistically, HIV-TAT increases the expression of sterol regulatory element-binding protein 2 (SREBP2) through microRNA-124 downregulation. Cholesterol synthesis inhibition can block HIV-TAT-mediated NLRP3 inflammasome activation and microglial activation in vitro and mitigate aging-related behavioral impairment and memory deficiency in HIV-iTAT mice. Taken together, our results indicate an inherent role of lipid metabolism and LDAM in the pathogenesis of NeuroHIV (immunometabolism). These findings suggest that LDAM reversal through modulating lipid/cholesterol metabolism could be a novel therapeutic target for ameliorating NeuroHIV symptoms in chronic HIV-1 infected individuals.

### Interactions between neurons and astrocytes during the pathogenesis of HIV-associated pain

Tang, SJ, PhD^1^; ^1^Department of Anesthesiology, Stony Brook University, Stony Brook, NY

Chronic pain is a common neurological disorder of HIV patients. Astrogliosis is a prominent neuropathology identified in the pain pathway of HIV patients. However, the pathogenic contribution of reactive astrocytes is unclear. We have used interdisciplinary approaches to determine the role of astrocytes in mouse models of HIV-associated pain. Our results reveal bidirectional interactions between neurons and reactive astrocytes in the pain neural circuits in the spinal cord dorsal horn of the model. Supported by R01NS079166, R01DA036165, R01NS095747.

## Symposium 9: Nanopharmacology: Nanotechnology based therapeutics for Infection and Inflammation

### Nanotechnology approach to target Neuroinflammation

Mahajan, S.D., PhD, Associate Professor, Department of Medicine, Jacobs School of Medicine & Biomedical Sciences, University at Buffalo, Buffalo, NY

Evidence suggests neuroinflammation as a standard feature of most neurological disorders and is potentially the primary cause of the disease or a response to neurological dysfunction. Targeting cells and molecular pathways that contribute to neuroinflammation using innovative nanotechnology-based strategies can facilitate the treatment of neurological disorders. Given the complexity of cellular and molecular phenomena involved in neuroinflammation, cell-targeted pharmacological approaches are essential in designing effective treatments. Recent advances in nanomaterials for drug delivery to the CNS and state-of-the-art nanoparticle drug delivery platforms can significantly impact local CNS bioavailability of pharmacological compounds and help treat neurological diseases. The research work presented will highlight the development of various nanotechnology platforms that we use to target neurological diseases such as Traumatic brain injury, Opiate addiction, and Alzheimer’s disease. Understanding neuroinflammatory pathways and CNS cellular biology has helped us develop methods to modify nanoparticle surface with ligands that increase cell-specific targeting in the brain and increase BBB penetration.

### 3D Nano-Imaging at the Sub-Cellular, Cellular and Multicellular Levels

Khmaladze, A., PhD, Associate Professor, Department of Physics, SUNY Albany, Albany, NY

In this presentation, I will discuss the applications of phase imaging techniques, namely Digital Holographic Microscopy (DHM) and Transport of Intensity Equation (TIE), together with fluorescent and Raman spectroscopic imaging to cellular apoptosis, mapping of intracellular iron, and ferroptosis. The non-invasive nature of optical microscopy enables us to study various materials under conditions approaching or similar to their “natural” environment. This is especially relevant to live biological specimens, which can be studied both in vitro and in vivo, providing a unique insight into the dynamic processes occurring in the live organisms. In recent years, the emphasis has been shifting towards the technologies that combine several different techniques to study a particular biological system, which in turn allows measuring a partially overlapping set of parameters, leading to more profound understanding of processes occurring within that system. Methods of tracking morphological cell changes are based on measurements of phase, which is proportional to the cell thickness and can be extended to measure the optical path length, and, therefore, cell volume. Additionally, Raman micro-spectroscopy is widely used for non-invasive and non-destructive mapping of chemical composition within live biological samples, such as cells, organoids, and tissues.

### Laser ablation – a unique methodology to nanoformulate HIV antivirals

Singh, A; Kutscher, HL; Bulmahn, JC; Mahajan, SD; He, GS; Prasad, PN

Antiretroviral therapy (ART) has had a significant impact on extending life expectancy in patients with HIV. Current therapies can reduce viral load below the detection limit; however, the virus can still reside within sanctuary sites (e.g., the brain). In addition, patients with HIV experience HIV-associated neurocognitive disorders (HAND). Therefore, developing novel formulations of HIV therapeutics capable of crossing the blood-brain barrier (BBB) could have significant clinical value by reducing the need for high-dose therapeutics. This presentation focuses on using laser ablation of ART drugs (atazanavir, ritonavir) combined with a theranostic imaging agent (curcumin). Briefly, crystalized drugs were subjected to femtosecond laser ablation in water containing F127 surfactant. The resulting ultrasmall (20–25 nm) nanoparticles can cross the BBB and remain biologically active to reduce HIV replication. This unique nanofabrication method has potential for clinical translation of poorly soluble and/or poorly permeable therapeutics that suffer from BBB crossing. Research presented was supported by University of Rochester Center for AIDS Research (CFAR) grant P30AI078498 (NIH/NIAID); Ruth L. Kirschstein NRSA Training Grant 1T32GM099607; UL1TR001412(NIH/NCATS); and R01AI129649 (NIH/NIAID).

### Raman hyperspectral imaging and analysis of complex biological systems

Sharikova, A., PhD., Department of Physics, SUNY Albany

Raman hyperspectral imaging, also known as Raman micro-spectroscopy, is widely used to map chemical composition within biological samples, such as cells, organoids, and tissues. It enables non-invasive and non-destructive measurements that do not require special sample preparation, such as dye labelling or staining. Currently, only invasive cell biological assays are used to monitor the expression level and subcellular location of proteins that bind iron or are involved in ferroptosis. Our group has previously reported a Raman-based method to identify iron-bound transferrin in cell cytoplasm. We have also developed Raman spectroscopic signatures that can be used to monitor the differentiation state and health of salivary organoids derived from progenitor cells that undergo differentiation in culture on their own and in the presence of alginate hydrogel scaffolds. Raman spectroscopy offers a crucial noninvasive tool capable of assessing cell phenotype. When cell morphology and phenotype change, this process is accompanied by changes in the protein structure within cells. We have previously shown that the secondary/tertiary protein structure changes can be detected by Raman spectroscopy, even in tissue engineered constructs, before they can be seen histologically. These studies pave the way for utilizing Raman micro-spectroscopy as an early predictor of the ultimate success of an in vivo implanted construct.

### Banquet

Robert Gallo, MD, Distinguished Professor, University of South Florida, and Chair, Scientific Leadership Board of the GVN

#### Viral Immunology Past, Present and Future

Best known for the co-discovery of HIV, Dr. Robert Gallo pioneered the development of the HIV blood test, which enabled the health care community to screen for infection – leading to a more rapid diagnosis while simultaneously protecting patients receiving blood transfusions. His research led physician-scientists to develop antiretroviral therapies to improve the quality of life and longevity of those infected with the virus. In 1996, his discovery that a natural compound known as chemokines that block HIV infection halted disease progression. The community of scientists hailed this as one of that year’s most important scientific breakthroughs. This also helped others identify CCR5 as the HIV co-receptor. Before the DS epidemic, Dr. Gallo was the first to identify a human retrovirus and the only known human leukemia virus – HTLV – one of few known viruses shown to cause human cancer. In 1976, he and his colleagues discovered Interleukin-2, a growth factor now used as therapy in cancer, autoimmunity, and HIV/AIDS. In 1986, he and his group found the first new human herpes virus in more than 25 years (HHV-6), which was later shown to cause an infantile disease with links to neurodegenerative diseases.

## Symposia 10: Advances in the Informatics analyses of Multi-Omics Data to Disentangle the Complex Interactions of HIV Infection with Drugs of use/misuse

### Morphine potentiates HIV Tat-mediated neuroinflammation and neurodegeneration.

Deshetty, UM, PhD^1^, Singh, S, PhD^1^, Callen, S^1^, Periyasamy, P, PhD^1^, Buch, S, PhD^1^; ^1^Pharmacology & Experimental Neuroscience, University of Nebraska Medical Center, Omaha, NE

Over 49 million people worldwide are living with HIV (PLWH), and nearly 50% experience HIV-associated neurocognitive disorders (HAND), despite effective combined antiretroviral therapy (cART). Opioid misuse further exacerbates HAND prevalence, as the intersection of HIV and substance abuse accelerates neuropathology. Morphine has been shown to interact with HIV Tat, promoting microglial activation and upregulating HIV coreceptors, thereby enhancing neuropathogenesis. Our findings demonstrate that morphine induces synaptic alterations and exacerbates HIV Tat-induced neuroinflammation and neuronal injury. Moreover, morphine amplifies HIV Tat toxicity in human neurons and neuroblastoma cells, highlighting its detrimental impact on PLWH with opioid use disorder, where neuropathology is often more severe. In macaque studies, morphine dependence in SIV-infected macaques increased macrophage/monocyte infiltration. It enhanced viral replication in the central nervous system (CNS), suggesting that morphine facilitates monocyte transmigration, thereby worsening neuroinflammation. Additionally, morphine differentially affects SIV reservoirs, reducing the CD4+ T-cell reservoir in lymphoid tissues while expanding the microglia/macrophage reservoir in the CNS. To further elucidate these mechanisms, we will present single-cell RNA sequencing analysis of microglia from morphine-administered SIV-infected macaques. These findings provide critical insights into opioid-mediated neuroinflammation and may inform therapeutic strategies for mitigating HAND in PLWH.

### Single-cell transcriptomics reveals neuron- and glia-specific transcriptional changes in the adolescent midbrain following neonatal morphine exposure, which are ameliorated with probiotic intervention.

Tao, JT, PhD^1^, Antoine, DA, PhD^2^, Boyles, SMB, PhD^3^, Hulme, WH, PhD^3^, Roy, SR, PhD^1^; ^1^Department of Surgery, University of Miami Miller School of Medicine, Miami, FL 33136 ^2^Department of Neuroscience, University of Miami Miller School of Medicine, Miami, FL 33136^3^3John P. Hussman Institute for Human Genomics, University of Miami Miller School of Medicine, Miami, FL 33136.

Neonatal morphine is commonly administered in the Neonatal Intensive Care Unit to manage pain, but its long-term effects on neurodevelopment remain a significant concern. The midbrain is a core region that plays a central role in pain processing and opioid-mediated analgesia, yet cell-type-specific transcriptional changes following neonatal morphine exposure remain unexplored. Here, we performed single-cell RNA sequencing and studied gene expression in 107,427 midbrain single cells from adolescent mice exposed to morphine, probiotic Bifidobacterium infantis, or saline neonatally for 5 days. We found that neuronal and glial transcriptomics were broadly altered, involving thousands of differential gene expressions within neurons, astrocytes, and oligodendrocytes. Ingenuity Pathway Analysis revealed that pathways related to gene transcription, neurotransmitter signalling, and cellular immune response were significantly upregulated with neonatal morphine exposure. Interestingly, neonatal probiotic supplementation mitigated the neonatal morphine-induced alterations on the transcriptome, with pathways related to neurotransmitter signalling, and cellular immune response downregulated during adolescence. This report is the first single-cell RNA sequencing dataset from the adolescent midbrain following neonatal morphine exposure and probiotic intervention, providing unique insights in understanding brain alterations following early opioid exposure and the mechanisms underlying microbiome-targeted therapeutics.

### Exploring the biology of HIV-1 latency in the CNS as a pathway to developing a cure

Li, Q., PhD, Willa Cather Professor at the School of Biological Sciences, University of Nebraska Lincoln

Antiretroviral therapy (ART) has revolutionized HIV-1 treatment by transforming it from a fatal condition into a manageable chronic disease. However, upon ART interruption (ATI), viral replication rapidly rebounds due to the reactivation of latent reservoirs. While quiescent memory CD4 T cells represent the primary HIV reservoir outside the central nervous system (CNS), myeloid cells within the CNS present an additional challenge for achieving an HIV-1 cure. This talk will focus on the current understanding of HIV-1 latent reservoirs in the CNS at molecular and cellular levels. It also highlights our efforts to investigate CNS reservoirs using brain tissues from humans, macaques, and humanized mice, with implications for future cure strategies.

### Cannabidiol Decreases HIV/SIV Infection, Reduces the Latent Viral Reservoir, and Suppresses Inflammatory Cytokines in Rhesus Macaques and *in vitro* studies with Primary Human Cells.

Ellison AL*, Rosado-Franco JJ*, Mamun, L*, Knerler S, Daniali M, Mehta K, Williams-McLeod S, Joyner S, MacLean A, Gaskill PJ, Corley M, Ndhlovu L, Vandrey R, Weerts E, Williams, DW

Chronic, immune activation is one of the hallmarks of HIV in the modern era, despite the effectiveness of antiretroviral therapy (ART) in suppressing viral replication. Significant electronic inflammation that occurs contributes to the development of comorbid disorders that hinders the quality of life of infected people and increases mortality. Unexpectedly, ART is insufficient to restore markers of immune activation to their pre-infection levels. Thus, this is an ideal time to identify novel adjunctive agents to limit inflammation and its consequent effect on comorbid disease. We aim to characterize the impact of cannabidiol (CBD), a component of the cannabis plant, on HIV-associated chronic immune activation. We hypothesize that CBD will decrease inflammation and decrease viremia by activating the endocannabinoid system. Four juvenile rhesus macaques (n=2 male, n=2 female, aged 4 years old) were infected with 20AID50 of SIV_mac251_ for seven days, after which oral CBD was administered. I*n vitro* experiments with primary human macrophages and T cells were also performed. SIV infection promoted viremia, as determined by detecting the p27 protein in plasma, which peaked at 2128 ± 1073 pg/mL at 2-weeks post-inoculation. However, CBD significantly decreased viremia to 713 ± 440 pg/mL at the terminal timepoint. Decreased viremia coincided with a significant decrease in 15 plasma cytokines involved in the antiviral response, including Type I and II interferons, IL-1b, and TNF-a between 10–60 fold, often to levels below the limit of detection. CBD did not reverse the loss of CD4+ T-lymphocytes known to occur during acute HIV/SIV infection. However, CBD increased monocyte frequency at the terminal timepoint (10.8 ± 2.6%) relative to acute infection (6.9 ± 2.8%) and increased their markers of immune response (TLR2 and CD42a) by 2.8 and 1.4 fold, respectively. Unexpectedly, CBD did not alter endocannabinoid ligands in plasma or CSF, but did modulate 10 endocannabinoid receptors in eight tissues, suggesting a change in signaling mediates its effects. Similar trends occurred with human cells where CBD decreased HIV infection, measured by p24 antigen, and cytokines released in the supernatant. Our data indicate CBD administration as a promising adjunctive therapeutic strategy during HIV infection that can decrease viremia, plasma markers of chronic inflammation, and immune cell antiviral responses, even in the absence of ART. These findings suggest a clinical benefit of CBD that may improve quality of life in people living with HIV and decrease risk of comorbid disease.Supported by: R01DA052859, U01DA058527, and P30 AI050409

### Bill Narayan Memorial Lecture

Howard Fox, PhD, Professor, University of Nebraska Medical Center, Omaha, NE

#### Omics unleashed: revolutionizing neuroimmune pharmacology with data-driven science

From Galen to da Vinci to Cahal and now to us, understanding the brain in both health and disease has been a formidable challenge for scientists. The advent of technologies that allow us to gather vast amounts of data in quantitative, sensitive, and comprehensive ways has not only provided answers to longstanding questions but also raised new ones. We have harnessed omics, alongside computational analyses and biological insights, to explore the impact of HIV infection on the brain as well as in parallel the effects of substance abuse. By utilizing the SIV infection model in rhesus macaques, our team and collaborators have discovered new pathogenic mechanisms in these conditions relevant to human health.

Supported by the National Institute of Mental Health and the National Institute on Drug Abuse.

## Symposium 11: SNIP member symposium will be announced

### Symposium 12: Local organizing committee symposium

#### Accelerated Dysregulation of Neuroimmune Markers in chronically HIV-infected and Aged Humanized Mice

Chen Zhang*, Rashmi Rikhi*, Larisa Y Poluektova*, Santhi Gorantla*, Howard E Gendelman, Prasanta K. Dash, Department of Pharmacology and Experimental Neuroscience, University of Nebraska Medical Center, Omaha, NE.

Aging is associated with persistent levels of inflammation, which is also a hallmark of HIV infection. Despite practical cART, people living with HIV (PLWH) are at high risk for age-related diseases and accelerated aging. However, the underlying mechanisms contributing to these risks, like chronic systemic inflammation, senescence, and mitochondrial and CNS dysfunction, have not been completely defined. We employed a CD34-NSG humanized mouse model, which mimics a human immune system and can sustain chronic HIV infection, to study aging-related pathways during HIV infection. The mice were observed up to 15 months post-humanization and 10 months post-infection for immune and viral level analysis. We also evaluated aging-associated markers in the brains of HIV-infected and uninfected mice using qPCR, transcriptomics, and immunofluorescence assays. CD4+ T cell decline was observed to be associated with viral level and age. Transcriptomics revealed an upregulation of COL1A, CD163, and CXCL16 and a downregulation of LMNA and CLU genes. Ingenuity pathway analysis affirmed links to immune activation, cellular senescence, neuroinflammation, mitochondrial dysfunction, and pyroptosis signaling. Humanized mice are identified as a valuable model for studying chronic HIV infection and aging. Neuronal signaling and immune senescence pathways are found to be associated with HIV infection and aging. Exploring the underlying molecular mechanisms in humanized mice will help identify suitable aging biomarkers of the CNS and help design therapies for aging-related complications in PLWH.

### Neural Mechanisms underlying Cardiac Dysfunction and Cardiac Remodeling in Chronic Heart Failure

Han-Jun Wang M.D. Department of Anesthesiology, University of Nebraska Medical Center, NE 68198, USA

Chronic heart failure (CHF) is a serious and debilitating condition with poor survival rates and an increasing level of prevalence in the aging population. The exaggerated sympatho-excitation that is a hallmark of CHF is a critical factor in the development and progression of the CHF state. The enhanced cardiac spinal afferent reflex (CSAR) critically contributes to CHF’s exaggerated global sympathetic tone. Our previous studies found that chronic ablation of CSAR by epicardial application of a selective afferent neurotoxin, resiniferatoxin (RTX), improves cardiac diastolic dysfunction, cardiac remodeling, venous congestion and renal dysfunction, ultimately enhancing long-term survival in CHF rats. Our recent unpublished findings further suggest that neural inflammation is a key driver of chronic cardiac afferent sensitization. Using a coronary ligation-induced myocardial infarction (MI) rat model, we observed a marked increase in Iba1-positive macrophages and the pro-inflammatory macrophage marker Interferon regulatory factor 8 (IRF8) in thoracic (T1-T4) dorsal root ganglia (DRGs) starting at four weeks post-MI and persisting for at least four weeks. The anti-inflammatory drug minocycline (Mino) largely prevented these inflammatory changes and significantly depleted by clodronate liposomes. Both treatments attenuated the exaggerated CSAR in CHF rats. These findings highlight neural inflammation as a promising therapeutic target for mitigating cardiac spinal afferent sensitization and improving cardiac function in CHF.

### Molecular Signatures in Rebound Viruses from Antiretroviral Drug and CRISPR Treated HIV-1-Infected Humanized Mice

Zhang C, PhD^1^, Li HT, MS^2^, Poluektova LY, MD, PhD^1^, Gendelman HE, MD^1^, Dash PK, PhD^1 1^Department of Pharmacology and Experimental Neuroscience, University of Nebraska Medical Center, Omaha, NE. ^2^School of Biological Sciences, University of Nebraska-Lincoln, Lincoln, NE.

HIV-1 elimination from a subset of virus-infected humanized mice (hu-mice) was reported following sequential dual-treated long-acting (LA) antiretroviral (ART) and CRISPR-Cas9 therapies. However, viral rebound is observed in >50% of the dual-treated animals. The molecular signatures of the rebound virus, recovered from plasma, are now determined. The LA-ART treatment comprised dolutegravir, lamivudine, abacavir, and rilpivirine combinations and HIV-1 excision treatment was CRISPR-Cas9 targeting the HIV-1-LTR-gag. One-step reverse transcriptase polymerase chain reaction, which avoids spontaneous preparatory mutations, is performed on plasma-derived RNA. Sanger and Next-Generation Sequencing are employed to analyze the HIV-1 gag, pol, and env genes. HIV-1env showed the most divergence. LA-ART, with or without CRISPR, is responsible for the new mutations. The primary and accessory mutations are detected by deep sequencing. Viral evolution reflects changes in the virus as reported by ART-treated and HIV-1-infected patients. No major CRISPR-specific mutations are observed. The molecular viral signatures demonstrate an accelerated HIV-1 drug resistance escape from ART rather than from the generation of CRISPR mutants, defining viral rebound in the dual-treated human mice. The data underscores the limited role of CRISPR excision in generating these rebound HIV-1 mutants from dual-treated hu-mice.

### Aging and Reservoirs in People with HIV (PWH)

Arpan Acharya PhD^1^, Debapriya Sutar^1^, Mahesh Mohan^2^, Varan Govind^3^, Francois Villinger^4^, Suresh Pallikkuth^5^, Siddappa N. Byrareddy^1^ 1Department of Pharmacology and Experimental Neuroscience, University of Nebraska Medical Center, Omaha, NE, ^2^Southwest National Primate Research Center, Texas Biomedical Research Institute, San Antonio, Texas, United States ^3^Department of Radiology, University of Miami Miller School of Medicine, Miami, Florida, ^4^Department of Biology, University of Louisiana at Lafayette, Lafayette, Louisiana, ^5^Department of Microbiology and Immunology, University of Miami Miller School of Medicine, Miami, Florida

The hallmark of chronological aging is a progressive dysfunction of innate and adaptive immune responses associated with clonal expansion of memory T cells with simultaneous loss of naïve T cells, resulting in a naïve-memory imbalance. With the advancement of combined antiretroviral therapy (ART), the life expectancy of people living with HIV (PLWH) becomes comparable with the general population. As the proportion of older PWH keeps increasing with time, it becomes critical to understand the impact of age-related immune dysfunction on HIV reservoirs. We hypothesize that accelerated clonal expansion of memory T cells with aging will expand the HIV reservoirs in aged PWH. In a preliminary study, we estimated the frequency of intact and total HIV proviral DNA from young and old PLWH with suppressed plasma viremia. Next, we quantified the intact and total proviruses from SIV-infected ART-suppressed young and aged rhesus macaques. We observed a significantly higher level of intact and total proviral DNA in older compared to younger PWH. The frequency of intact provirus is positively correlated with activated CD4 and CD8 T cells. Similarly, in rhesus macaques, we found significantly higher levels of intact and total proviruses in aged compared to young macaques. Our results suggest an expansion of HIV/SIV reservoirs in circulation in older people/macaques compared to their younger counterparts.

## Early Career Investigators Travel Award (ECITA) Abstracts

1. Humanized glia mice exhibit HIV-induced behavioral, neurobiological, and metabolic changes similar to clinical disease. Fernandes, A, BS^1^, Makarov, E, MS^1^, Thiele, M, BS^1^, Liu, Y, PhD^1^, Samuelson, M, PhD^1^, Gorantla, S, PhD^1^; ^1^Department of Pharmacology & Experimental Neuroscience, University of Nebraska Medical Center, Omaha, NE 68198.
  Effective antiretroviral therapy (ART) reduced the severity but not the frequency of HIV-associated neurocognitive disorders (HAND), which affect cognitive, mood, and motor functions in people living with HIV. We developed a novel humanized glial mouse model reconstituted with the human immune system and glia to study HAND pathology and behavior during ART-suppressed HIV infection, mimicking human disease. Mice were infected with HIV-1ADA and treated with ART. Magnetic resonance spectroscopy (MRS) and diffusion tensor imaging (DTI) were used to assess brain metabolic and structural changes, while neuropathology was analyzed by immunohistology. Mood disorders (sucrose anhedonia test) and cognitive deficits (novel object recognition test) were also examined. Changes in fractional anisotropy (FA) and apparent diffusion coefficient (ADC) in the whisker barrel (FA p=0.02; ADC p=0.02), frontal cortex (FA p<0.001), and corpus callosum (ADC p=0.02), indicating altered white matter integrity in HIV-infected and ART mice. Immunohistology showed significant demyelination in corpus callosum and cerebellum (p<0.05). MRS identified increased phosphocholine and choline (p<0.0001), and glutamine (p=0.02) in hippocampus, along with decreased glucose and altered myoinositol (p<0.005) in cortex. Behavioral analysis confirmed decreased sucrose preference (p=0.004), reduced memory recognition index (p<0.05), in infected mice. Humanized glial mouse mirrors HIV brain disease in humans, displaying significant neuropathological, behavioral, and brain structural changes that persist despite ART. Supported y NIH/NIDA 1R01DA054535-01; NIH/NIMH 1 R01 MH128009-01; 5T32NS105594.
2. Animal model to decipher mechanisms responsible for sudden cardiac death in HIV-1 infection. Namvaran, A, PhD^1^, Garcia, J, BS^1^, Hackfort, BT, PhD^2^, Dash, P, PhD^1^, Edagwa, BJ, PhD^1^, Gorantla, S, PhD^1^, Bidasee, KR, PhD^1^; ^1^Department of Pharmacology and Experimental Neuroscience, University of Nebraska Medical Center, Omaha, NE 68198-5800 ^2^Cellular and Integrative Physiology, University of Nebraska Medical Center, Omaha, NE 68198-5800.
  People living with HIV-1 infection (PLWH) are 2–4 times more likely to succumb to sudden cardiac death (SCD) compared to the general population. Studies attribute this to a fatal ventricular arrhythmia arising from abnormalities in the electrocardiogram (ECG) due to myocardial ischemia, fibrosis, cardiac diastolic dysfunction (DD), and occult drug overdose. Here we investigated if our HIV-1 infected Hu-mice that develop myocardial ischemia, fibrosis, and DD also create changes in ECG. Hu-mice were infected with 5 X 106 viral particles of HIV-1. After 4 weeks, animals were divided into two groups and treated with DTG/TDF/FTC or no treatment for 8 weeks. Uninfected Hu-mice served as controls. ECG and DD were measured every 4 weeks and normalized to heart rate. Before euthanasia, animals were injected with BSA-FITC to assess microvessel perfusion/ischemia. Excised hearts were stained for fibrosis. After two months of infection, P-R interval and QRS complex decreased by 9% and 18 % (p < 0.05), while QT interval, ST segment, and MVET (left ventricular filling time) increased by 24%, 35%, and 23% (p < 0.05) over controls. Perfused microvessels density was reduced by 39% and fibrosis increased by 280%. Treatment with DTG/TDF/FTC minimally attenuated ECG and DD changes, ischemia, and fibrosis. These data are the first to show abnormalities in ECG pattern and MVET in anesthetized HIV-1 infected Hu-mice with and without ART. They also suggest that HIV-1-infected Hu-mice is an appropriate model to delineate mechanisms underlying SCD in PLWH. Supported by Funding: 1R01HL164306, R21NS139920, and R56HL151602-01A1.
3. Methamphetamine-mediated astrocytic pyroptosis and neuroinflammation involves miR-152-NLRP6 inflammasome signaling axis. Oladapo, A, PhD^1^, Kannan, M, PhD^1^, Deshetty, U, PhD^1^, Singh, S, PhD^1^, Buch, S, PhD^1^, Periyasamy, P, PhD^1^; ^1^Pharmacology and Experimental Neuroscience, University of Nebraska Medical Center, OMAHA, NE 68198.
  Methamphetamine is a widely abused drug associated with significant neuroinflammation and neurodegeneration, primarily through the activation of glial cells and neurons in the central nervous system. This study investigates the role of the astrocyte-specific NOD-like receptor family pyrin domain-containing protein 6 (NLRP6) inflammasome in methamphetamine-induced astrocytic pyroptosis and neuroinflammation. Our findings demonstrate that methamphetamine exposure induces NLRP6-dependent pyroptosis, astrocyte activation, and the release of proinflammatory cytokines in mouse primary astrocytes. Gene silencing of NLRP6 significantly reduces methamphetamine-induced pyroptosis and proinflammatory cytokine release. We also identified miR-152 as a critical upstream regulator of NLRP6, which is downregulated in methamphetamine-exposed astrocytes. Overexpression of miR-152 decreases NLRP6 expression, mitigating methamphetamine-induced pyroptosis and inflammation. In vivo and ex vivo studies in methamphetamine-exposed mice confirmed these findings and demonstrated that methamphetamine induces anxiety-like behavior, cognitive impairment, and depression-like behavior, further linking astrocyte-specific NLRP6 signaling to methamphetamine-induced neuroinflammation. This study highlights the potential of targeting the NLRP6 inflammasome in astrocytes as a therapeutic approach to alleviate methamphetamine-induced central nervous system pathology. Further research is warranted to explore clinical applications and identify therapeutic targets for methamphetamine-related neurological disorders. Supported by National Institutes of Health – National Institute on Drug Abuse: DA052266 and DA060753.
4. SRI-47056 a pyrimidine structure-based compound allosterically modulates monoaminergic transporters, attenuating the dysregulation of dopamine and serotonin uptake induced by HIV-1 transactivator of transcription (TAT). Jimenez, AC, PhD^1^, Moukha-Chafiq, O, PhD^2^, Nguyen, T, PhD^2^, Subramaniam, A, PhD^2^, Augelli-Szafran, C, PhD^2^, Chang-Guo, Z, PhD^3^, Zhu, J, MD, PhD^1^; ^1^Department of Drug Discovery, Collage of Pharmacy, University of South Carolina, Columbia, SC 29208 ^2^Department of Chemistry, Southern Research Institute, Birmingham, AL 35205 ^3^Department of Pharmaceutical Sciences, College of Pharmacy, University of Kentucky, Lexington, KY 40536
  We have demonstrated the allosteric interaction of HIV-Tat protein with the dopamine transporter (DAT), norepinephrine (NET), and serotonin transporter (SERT) as the main mechanism to disrupt the monoaminergic transmission in HIV infection, which a novel allosteric modulator, SRI-32743, can attenuate. This study determined the pharmacological profile of SRI-47056, a novel analog of SRI-32743, with improved solubility and metabolic stability and stabilized dopamine and serotonin levels in iTat-tg mice. SRI-47056 displayed IC50 values to inhibit [3H]dopamine uptake via hDAT of 0.96 ± 0.05 µM; hNET 0.24 ± 0.03 µM and, hSERT 3.79 ± 1.44 µM, showing a partial inhibition of dopamine uptake (Emax value 32.33 to 55.58 %). SRI-47056 partially inhibited [3H]5-HT uptake (IC50= 5.40 ± 1.09, Emax= 65.85 ± 13.17) and [3H]norepinephrine uptake (IC50= 0.06 ± 0.1, Emax= 48 ± 12.7). SRI-47056 diminished the cocaine-mediated dissociation of [3H]WIN35,428 binding in hDAT (K-1=0.133 ± 0.036 min-1 and K-1 =0.086 ± 0.043 min-1, respectively) and [3H]nisoxetine binding in hNET (K-1=0.087 ± 0.027 min-1 and K-1 =0.031 ± 0.003 min-1, respectively) relative to cocaine alone (0.309 ± 0.039 min-1, 0.143 ± 0.011, respectively). SRI-47056 attenuated Tat (8.7 or 17.5 nM)-induced decrease in [3H]DA uptake via hDAT and hNET and [3H]5-HT uptake. These findings suggest that attenuating Tat binding to the monoamine transporters through novel allosteric modulators with minimal effects on physiological changes in monoaminergic transmission preserve neurocognitive function in HIV infected individuals. Supported by NIH grants DA035714, DA047924, and DA057866.
5. Creation of an ultra-long-acting prodrug of buprenorphine for opioid dependence. Sultana, Ashrafi, MS^1^, Sillman, Brady, PhD^2^, Deodhar, Suyash, PhD^2^, Le, Nam Thai Hoang, BS^2^, Nayan, Mohammad Ullah, PhD^2^, Gendelman, Howard E., MD, PhD^2^, Edagwa, Benson J., PhD^2^; ^1^Medical Sciences Interdepartmental Area – Patient Oriented Research, University of Nebraska Medical Center, Omaha, NE 68105-1169 ^2^Pharmacology and Experimental Neuroscience, University of Nebraska Medical Center, Omaha, NE 68105-1169.
  Opioid Use Disorder (OUD) remains a critical public health crisis in the United States, necessitating innovative treatment options. Buprenorphine (BUP), a partial μ-opioid receptor (MOR) agonist, has demonstrated efficacy in reducing mortality and preventing overdose compared to methadone and naltrexone. BUP is available in daily oral/sublingual dosage forms and subcutaneous monthly injectables. However, the bitter taste and diversion-related concerns associated with the oral dosage forms, patient acceptance, and tolerability of frequent injections that utilize organic solvents are significant limitations. Several studies have demonstrated that patients prefer longer-acting dosage forms due to the reduced medication burden associated with the sustenance of continuous drug levels. With the patient’s needs in mind, we developed organic solvent-free ultra-long-acting (ULA) BUP prodrug formulations. Preclinical studies in Sprague Dawley rats demonstrated that a lead prodrug nanosuspension, NM6BUP, sustains therapeutic BUP concentrations in plasma and brain over 182 days following a single intramuscular injection. NM6BUP was well tolerated without adverse effects such as weight loss. The extended pharmacokinetic and safety profiles of the prodrug formulation and the existing BUP efficacy data support further development of NM6BUP as a ULA formulation for treating OUD. Supported by The University of Nebraska Foundation, which includes donations from Dr. Carol Swarts, M.D.
6. Altered NLRP3 inflammasome gene expression in monocyte-derived macrophages linked to cannabis use in people living with HIV. Avalos, BA, PhD^1^, Walter, KC, BS^1^, Ford, MK, BS^1^, Laird, AE, BS^1^, Boustani, A, MD^1^, Spencer, M, BS^1^, Shu, L, BS^1^, Chaillon, A, PhD^3^, Crescini, M, BS^1^, Cookson, D, PhD^1^, Ellis, RJ, PhD^2^, Letendre, SL, PhD^3^, Iudicello, J, PhD^1^, Fields, JA, PhD^1^; ^1^Department of Psychiatry, UC San Diego, La Jolla, CA 92093 ^2^Department of Neurosciences, UC San Diego, La Jolla, CA 92093 ^3^Department of Medicine, UC San Diego, La Jolla, CA
  Background: Human immunodeficiency virus (HIV) infection is associated with chronic inflammation and cognitive dysfunction, even in people living with HIV (PWH) on antiretroviral therapy (ART). The NLRP3 inflammasome drives inflammation by promoting the secretion of interleukins (IL)-1β and IL-18. Cannabis use and cannabinoids, such as cannabidiol (CBD), may modulate inflammation and offer therapeutic benefits in chronic inflammatory conditions. Methods: mRNA levels of NLRP3, IL1β, and IL18 were measured in monocyte-derived macrophages (MDMs) isolated from PWH with varying cannabis use patterns (i.e. daily, moderate, naive/non-user). Additionally, donor-derived MDMs were treated with CBD, IL1β, or a combination of CBD + IL1β for 24 hours to examine their effects on NLRP3-related gene expression. Clinical data were analyzed for potential correlations. Results: HIV+ daily cannabis users exhibited higher NLRP3-related gene expression compared to non-users and moderate users. CBD treatment reduced NLRP3 mRNA expression in MDMs, but the combination of CBD + IL1β led to significant increases in IL1β and IL18 expression, indicating complex interactions with inflammatory pathways. Conclusions: CBD, as part of cannabis use, may reduce NLRP3 activation in PWH, potentially offering therapeutic benefits for chronic inflammation. However, its variable effects on cytokine expression highlight the need for further research to clarify its role in modulating the NLRP3 inflammasome and broader inflammation in PWH.
7. Cannabidiol’s vascular protective effects on the Blood-Brain Barrier counteracts adverse outcomes from hiv virotoxins and inflammatory insult. Birru, BB, PhD^1^, Andrews, AMA, PhD^1^, Ramirez, SHR, PhD^1^; ^1^Department of Pathology, Immunology and Laboratory Medicine, University of Florida, Gainesville, FL
  Despite the benefits of anti-retroviral therapy for controlling HIV infection, neuroinflammation and HIV-associated vasculopathy persist. In fact, accelerated atherosclerosis, vasculitis, small vessel disease, and disruption of the blood-brain barrier (BBB) contribute to various neurological disorders in people living with HIV (PWH). Individuals affected by medical ailments (including PWH) use cannabis products for its anti-inflammatory, pain management, and appetite promoting effects. Here, we explore whether cannabidiol (CBD) offers brain endothelial protective effects in the context of inflammation and HIV virotoxins. First, gene expression analysis for CNR1 and CNR2 (cannabinoid receptors) was performed on human brain microvascular endothelial cells (HBMVECs) from various donors. Results show mild expression of the cannabinoid receptors but with some degree of donor variability. Assays for testing barrier integrity were performed in the presence of HIV viral proteins (Tat, Nef, Gp120) and inflammatory cytokines with or without CBD. Functional and structural outcomes for BBB permeability revealed a significant reversal of BBB breach (induced by HIV virotoxins and cytokines) by CBD. Our data suggest that CBD, likely via CB1 and CB2, on HBMVECs induce molecular events that strengthen barrier tightness, which are long-lasting and protective against HIV virotoxins. Future studies will explore whether these effects extend toward enhancing other aspects of cerebrovascular health. Supported by R01DA052850.
8. Persistent opioid-associated dysregulation of the innate immune system in people living with HIV. Bever, B, MS^1^, Park, B, PhD^1^, Cook, R, PhD^1^, Korthuis, T, MD^1^, Underwood, M, BS^1^, Carbone, L, PhD^1^, Davis, B, BS^1^, Pereira Ribeiro, S, PhD^2^, Lancioni, C, MD^1^; ^1^Pediatrics, Oregon Health and Science University, Portland, OR 97239 ^2^Pathology and Laboratory Medicine, Emory University, Atlanta, GA.
  The impact of opioids on immunity is poorly understood among people living with HIV (PWH). We hypothesized that PWH and opioid-use-disorder (OUD+) would have sustained alterations in innate immune responses despite optimization of viral load (VL) with antiviral therapy (ART). PBMCs and plasma were collected from PWH/OUD+ undergoing ART optimization over 6-months (n=59), and PWH/OUD- with undetectable VL (n=44). Plasma cytokines, sCD14, sCD163 and monocyte responses to LPS were assessed. We performed cross-sectional comparisons between PWH/OUD±, and a longitudinal analysis among PWH/OUD+. sCD14 and sCD163 were increased in PWH/OUD+ at all timepoints (p<0.001, VL adjusted), without significant changes over time. PWH/OUD+ had diminished levels of plasma TGF-β1, TGF-β2, but increased Fraktalkine and ITAC at all timepoints (p<0.01, VL adjusted), without significant changes over time. Monocytes from PWH/OUD+ produced less IL-1β, IL-10, TNF-α, and MIP-3α in response to LPS at all timepoints (p<0.05). Gene set enrichment analysis of bulk RNAseq of monocytes from PWH/OUD+ showed alterations in pathways associated with glycolysis, mTORC1, OXPHOS, and ROS. OUD is associated with elevated biomarkers of innate immune activation, altered plasma cytokines and monocyte functional responses that do not normalize within 6-months of ART. Identifying cellular mechanisms driving opioid-associated monocyte dysfunction is critical due to the potential for monocytes to drive systemic and neuro-inflammatory responses among PWH, and to serve as a HIV reservoir within both the CNS and periphery. Supported by RO1DA046229.
9. Role of organellar stress responses in bace-1 inhibitor drugs-induced neurotoxicity. Quansah, Darius N.K, MS^1^, Geiger, Jonathan, PhD^1^; ^1^Department of Biomedical Science, University of North Dakota, Grand Forks, ND 58203.
  Alzheimer’s disease (AD) is pathologically characterized by extracellular plaques composed of amyloid beta (Aβ) proteins that are produced by amyloidogenic cleavage of Aβ-precursor protein (AβPP), a process controlled by the rate-limiting beta-site AβPP cleaving enzyme (BACE-1). BACE-1 inhibitor drugs significantly reduce Aβ levels but have neurotoxic effects by unclear mechanisms. BACE-1 resides in endolysosomes that contain sufficiently large stores to ferrous iron (Fe2+) to cause insult-induced increases in intracellular Fe2+, reactive oxygen species (ROS) and cell death. We tested here the extent to which BACE-1 inhibitors cause neurotoxicity by perturbing endolysosome stores of Fe2+. Using SH-SY5Y human neuroblastoma cells, we determined the effects of clinically relevant concentrations of three BACE-1 inhibitors (elenbecestat, lanabecestat and verubecestat) and two non-BACE-1 anti-AD drugs (memantine and galanthamine) on endolysosome and mitochondrial stress responses. The BACE-1 inhibitor drugs but not the non-BACE-1 anti-AD drugs de-acidified endolysosomes, decreased endolysosome Fe2+ levels, increased cytosolic Fe2+ and ROS levels, increased mitochondrial Fe2+ and ROS levels, and caused mitochondrial membrane depolarization. The endocytosed endolysosome Fe2+ chelator deferoxamine blocked the neurotoxic effects of BACE-1 inhibitor drugs. These findings emphasize the importance of endolysosome Fe2+ in developing new therapeutics against AD. Supported by Research Funding: 2R01DA032444, P20GM139759, RO1NS065957.
10. Misfolded protein aggregates reactivate HIV CNS reservoirs. Sutar, Debapriya, MS^1^, Acharya, Arpan, PhD^1^, Byrareddy, Siddappa, PhD^1^; ^1^Department of Pharmacology and Experimental Neuroscience, University of Nebraska Medical Center, Omaha, NE 68198.
  With the advancement of combination antiretroviral therapy, people with HIV (PWH) live longer. However, due to the persistence of latent reservoirs curing of HIV remains elusive. Chronological aging is associated with the accumulation of amyloid fibrils in the CNS, which plays a significant role in the development of Alzheimer’s and Alzheimer’s disease-related dementias. However, the impact of the amyloid fibril deposition on reactivation of CNS reservoirs of older PWH remain unknown. In the present study we exposed a latently infected microglia cell line HC69.5 to Aβ1-42, α-Synuclein (αSn), and Tau preformed fibrils for 24 hours, and flow cytometry was performed to detect the GFP expression as a marker of HIV reactivation. We observed a significant increase in GFP-positive cells: 18.7% & 21.9% for Aβ1-42 (500nM, 1000nM), 27.0% & 29.0% for αSn PFF (50nM, 100nM), and 21.7% & 19.2% for Tau PFF (50nM, 100nM) compared to untreated controls. The IBA1 expression also significantly increased in Aβ1-42, α-Synuclein (αSn), and Tau treated cells, indicating microglial activation. Furthermore, when neurons were treated with the culture supernatants from different PFFs treated H69.5 cells, a significant increase in neuronal toxicity observed. Overall, these data indicates that, when latently infected microglia encounter with fibrillar Aβ, αSn, and tau, it gets activated and resume the viral transcription. Moreover, the inflammatory mediators released from these cells contributes to neuronal death. These findings underscore the potential of amyloid fibrils in modulating HIV latency.
11. Fak signaling and cell migration deficits in neural progenitor cells driven by MIR-21A-5p in methamphetamine and HIV co-treatment. Levitis, DL, BS^1^, Park, MS, PhD^1^, Toborek, M, MD, PhD^1^, Si, J, BS^1^, Herrera, A, BS^1^; ^1^Department of Biochemistry & Molecular Biology, University of Miami Miller School of Medicine, Miami, FL 33136.
  Methamphetamine (METH) exacerbates HIV-induced deficits in neuroblast migration, yet the mechanisms remain unclear. In the subventricular zone (SVZ), neuroblasts migrate to the olfactory bulb (OB) along the rostral migratory stream (RMS) within a tightly regulated neurovascular microenvironment. This study explores how METH and HIV disrupt this microenvironment and impair neuroblast migration. C57BL/6J mice were exposed to chronic METH and/or infected with a chimeric HIV-NDK (EcoHIV). Neural progenitor cells (NPCs) were isolated from the SVZ, and miRNA sequencing identified 13 miRNAs with significantly altered expression (false discovery rate (FDR) < 0.1). Among these, miR-322-5p (human miR-424) was elevated, while miR-21a-5p was reduced in the METH and HIV co-treated group. Reducing miR-21a-5p in ReNcells, a human progenitor cell line, impaired CXCL12/SDF-1-induced transmigration, similar to the effects observed in METH and HIV co-exposed ReNcells in vitro. SDF-1-induced FAK phosphorylation, critical for cell adhesion, was diminished in METH and HIV co-treated cells, while overexpressing miR-21a-5p partially rescued this impairment. These findings highlight the critical role of miR-21a-5p in regulating FAK-related cell adhesion, a process essential for NPC migration in the SVZ. METH exposure and HIV infection disrupt this mechanism, shedding light on how these conditions impair neuroblast migration and contribute to neurovascular deficits. Supported by NIH: DA060085, DA050528, DA044579, DA059849, HL126559, MH128022, and MH072567. Support from CFAR by NIH grant: P30AI073961.
12. Modeling HIV associated neuropathology in a novel microglia containing brain organoids. Domene Rubio, A, PhD^1^, Yelamanchili, S, PhD^1^, Moore, D, MS^1^; ^1^Department of Anesthesiology, University of Nebraska Medical Center, Omaha, NE 68198.
  Modeling HIV infection in brain organoids represents a promising approach to studying the impact of virus on the central nervous system (CNS). Cerebral brain organoids are 3D cultures derived from human induced pluripotentant stem cells that mimic the complexity of the brain and offer an excellent in vitro platform for investigating HIV neuroinvasion and neurocognitive disorders associated with chronic infection. This model enables us to explore the dynamics of HIV entry, replication, and the resultant neuroinflammation within a more physiologically relevant context compared to traditional cell cultures or animal models. By utilizing brain organoids, scientists can uncover key mechanisms underlying the virus’s effects on neuronal function, identify potential therapeutic targets, and assess the efficacy of antiretroviral and neuroprotective drugs. Additionally, these models provide insights into how HIV may contribute to cognitive decline and other neurological complications in people living with HIV, thus advancing our understanding of the intersection between virology and neurobiology. Here, we have established a novel organoid model that recapitulates the immunotypic human brain mimicking the Trojan horse model thereby facilitating HIV entry. Our results reveal establishing a chronic infection resulting in neuroinflammation and synaptic injury as seen with HAND.
13. Persistent HIV and associated pathology in the spinal cord of art-treated HIV-infected humanized glial mice. Etafo, EE, MS^1^, Amanda Fernandes, AF, BS^2^, Ed Makarov, EM, MS^2^, Matthew Thiele, MT, BS^2^, Divya Prakash Gnanadhas, DG, PhD^2^, Debashis Datta, DD, PhD^2^, Gorantla Santhi, SG, PhD^2^; ^1^Pharmaceutical Science Department, University of Nebraska Medical Center, Omaha, NE 68198^2^Pharmacology & Experimental Neuroscience, University of Nebraska Medical Center, Omaha, NE 68198.
  Central nervous system (CNS) reservoirs and pathology are studied mainly in the brain due to HIV-associated neurocognitive disorders (HAND), however, the spinal cord (SC) is also affected, causing motor, coordination, and sensory deficits. HIV infection of the SC can lead to neurological and motor deficits, despite effective viral suppression in the blood by ART. This condition is associated with HIV-associated myelopathy (HIVAM) and other neuroinflammatory processes in SC. We utilized a recently developed humanized glial mouse model with human microglial cell reconstitution to examine HIV-related spinal cord reservoirs and pathology. This model enables HIV infection in the CNS, allowing for the study of viral reservoirs and associated pathology. DdPCR and the neuroinflammation and pathology detected the viral RNA and DNA were analyzed by immunohistology using antibodies to GFAP, iba-1, synaptophysin, myelin associated MAG and MOG. This is the first model to demonstrate of human microglial reconstitution and productive HIV infection in mouse SC. HIV-infected SC had 105 to 106 HIV RNA copies per mg total RNA. Low levels, but detectable HIV RNA (101 to 102 copies) were found in ART mice SC. HIV infection resulted in myelin loss in the anterior column white matter, glial activation in the grey matter and demyelination in different regions of SC that may result in motor and coordination impairments seen in people living with HIV. The new mouse model can be used to study the mechanisms underlying the pathology, discover therapeutic targets and for therapeutic testing. Supported by NIH/NIDA 1R01DA054535-01 NIH/NIMH 1 R01 MH128009-01.
14. Investigating the impact of HIV-1 TAT protein on serotonin transporter function: mutation on human serotonin transporter at Asp316 is critical for serotonin transport. Gao, FG, MD, PhD^1^, Zhu, JZ, MD, PhD^1^, Jimenez Torres, ACJT, PhD^1^, Zhan, CZ, PhD^1^, DeLaney, JD, BS^1^; ^1^Department of Drug Discovery and Biomedical Sciences, College of Pharmacy, University of South Carolina, Columbia, SC 29208.
  HIV-1 Tat protein has a great impact on the development of HIV-1 associated depression through disrupting serotonin (5-HT) transmission. Through computational modeling hSERT-Tat interactions, we predicted Asp319, Glu322, and Asp569 as key residues for Tat binding. Among these mutants (N316A, E322A, N569A), we identified that mutation of hSERT at Asp316 (N316A) significantly alters basal hSERT-mediated 5-HT by changing potencies of 5-HT and SERT inhibitor (cocaine). This study determined the effects of double and triple mutations of the predicted hSERT residues on basal and Tat-induced inhibition of 5-HT transport. Compared to WT hSERT, the IC50 values of cocaine inhibiting [3H]5-HT uptake were decreased in N316A/N569A and E316A/N569A and unchanged in E322A/N569A. We also determined the IC50 values for cocaine inhibiting [3H]Citalopram (a selective SERT inhibitor) binding, which were increased in N316A/N569A and E316A/N569A but unchanged in E322A/N569A relative to WT hSERT. In addition, we found that N316A/E322A/N569A did not alter IC50 values of cocaine inhibiting 5-HT uptake but increased IC50 values of cocaine inhibiting [3H]Citalopram binding compared to WT hSERT. N316A/E322A/N569A also significantly decreased the Vmax and Km values of [3H]5-HT uptake relative to WT hSERT. Ongoing project is subjected to determine whether Tat-induced inhibition of 5-HT uptake is attenuated in these mutants. These results provide mechanistic insights into developing allosteric modulators as a therapeutic strategy for attenuating Tat-induced dysregulation of serotonin transmission. Supported by This work was supported by NIH grants DA035714, DA047924 and DA057866.
15. Drug use increase the viral reservoir pool in the human brain. Gutierrez, H, MS^1^; ^1^Department of Neurobiology, University of Texas Medical Branch, Galveston, TX 77550.
  Drug use is a significant co-morbidity of HIV associated neurocognitive disorders. Our data demonstrates that drugs of abuse potentiate the presence of viral reservoirs as well as their radius of damage in people living with HIV (PLWH). HIV has become a chronic disease, and treatment is still not curative. The main obstacle to viral eradication is the generation of viral reservoirs that perpetuate the virus in infected individuals. No quantification of viral reservoirs in tissues such as the CNS has been performed in the current antiretroviral therapy (ART) era. Data using large brain tissue sections from HIV-infected individuals under effective ART indicate that a small population of myeloid cells, such as microglia/macrophages, and a smaller population of astrocytes contain integrated HIV-DNA. Half of the cells containing HIV-integrated DNA expressed HIV mRNA, and few expressed viral proteins under ART. Viral proteins diffuse or are released into neighboring cells that lack HIV-integrated DNA, supporting a bystander mechanism of HIV-CNS damage. These mechanisms are enhanced by drug use, but the mechanism is still unknown. Here, we quantified the concentration of the virus in the brain and lymph to determine the contribution of drug use to viral reservoir stability in a large population sample. Half-life, extrapolations to reach an undetectable amount of virus in the body, as well as patient leukocyte levels are also included. This data will be useful in understanding the impact drugs of abuse have on viral reservoir stability in PLWH, leading to increased HANDS symptoms. Supported by The National Institute of Mental Health grant, MH128082/MH134761, the NINDS, NNS105584, and UTMB internal Texas funding to E.A.E.
16. Effect of chronic HIV infection on brain-derived mitovesicles. Hamilton, LJ, MS ^1^, Bausch, M ^2^, Schaal, V, BS ^1^, Gowen, A, PhD ^3^, Ware, J, MS ^1^, Pendyala, G, PhD ^1^, Yelamanchili, SV, PhD ^1^; ^1^Department of Anesthesiology, University of Nebraska Medical Center, Omaha, NE 68198 ^2^Department of Biological Sciences, University of Notre Dame, Notre Dame, IN 46556 ^3^USA Division, Sanofi, Bridgewater, NJ.
  The advent of combined antiretroviral therapy and accompanying rise in the number of older people living with HIV has precipitated a rise in prevalence of a set of mild forms of dementia, collectively called HIV-associated neurocognitive disorders (HAND). Mitochondrial dysfunction has been described in several animal models of HAND. In people diagnosed with HAND, impaired mitochondrial fission and distribution have also been reported. Recently, we and others have shown the role of extracellular vesicles (EVs) in exacerbation of HIV neuropathology. A novel class of EVs from mitochondria, namely, “mitovesicles,” were recently shown to play important roles in inflammation and neurodegeneration. Here, we investigated the effect of chronic HIV infection on mitovesicle composition. Mitovesicles were isolated from wild type (WT) and HIV-transgenic (Tg) rat brains by Optiprep density gradient ultracentrifugation techniques. Isolated mitovesicles were characterized by TEM, western blotting, and high-throughput proteomics. Results revealed significant alterations in mitochondrial proteins. Most of these were part of the mitochondrial membrane and inner mitochondrial membrane protein complexes. Functional Seahorse analysis showed that mitovesicles isolated from HIV-Tg animals increased basal respiration and ATP production in B35 neuroblastoma cells. In summary, our data reveal a significant alteration in brain mitovesicle proteins isolated from HIV-Tg rats indicating mitochondrial distress and dysfunction as a potential underlying cause for neuronal dysfunction in HIV infection. Supported by Yelamanchili Development Funds.
17. Hepatocyte-derived apoptotic bodies induce hepatotoxicity progression in liver cells exposed to HIV and ethanol metabolites. Adepoju, L. A, MS^1^, Bybee, G, BS^1^, Pathania, A. S, PhD^1^, Osna, N. A, MD, PhD^1^; ^1^Department of Pharmacology and Experimental Neuroscience, University of Nebraska Medical Center , Omaha, NE 68198^2^ College of Public Health, University of Nebraska Medical Center , Omaha, NE 68198.
  The development of end-stage liver disease has been reported as an essential outcome of HIV infection, potentiated by second hits, such as alcohol abuse and co-infections. This study aims to identify the mechanisms leading to liver injury progression. Previously, we observed a significant apoptotic cell death in hepatocytes exposed to ethanol and HIV. To elucidate the role of apoptotic bodies (ABs) in communication between liver parenchymal and non-parenchymal cells, Huh 7.5. cells overexpressing ethanol-metabolizing enzyme CYP2E1 were treated with the acetaldehyde-generating system (AGS) and HIVADA and exposed to UV light. Then, ABs were generated by differential centrifugation and tested for cleaved caspase 3, cleaved PARP, LAMP1, calreticulin, and exosome marker CD63. While HIV by itself did not affect caspase 3 and PARP cleavage, AGS reduced their expression, indicating that AGS treatment may provide AB leakage due to pyroptosis or necroptosis. Currently, we are testing the necroptosis marker, MLKL, pyroptosis marker, cleaved gasdermin D expression in hepatocytes and ABs by western blot. Furthermore, when neighboring non-infected hepatocytes internalized these ABs, they up-regulated mRNAs of pro-inflammatory cytokines, TNFa and IL-1b were pushed to apoptosis and pyroptosis as indicated by increased caspase 3 and gasdermin D cleavage. We conclude that the combined exposure of hepatocytes to alcohol metabolites and HIV induces multiple mechanisms of cell death, which further promotes the spread of inflammation and hepatoxicity. Supported by NIAAA, R21AA031928-01.
18. Antiretroviral drugs affect placental matrix metalloproteinases functions. Kumar, M, PhD^1^, Yao, B, PhD^1^, Freel, CI, BS^2^, Summerlin, M, BS^1^, Adalikwu, C, BS^1^, Natarajan, SK, PhD^3^, Anderson-Berry, AL, MD, PhD^4^, Edagwa, BJ, PhD^1^, Gendelman, HE, MD^1^, Bade, AN, PhD^1^; ^1^Department of Pharmacology and Experimental Neuroscience, University of Nebraska Medical Center, Omaha, NE 68198^2^Department of Cellular and Integrative Physiology, University of Nebraska Medical Center, Omaha, NE 68198^3^Department of Nutrition and Health Sciences, University of Nebraska-Lincoln, Lincoln, NE 68583^4^Department of Pediatrics and Division of Neonatology, University of Nebraska Medical Center, Omaha, NE 68198.
  The number of HIV-1-exposed uninfected (HEU) children remain on the rise. Studies showed that HEU children are at increased risk for preterm delivery, infectious morbidity, immune abnormalities, and impaired growth. Yet there are gaps in understanding how in utero antiretroviral drugs (ARVs) exposures impact the health of HEU children across their lifespan. ARVs effects on maternal health and fetal outcomes have been widely studied. However, there is a critical knowledge gap of the effects of ARVs on placental health, a key regulator of fetal development and pregnancy outcomes. Thus, the effects of dolutegravir (DTG) on human term placental explants were evaluated as DTG’s association with abnormal vasculature and preeclampsia was previously reported. DTG’s effect on matrix metalloproteinases (MMPs) activities in placental explants was investigated to assess safety and to determine the potential underlying cause for adverse pregnancy outcomes. Herein, in vitro cultures of placental explants were treated with clinically relevant DTG concentrations or with a vehicle (control). Gelatin zymography was performed on culture media to determine the effect on MMPs activities. Inhibition of both MMP-2 and -9 activities was noted. These observations were validated by DTG-induced reduction in migration and invasion of HTR-8 trophoblastic cells. Overall, we conclude that in utero DTG exposure could impair placental development through reduced MMPs activities. Genetic or co-morbidities-inducing MMPs deficiency may increase the risk for DTG-regimen-affected placental development. Supported by 1R01HD115482-01 (NICHD) and R21HD106842-01 (NICHD).
19. Dopamine modulates nlrp3 inflammasome activation through mitochondrial dysfunction in human macrophages. Daniali, M^1^, Channer, B^1^, Matt, SM, PhD^1^, Kist, T^1^, Gaskill, PJ, PhD^1^; ^1^Department of Pharmacology and Physiology, Drexel University, College of Medicine, Philadelphia, PA 19102.
  Dopamine’s (DA) expanded role beyond neurotransmission and in regulating innate immunity has been investigated recently. Our prior work showed DA (1uM) activates nuclear factor kappa-B (NF-kB) in human monocyte-derived macrophages (hMDMs), triggering IL-1B production through the inflammasome pathway. The NLRP3 inflammasome responds to various damage- and pathogen-associated molecular patterns and can be activated through a two-step process. Inflammasome activation is induced in the second step, mediated by a secondary signal such as mitochondrial damage superoxide. While DA induces inflammasome priming through the NF-κB pathway, our data show that DA simultaneously induces mitochondrial superoxide (MitoSox) production. Based on these, we hypothesized that DA can regulate inflammasome activation via mitochondrial dysregulation. Therefore, we assessed DA-mediated changes in mitochondrial membrane potential and fission in hMDMs, showing that DA-mediated mitochondrial alterations occur independently of inflammasome priming. Also, DA enhances DRP-1 phosphorylation, a GTPase regulating fission, and pharmacological inhibition of DRP-1 phosphorylation reduces DA-mediated inflammasome activation. Further, DA-associated changes in inflammasome activation strongly correlate with alterations in mitochondrial dynamics, highlighting the interdependence of DA signaling, inflammasome activation, and mitochondrial homeostasis. These findings could suggest potential therapeutic targets for conditions involving DA dysregulation, such as Parkinson’s disease and substance use disorders.
20. Bioimaging affirms antiretroviral drug effects on fetal neurodevelopment. Summerlin, M., BS^1^, Kumar, M., PhD^1^, Yao, B., PhD^1^, Adalikwu, C., BS^1^, Foster, E.G., BS^1^, Edagwa, B.J., PhD^1^, Gendelman, H.E., MD^1^, Liu, Y., PhD^2^, Bade, A.N., PhD^1^; ^1^Department of Pharmacology and Experimental Neuroscience, University of Nebraska Medical Center, Omaha, NE 68198 ^2^Department of Radiology, University of Nebraska Medical Center, Omaha, NE 68198.
  The number of HIV-1-exposed uninfected (HEU) children exposed in utero to antiretroviral drugs (ARVs) is increasing. These children suffer from poor neurodevelopment across multiple domains including cognitive, motor, language, and socio-emotional functions. Understanding of in utero ARV-associated neurotoxicity is limited due to the time needed for longitudinal assessments of functional neurodevelopment post-birth. Thus, developing a non-invasive biomarker for early recognition of ARV-linked neurodevelopmental deficits is timely. We posit that novel chemical exchange saturation transfer (CEST) MRI can detect ARV-induced metabolomic alterations in embryo brain. Herein, pregnant C3H/HeJ mice treated daily with dolutegravir (DTG; 50 mg/kg) or vehicle (control) were scanned by a 7T scanner on gestation day 17.5 to acquire CEST maps of embryo brains. CEST scans were acquired using RARE sequence and nonuniform frequency offsets. Data were analyzed with 5-pool Lorentzian fitting. CEST contrast hyperintensities at 3.5 ppm and -3.5 ppm were observed on DTG-exposed embryos brains indicating alterations in glutamate and neuronal membrane lipids, respectively. These indicated DTG-induced developmental neuronal impairments. The average DTG concentration in embryo brain tissue was 575 ng/g. Metabolomics found altered energy and membrane-linked metabolites in DTG-exposed embryos brains validating CEST data. Overall, the study concludes that CEST MRI detects DTG-induced impairments in neuronal development. CEST MRI can be used to assess ARV-associated early-stage neurodevelopmental deficits. Supported by 1R01HD115482-01 (NICHD); R21HD106842-01 (NICHD).
21. HIV-1 proteins and cocaine dysregulate cd34+ progenitor cells differentiation potentially contributing to enhanced systemic inflammation and neuroinflammation. Benmassaoud, MMB, PhD^1^, Cruz, PEC, PhD^1^, Ramirez, SHR, PhD^1^, Andrews, AMA, PhD^1^; ^1^Pathology, Immunology and Laboratory Medicine, College of Medicine, University of Florida, Gainesville, FL 32610.
  People living with HIV (PWH) have an increased risk of cardio-cerebrovascular diseases, which is further elevated by drugs of misuse. CD34+ cells are hematopoietic stem cells (HSC) which are critical for the production and differentiation of blood cells. While HIV infection of CD34+ cells is controversial, the bone marrow contains infected cells which can produce viral proteins or create an inflammatory environment that negatively impact CD34+ activation and differentiation. HSCs are also vulnerable to the effects of drugs of misuse; however, little is known about the shared effects of drugs of misuse in the context of HIV. Here we explored the combined effects of HIV-1 virotoxins (tat or nef) and cocaine on CD34+ cells viability, mitochondrial activity, and differentiation. Our results show that while the cells remain viable, the combination treatment led to an increase in ATP production. Additionally, HIV-1 Tat combined with cocaine resulted in elevated levels of reactive oxygen species, while HIV-1 Nef and cocaine treatment increased mitochondrial activity. Analysis of HSC differentiation using the colony-forming unit (CFU) assay revealed a reduction or impairment in the number of colonies typically formed. Together, these findings suggest that the combination of HIV-1 proteins and cocaine can significantly impair CD34+ cell function. Future studies will explore the effects on cellular respiration and specific lineage differentiation. A better understanding of HSC biology in the context of HIV infection and drugs of misuse could help explain the accelerated CVD in PWH. Supported by DP2DA056172.
22. Higher cerebrospinal soluble CD14 and MIP-1β are associated with neuropathic pain in people with HIV. Sheikh Andalibi, M.S.A., MD^1^, Tavasoli, A.T., MD^1^, Dastgheyb, R.D., PhD^2^, Letendre, S.L., MD^3^, Ellis, R.J.E., MD, PhD^1^; ^1^Department of Neuroscience, University of California San Diego, San Diego, CA 92093 ^2^Departments of Neurology, Johns Hopkins University School of Medicine, Baltimore, MD 21205 ^3^Department of Medicine, University of California San Diego, San Diego, CA 92093.
  Background: People with HIV (PWH) face a higher risk of medical conditions such as metabolic syndrome (MetS), and neuropathic pain (NPP) complicating HIV management. Chronic inflammation is central to these comorbidities. While soluble inflammatory proteins in blood are well-studied, their presence in cerebrospinal fluid (CSF) is less explored. This study investigates associations between inflammatory proteins in CSF and common medical conditions in PWH and people without HIV (PWoH). Methods: CSF samples from 257 participants (126 PWH, 131 PWoH; mean age 38 years) were analyzed. PWH had more males than PWoH (88% vs. 73%, p<0.01) but were similar in age, race, and ethnicity. NPP was defined as burning, aching, or shooting pain in a symmetric, bilateral distribution. Five CSF biomarkers were measured—MCP-1, soluble TNFRII, TNFα, MIP-1β, and soluble CD14 (sCD14). Multivariate logistic regression adjusted for age, sex, HIV status, and HIV-RNA in subgroup analyses of PWH. Results: Among 126 PWH, 44% were virally suppressed with the median current CD4+ T-cell count 683/µL (IQR:366). Compared to PWoH, PWH had a higher prevalence of NPP, dyslipidemia, and MetS (p<0.05). Higher CSF sCD14 and MIP-1β was associated with NPP (adjusted OR [95% CI]: 2.8 [1.31–6.6]). Conclusion: CSF sCD14 and MIP-1β levels are associated with NPP in PWH, underscoring the role of myeloid activation. These biomarkers could aid in identifying individuals who have NPP and be therapeutic targets to treat NPP. Developing inhibitors for myeloid activation and other immunomodulatory therapies may be effective.
23. Hamster model of post-acute sequelae of SARS-COV-2 (pasc) parallels with human pasc. Kumar, N, PhD^1^, Sardarni, U K, PhD^1^, Balasrinivasa, S, PhD^2^, Samuelson , M M, PhD^3^, Acharya, A, PhD^1^, Byrareddy, S N, PhD^1^; ^1^Department of Pharmacology and Experimental Neuroscience, University of Nebraska Medical Center, Omaha, NE 68198 ^2^Department of Radiology, University of Nebraska Medical Center, Omaha, NE 68198 ^3^Animal Behavior Core, University of Nebraska Medical Center, Omaha, NE 68198.
  Some COVID-19 patients experience several chronic conditions including fatigue, mood swings, sleep disorders, loss of smell and taste, and cognitive impairments collectively termed neurological manifestations of post-acute sequelae of COVID-19 (neuro-PASC). Recent evidence suggests that individuals with neuro-PASC may develop AD and related dementia (ADRD) with unknown etiology. To understand tissue-specific insult and contributing factors for ADRD development, we have developed a Golden Syrian Hamsters (GSH) model of PASC. Young and old GSHs were inoculated with SARS-CoV-2 and monitored for 35 days post-infection to model PASC in humans. The viral shedding in oral fluids is seen as similar to humans. At necropsy, we observed a detectable viral RNA only in the lung of a subset of animals. The transcriptomic analysis of the brain identified differentially expressed genes associated with the enrichment of several pathways linked with microglia activation, neuroinflammation, and reduction of its phagocytosis capacity, which are implicated in the development of ADRD. The Behavioural studies indicate the development of elevated compulsive behaviours at acute infection, which persists during neuro-PASC. Next, DTI indicates a reduction in the fractional anisotropy in the cerebral cortex and hippocampus, suggesting presence of microstructural changes during neuro-PASC. These findings indicate that similar to neuro-PASC patients, an inflammatory milieu persists in the hamster’s brain with metabolomic and transcriptomic signatures of development of ADRD-related illness.
24. EcoHIV shifts dendritic branching in pyramidal neurons irrespective of the effect of Acod1. Ye, QY, MS^3^, Li, HL, PhD^1^, Mactutus, CF, PhD^1^, Korunova, EK, PhD^2^, Sikirzhytski, AS, PhD^2^, Frizzell, NF, PhD^2^, Shtutman, MS, PhD^2^, Booze, RM, PhD^1^; ^1^Cognitive and Neural Sciences Laboratory, Department of Psychology, University of South Carolina, Columbia, SC 29208 ^2^Department of Drug discovery and Biomedical Sciences, College of Pharmacy, University of South Carolina, Columbia, SC 29208 ^3^Department of Biological Sciences, College of Arts and Sciences, University of South Carolina, Columbia, SC 29208.
  HIV-1-associated neurocognitive disorders (HAND) is a clinically significant complication of HIV infection. We found that HIV infection upregulated the microglia-specific gene, aconitate decarboxylase 1 (Acod1). Acod1 can produce itaconate, an anti-inflammatory factor protecting macrophages from infection-triggered death. Our preliminary results showed that the permeable modified derivatives of itaconate – dimethyl itaconate (DMI) and 4-octyl-itaconate (4-OI) protect primary neurons from the toxicity of HIV-Tat. However, the exact role of Acod1 and its product in protecting neurons from HIV remains unclear. In a previous study, we found EcoHIV infection significantly altered pyramidal neuron morphology in medial prefrontal cortex (mPFC). Here, we knocked out the Acod1 gene to investigate its effects on pyramidal neuron morphology after HIV infection. Acod1-/- and wild-type C57BL/6 mice were injected retro-orbitally with or without EcoHIV and sacrificed two weeks later. Ballistic labeling was used to visualize pyramidal neurons in the mPFC. Neurolucida 360 was utilized to perform Sholl analysis. Presently, EcoHIV significantly altered intersections in both wild-type and Acod1-/- mice (Regression fit, R2s >0.95, F(4, 39)=15.7, p≤0.001). Specifically, EcoHIV caused a shift in the distribution of dendritic branching from more proximal to more distal locations. However, Acod1 knock out did not affect intersections in control or EcoHIV injected mice (Regression fit, R2s >0.94, F(4,39)=0.47, p>0.10). Supported by DA059310, AG082539, DA058586, GM109091.
25. Fentanyl induced lipid droplet-accumulation in microglia – a biomarker for neuroinflammation. Das, RK, PhD^1^, Ghazanfarpour, S, MS^2^, Aalinkeel, R, PhD^1^, Sharikova, AV, PhD^2^, Prasad, PN, PhD ^1^, Khmaladze, A, PhD^2^, Mahajan, SD, PhD^1^; ^1^Department of Medicine, SUNY University at Buffalo, Buffalo, NY 14203 ^2^Department of Physics, SUNY University at Albany, Albany, NY 12222.
  Fentanyl is a highly potent analgesic, but its abuse can lead towards physical dependency and addiction, which increases the risk for overdose and death. Fentanyl’s high lipophilicity results in its increased accumulation in the brain, causing severe CNS cytotoxicity, resulting in microglial dysfunction, increased cell apoptosis, contributing to neurocognitive impairment. Raman spectroscopy is a label-free vibrational spectroscopic technique, used to examine cellular/biochemical changes that occur when a cell undergoes apoptosis. Implementing Raman-based imaging, the goal of our study was to accomplish real-time understanding of chemical changes after fentanyl impact and unfold underlying biochemical changes in microglial apoptosis. Our results exhibit fentanyl overdose induced oxidative stress and its functional consequences in microglia offering significant insights into the role of mitochondria in neuropathology. Fentanyl induces significant changes in lipid chemistry attributed to changes in hybridization of phospholipids and mobilization of lipids from the Lipid droplets (LD). The drug-induced increase in LD in microglia, termed lipid droplet-accumulating microglia (LDAM), can produce high ROS levels, secrete pro-inflammatory cytokines, and simultaneously contribute to defective phagocytosis, leading to neuroinflammation and hyperalgesia. Thus, the approach facilitates non-invasive real time monitoring of fentanyl-induced apoptosis in human microglia, highlighting mechanisms that underlie its neurotoxicity and lead towards development of novel therapeutic targets for its cure. Supported by NIH-National Institute of Drug Abuse (Grant # 5R01DA047410-02).
26. Muscle building supplement, β-hydroxy β-methyl butyrate, modulates neuroplasticity in an Alzheimer’s mouse model. Paidi, Ramesh Kumar, PhD^1^, Pahan, Kalipada, PhD^1^; ^1^Department of Neurological Sciences, Rush University Medical Center, Chicago, IL 60612 ^2^Division of Research and Development, Jesse Brown Veterans Affairs Medical Center, Chicago, IL 60612.
  Alzheimer’s disease is a common form of dementia. It has an unclear etiology, but several behavioral, genetic, and environmental factors are known to significantly contribute to the disease progression. However, one of the main neuropathology’s associated with Alzheimer’s disease is thought to be synapse loss. β-hydroxy β-methyl butyrate (HMB) is a typical supplement used by bodybuilders to increase the strength and muscle growth brought on by training. Even after prolonged use, HMB is a safe supplement with no negative side effects. Initially, we evaluate the HMB’s impact on neuronal plasticity and neuronal markers in primary mouse hippocampal neuronal cells. We also administered oral HMB to six-month-old XFAD mice for 30 days and evaluated their cognitive abilities using the T-Maze, Barnes Maze, Novel Object Recognition, and locomotor tests. Western blotting and immunohistochemistry were used to assess in vivo pathway inductions. Neuronal cells from the hippocampus showed more of the neuronal plasticity markers Glut N2A, Glu-A1, Snap 25, and PSD 95 after being treated with HMB. Oral HMB treatment enhances cognitive performance in 5xFAD AD mice. SNAP25 and PSD 95 expressions increased after the HMB administration. Improved synaptic plasticity is associated with increased calcium influx and CREB phosphorylation in the hippocampal region. our findings illustrate, HMB promotes synaptic protein expression and regulates CREB phosphorylation, potentially regulating brain plasticity and leading to cognitive recovery in AD models. Supported by This work was supported by grants (AT10980, AT10980-01S1, and AT10980- 03S1) from NIH.
27. Cocaine increases HIV pathogenesis for a dopamine-independent mechanism. Luu, R, BS^1^, Eugenin, E, PhD^1^; ^1^Dept. of Neurobiology, University of Texas Medical Branch at Galveston, Galveston, TX 77555.
  Cocaine is the second most abused recreational drug. In addition to overdoses, cocaine is associated with inflammation, immunological suppression, and worsened infectious diseases, including HIV. Within the brain, cocaine causes both acute and chronic imbalance of neurobiochemistry by inducing chronically elevated neurotransmitters such as dopamine and preventing its reuptake at the neuronal cleft. However, in the immune system, cocaine suppresses cell function and promotes a pro-inflammatory state. Elevated levels of extracellular dopamine in response to cocaine increased susceptibility to HIV viral entry and neuroinflammation but the mechanism of cocaine-inducing immune/HIV dysregulation is poorly explored. We hypothesize that cocaine alone induces a transcriptionally active state within peripherally circulating immune cells that makes them susceptible to HIV infection and higher viral replication by a dopamine-independent mechanism. Here, we demonstrate that circulating levels of catecholamines in healthy, HIV, HIV+ART, and drug users are minimal and not correlated with variables such as drug status, immune compromise, or replication, suggesting an alternative mechanism of immune activation. Treatment of HIV-infected PBMCs with cocaine enhanced viral replication by an independent dopaminergic mechanism because blocking DAT or D1/D2 receptors on immune cells did not alter the enhanced replication elicited by cocaine. In conclusion, cocaine is able to induce viral replication in a manner independent of soluble dopamine or its receptors. Supported by The National Institute of Mental Health and the National Institute of Neurological Disorders and Stroke.
28. Epigenetic regulation of NLRP6 inflammasome-mediated neuroinflammation in HIV TAT and ethanol-exposed astrocytes. Singh, S, PhD^1^, Buch, S, PhD^1^, Periyasamy, P, PhD^1^; ^1^Department of Pharmacology and Experimental Neuroscience, University of Nebraska Medical Center, Omaha, NE 68198
  Nearly half of people living with HIV (PLWH) are alcohol abusers, placing them at a 2–3-fold higher risk of developing HIV-associated neurological disorders (HAND) which significantly impact public health. Our group has recently identified that the astrocyte-specific inflammasome NLRP6 plays a critical role in HIV Tat and ethanol-induced neuroinflammation. Based on the premise that alcohol use exacerbates HIV-associated neuroinflammation, we explored the epigenetic regulation of NLRP6-mediated neuroinflammation in HIV Tat and ethanol-exposed astrocytes. We demonstrated that exposure of mouse primary astrocytes (mPAs) to HIV Tat (50 ng/mL) and ethanol (50 mM) synergistically activated astrocyte markers and the NLRP6 inflammasome. This combined exposure also increased NLRP6 downstream signaling mediators-caspase-1, and proinflammatory cytokines- IL-1β and IL-18. Notably, silencing NLRP6 expression using siRNA inhibited these effects, confirming its pivotal role in HIV Tat and ethanol-mediated neuroinflammation. Additionally, our miRNA microarray study revealed significant downregulation of miR-339, which was confirmed to target the 3’-UTR of NLRP6 mRNA using TargetScan and argonaute immunoprecipitation assays. Overexpression of miR-339 in mPAs exposed to HIV Tat and ethanol validated its role in the epigenetic regulation of NLRP6 signaling. In summary, our findings provide novel insights into the miR-339-mediated epigenetic regulation of NLRP6 activation in HIV Tat and ethanol-exposed astrocytes, advancing our understanding of HAND pathogenesis in the context of alcohol use.
  Supported by Startup funding from the UNMC to PP, NIAAA (AA031444; P50AA030407-5126, Pilot Core grant) to SS. and NCSAR.
29. Colostrum extracellular vesicle therapies for Parkinson’s disease. Akter, SA, MS^1^, Hollingsworth, DH, BS^1^, Srivastava, SS, BS^1^, Dey, SSD, PhD^1^, Panja, SP, PhD^1^, Kumar, MK, MS^1^, Du, XU, MS^1^, Saha, AS, BS^1^, Yeapuri, PY, PhD^1^, Bhattarai, SB, MS^1^, Foste, EF, BS^1^, Kadry, RK, MS^1^, Oludipe, DO, BS^1^, Ehrenkranz, EE^1^, Hu, GH, PhD^1^, Mosley, RLE, PhD^1^, Oehlerking, JO, BS^2^, Swarts, KS, BS^2^, Gendelman, HE, MD^1^, Sil, SS, PhD^1^; ^1^Department of Pharmacology & Experimental Neuroscience, University of Nebraska Medical Center, Omaha, NE 68198 ^2^Oehlerking Farm, Oehlerking Farm, Elmwood, NE 68349.
  Parkinson’s Disease (PD) is the second most common progressive neurodegenerative disease. A pathological hallmark is nigrostriatal degeneration and the formation of Lewy bodies. Microglial neuroinflammation is critical in PD progression amongst genetic and environmental factors. Colostrum EVs (CEVs) were studied based on their anti-inflammatory properties in neurological diseases. Based on the intense interests of our laboratories in developmental PD therapeutics, we tested CEVs for their neuroprotective and anti-inflammatory properties in the 1-methyl-4-phenyl-1,2,3,6-tetrahydropyridine (MPTP) e model. CEV-injected MPTP mice were studied during the maximum neuroinflammatory activities intoxication. Brain tissues were collected from the midbrain, the brain sub-regions affected in human disease. RNA was isolated and sequencing was performed. Transcriptomic analyses followed this to assess the differential gene expression and pathway enrichments. Gene expression associated with inflammatory pathways demonstrated infiltration and activation of immune cells. CEV treatments demonstrated control of microglial activation pathways and neuroprotection. Decreased pro-inflammatory cytokines and chemokines as well as suppressed canonical inflammasomes were recorded during CEV-treatments. Each was linked to immune-regulatory transcription factors, proteins, and receptors associated with neuroinflammation. These results will be discussed as they are linked to broad pathways for neuronal protection. The University of Nebraska Foundation, including donations from the Margaret R. Larson Professorship supported this research.
30. Induction of interferon-stimulated genes (ISGs) is differentially affected by HBV and alcohol in hepatocytes and macrophages. Bhat, SB, PhD^1^, Chava, HC, MS^1^, Bybee, GB, BS^1^, Pathania, AP, PhD^1^, Osna, NO, MD, PhD^1^; ^1^Department of Pharmacology and Experimental Neurosciences, University of Nebraska Medical Center, Omaha Nebraska
  About 296 million people worldwide live with chronic hepatitis B viral (HBV) infection, and alcohol potentiates the outcomes to end-stage liver diseases. HBV replicates in hepatocytes, but other liver non-parenchymal cells can sense the virus. The products of several APOBEC genes from the ISG family regulate HBV DNA expression and cccDNA cleavage. Here, we studied the IFN-triggered induction of APOBEC3G, 3A, and 3B in HepAD38 cells and monocyte-derived macrophages (MDM) and the effects of ethanol on APOBEC expression. APOBEC induction was performed by exposure to 200–400 IU of IFNa, in the presence or absence of an acetaldehyde-generating system (AGS) for HepAD38 cells and/or 250 DE HBV and 25 mM ethanol for MDMs and then tested by RT-PCR. In HepAD38 cells, we found no expression of APOBEC 3A, low induction of 3B and significant induction of 3G, which AGS suppressed. This corresponds to an AGS-potentiated increase in HBV DNA and cccDNA. In MDMs, all APOBEC isoforms were induced by IFNa, but ethanol suppressed only 3G expression corresponding to the up-regulation of HBV RNA levels. All APOBEC mRNAs were secreted with exosomes and detected in MDM’s exosome cargo. We conclude that IFNa-induced APOBEC3G regulates HBV replication in hepatocytes, but its effects on cccDNA cleavage might be strengthened by the delivery of 3A and 3B with exosomes from IFNa-stimulated macrophages. Supported by NIAAA P50AA030407-5129 (PI- N. Osna).
31. Oral-blood microbial translocation in chronic cocaine users: implications for cocaine-altered barrier integrity. Johnson, D, MS^1^, Wan, Z, MS^1^, Salman, T, PhD^1^, Jiang, W, MD^1^; ^1^Department of Pharmacology and Immunology, Medical University of South Carolina, Charleston, SC 29425 ^2^Clinical Science Research and Development, Ralph H. Johnson VA Medical Center, Charleston, SC 29425 ^3^Division of Infectious Diseases, Department of Medicine, Medical University of South Carolina, Charleston, SC 29425.
  Chronic cocaine use impacts systemic inflammation either directly or indirectly. This study examines oral-to-blood microbial translocation as a potential driver of peripheral inflammation in chronic cocaine users. Paired saliva and plasma samples were collected from chronic cocaine users and age- and sex-matched non-drug users and analyzed using microbial 16S rRNA V4 sequencing. Cocaine users exhibited reduced α and distinct β diversities in saliva but not in plasma versus controls (Mann-Whitney U test), suggesting localized microbiome dysbiosis. The most abundant oral microbiome was the Streptococcus genus, and the least abundant microbiome was Neisseria in cocaine users versus controls. Notably, only the Streptococcus genus and Streptococcus species (i.e., S. parasanguinis) are the predominant microbiomes sharing at the two sites in cocaine users (p < 0.05) but not in controls, indicating species-specific translocation. Although saliva Neisseria was more abundant in controls versus cocaine users, some Neisseria species were detected in the blood of cocaine users but not controls, indicating a compromised barrier in cocaine users. Finally, in vitro studies demonstrated cocaine promoted the growth of S. parasanguinis; human PBMCs produced IL-6, IL-1β, and TNF-α in response to inactivated S. parasanguinis (p < 0.05); however, adding cocaine did not alter cytokine levels. Our findings suggest that cocaine use-increased Streptococcus and its species in the oral cavity may translocate into circulation via the compromised oral-to-blood barrier and promote systemic inflammation. Supported by NIDA (R01DA055523 and R03DA057164); USDVA CSR&D Service (CX00242); MUSC (Odyssey Pre-doctoral Fellowship).
32. Methamphetamine increases metallothionein expression in HIV infected macrophages: Potential contributions to HIV-NCI. Weiselberg, J, MS^1^, Hernandez, C, PhD^1^, Niu, M, PhD^2^, Fox, H, MD, PhD^2^, Calderon, T, PhD^1^, Berman, JW, PhD^1^; ^1^Pathology Department, Albert Einstein College of Medicine, Bronx, NY 10033 ^2^Department of Neurological Sciences, University of Nebraska Medical Center, Omaha, NE 68198.
  HIV associated neurocognitive impairment (HIV-NCI) occurs in 15–50% of people with HIV (PWH) despite viral suppression with antiretroviral therapy. Methamphetamine (meth) use by PWH exacerbates HIV-NCI. HIV-NCI develops in part due to the entry of HIV infected monocytes into the central nervous system (CNS), that can differentiate into macrophages, establishing a persistent viral reservoir. Macrophages are important immune cells that perform myriad functions to maintain tissue homeostasis. We hypothesize that long term treatment with meth results in macrophage dysfunction and exacerbates HIV-NCI. Bulk RNA sequencing of primary human macrophages infected with HIV and treated with meth daily for five days showed increased expression of metallothionein 1 (MT1) in five independent donors. These findings were validated in additional donors by qPCR. MT1 neutralizes ROS and translocates to the nucleus, resulting in cytokine production. Increased ROS and inflammatory cytokines are hallmarks of HIV-NCI. Increased MT1 has been shown in conditions of neurocognitive impairment, but there are no studies in the context of HIV or substance use. We performed immunofluorescence staining and confocal microscopy to quantify expression and localization of MT1 in the cytoplasm and nucleus and showed an increase in nuclear translocation of MT1 with meth treatment. Ongoing studies include cytokine and ROS assays. Long term meth treatment may impair HIV infected macrophage functions due to functional changes of MT1, contributing to neuroinflammation and CNS injury that result in HIV-NCI. Supported by NIDA.
33. Mechanisms contributing to inflammasome modulation of mu-opioid receptor (Mu-or) agonists morphine and fentanyl on neuronal adaptation and behavioral activity. Veeragoni, DV, PhD^1^, Rodriguez, M, PhD^1^, Eans, SO, PhD^2^, Carbajal, C, PhD^1^, Owens, F, PhD^1^, McLaughlin, JP, PhD^2^, El-Hage, N, PhD^1^; ^1^Department of Cellular and Molecular Medicine, Herbert Wertheim College of Medicine. Florida International University, Miami, FL 33199 ^2^Department of Pharmacodynamics, College of Pharmacy. University of Florida, Gainesville, FL 32610.
  Mu-opioid receptor agonists morphine and fentanyl are effective analgesics, with fentanyl being 100 times more potent. However, they can cause adverse effects like respiratory depression. We studied how morphine or fentanyl exposure affects genes and proteins related to synaptic plasticity and the inflammasome in pain-activated brain regions. C57BL/6J mice received i.p. injections of morphine (10 mg/kg) or fentanyl (0.1 mg/kg), revealing maximal antinociception 30 minutes post-injection. Brain analysis 4 or 24 hours after treatment showed fentanyl alone reduced MOR-1 RNA expression, while both drugs significantly downregulated AMPA and NMDA glutamate receptors levels and PSD-95 expression compared to saline. The combined treatment elevated NF-κB and MAPK p38 expression versus single-drug or saline treatments. These alterations corresponded with increased IL-1β, IL-6, IL-18, MCP-1, and RANTES cytokines and inflammasome-related gene expression like Casp1 and Nlrp3. To further assess the inflammasome’s role, mice were pretreated with an NLRP3 inhibitor MCC950 (40 mg/kg, i.p.) before morphine or fentanyl administration. NLRP3 inhibition enhanced both drugs’ antinociception and respiratory depression, along with morphine-conditioned place preference. These findings suggest a new mechanism of inflammasome modulation affecting morphine and fentanyl’s neuronal adaptation, synaptic impairment, and opioid-induced behavioral effects. Supported by National Institutes of Health R01DA057884 and R01DA057145 to NEH and JPM.
34. Characterizing epigenetic modulation of inflammation and synaptic plasticity in the development of HIV and opioid-induced CNS disorders. Owens, F^1^, Souchak, J^1^, Carbajal, C, MS^1^, Cesaroni, E^1^, Dimitroff, C, PhD^1^, Lima, F, PhD^2^, Rodriguez, M, PhD^1^, El-Hage, N, PhD^1^; ^1^Department of Cellular and Molecular Medicine, Florida International University, Miami, FL 33199 ^2^Department of Chemistry and Biochemistry, Florida International University, Miami, FL.
  We’ve previously shown that HIV infection and morphine exposure reduce antiretroviral (ART) effectiveness, increasing viral replication and inflammation, and elevating histone-modifying enzymes in HIV-infected astrocytes. In postmortem brain tissue from HIV patients with HAND and substance use disorder, we found dysregulated expression of immediate early response genes linked to synaptic plasticity, enhanced extracellular matrix remodeling, Alzheimer’s-related signaling pathways, and persistent neuroinflammation associated with chromatin remodeling. In this study, we used an EcoHIV-infected mouse model with opioid use disorder to explore changes in neural activity, immune responses, epigenetics, and behavior in both male and female mice. Over nine weeks, infected mice on ART and exposed to morphine showed cognitive decline (Y-maze), reduced motor skills (horizontal bar) in females, and increased antinociception (hot plate) in females but not males. Flow cytometry revealed initial increases in neurotransmitter receptors DAT and NMDAR1 after two weeks, followed by a decrease after one month of morphine exposure in EcoHIV, ART, and morphine-treated mice – a trend not seen in other groups. Similar to human brain tissue, we also observed sustained innate immune responses and chromatin remodeling in mice treated with EcoHIV, cART, and morphine using a combination of ELISA, mass spectrometry, PCR, and western blotting. Overall, this data helps characterize the environment contributing to the development of HAND and may offer potential therapeutic targets in the future. Supported by National Institutes of Health R01DA057884 and R01DA057145 to Nazira El-Hage.
35. Chrysin attenuates cardiac diastolic dysfunction in HIV-infected humanized mice treated with anti-retroviral drugs. Garcia, JV, BS^1^, Namvaran, A, PhD^1^, Ramasamy, M, PhD^1^, Hackfort, BT, PhD^2^, Dash, P, PhD^1^, Edagwa, B, PhD^1^, Gorantla, S, PhD^1^, Bidasee, KR, PhD^1^; ^1^Pharmacology and Experimental Neuroscience, University of Nebraska Medical Center, Omaha, NE 68198 ^2^Cellular and Integrative Physiology, University of Nebraska Medical Center, Omaha, NE 68198.
  Antiretroviral drug treatment has successfully lowered HIV-1 viremia, but people living with HIV-1 infection (PLWH) continue to be plagued with early-onset cardiac diastolic dysfunction (DD). Studies suggest that the DD is due to myocardial ischemia and fibrosis. In ischemic environments, cellular breakdown of HIF-1a is reduced, allowing it to translocate to the nucleus and induce expression of metabolic genes. However, persistent elevation in HIF-1a is deleterious. Chrysin is a dietary flavone that exhibits anti-HIV activity and HIF-1a inhibition. Here we investigated whether chrysin would attenuate DD in HIV-1 infected Hu-mice treated with DTG/TDF/FTC for 8 weeks using echocardiography. Fifteen mice were infected with 5 X 106 viral particles of HIV-1ADA. Four weeks later, infected animals were randomly divided into three groups and treated with DTG/TDF/FTC, DTG/TDF/FTC/chrysin or no treatment for 8 weeks hu-mice with and without chrysin treatment served as controls. After 12 weeks of infection, E:A ratio, E-wave deceleration time, IVRT and MV ET increased in Hu-mice indicative of diastolic dysfunction. Ejection fraction and fractional shortening also decreased, suggestive of systolic dysfunction. Treating HIV-1 infected Hu-mice with DTG/TDF/FTC blunted systolic but not diastolic dysfunction. Treating HIV-1 infection mice with DTG/TDF/FTC/chrysin for 8 weeks blunted both systolic and diastolic dysfunction. These data are the first to supplementing the dietary flavone Chrysin to DTG/TDF/FTC-treated HIV-1 infected mice was cardio-protective. Funded in part by NIH.
36. Alcohol exposure disrupts human cerebral organoid development and neurogenesis. Donadoni, M, PhD^1^, Cakir, S^1^, Wolf, M^1^, Swingler, M^1^, Sariyer, IK^1^; ^1^Department of Microbiology, Immunology and Inflammation, Center for Neurovirology and Gene Editing, Temple University Lewis Katz School of Medicine, Philadelphia, PA.
  Alternative splicing of genes in the brain can modulate protein functions, potentially influencing alcohol-induced neurotoxicity. Recent developments in induced pluripotent stem cell (hiPSC) have enabled the creation of 2D and 3D culture models that hold promise as models for studying effect of alcohol on brain development and function. To explore the effects of alcohol on brain development, we generated neural progenitor cells (NPCs) and human cerebral organoids (hCOs), from hiPSCs. We showed that exposure of ethanol at different stages of hCO development impacts their viability and growth. Early exposure resulted in complete loss of viability, whereas delayed exposure led to reduced size and highly compromised structural integrity. Histological analysis showed loss of neuronal culture in mature hCOs. Further, hiPSCs, NPCs and immature hCOs were particularly sensitive to alcohol-induced toxicity, while young and mature hCOs were resistant. Our previous studies demonstrated that ethanol exposure causes alternative splicing of Mcl-1, which plays a crucial role in the neurotoxicity linked to alcohol exposure in the developing brain. Through analysis of Mcl-1 pre-mRNA splicing, we demonstrated that ethanol significantly decreases the Mcl-1L/Mcl-1S ratio in hiPSCs and NPCs, with no observable effect on mature hCOs. These results confirm the critical role of Mcl-1 in alcohol-induced toxicity and indicate that Mcl-1L protects against ethanol toxicity in hiPSCs and NPCs, uncovering a novel mechanism underlying the increased sensitivity of progenitors to the toxic effects of EtOH.
37. APOE4 genotype exacerbates SARS-COV-2 neuropathogenesis in a sex-specific manner. Narayanan, M^1^, Krishna, VD^1^, Arju, T^1^, Chang, A^2^, Korthas, H^2^, Li, L^2^, Low, WC^3^, Cheeran, MCJ^1^; ^1^Veterinary Population Medicine, College of Veterinary Medicine, University of Minnesota, St. Paul, MN 55108 ^2^Department of Experimental and Clinical Pharmacology, College of Pharmacy, University of Minnesota, Minneapolis, MN 55455 ^3^Department of Neurosurgery, Medical School, University of Minnesota, Minneapolis, MN 55455.
  The neurological effects of SARS-CoV-2 infection are particularly pronounced in older individuals and evidence suggests that SARS-CoV-2 can disrupt molecular pathways linked to neurodegeneration, exacerbating neuroinflammation and cognitive decline. The APOE4 genotype is a major genetic risk factor for Alzheimer’s disease (AD). Clinical data indicate that APOE4 carriers are more susceptible to SARS-CoV-2 infection. However, the impact of APOE4 genotype on SARS-CoV-2 neuropathogenesis remains poorly understood. We hypothesized that APOE4 genotype worsens SARS-CoV-2 neuropathogenesis, leading to cognitive and neurodegenerative defects. To test this, we infected human APOE4 and APOE3 knock-in mice intranasally with SARS-CoV-2 and assessed viral pathogenesis and neuroinflammatory responses. Our results show that APOE4 mice exhibit delayed viral clearance and higher viral load in the lungs, with differences between male and female mice. Despite the absence of infectious virus in the brain, APOE4 mice displayed a slow, persistent increase in cytokines TNF-a and IL-1b compared to the control APOE3 mice. Microglia / macrophage activation markers were upregulated at 14 days post-infection, suggesting chronic neuroimmune activation. Ongoing studies are focused on the impact of APOE4 on the blood-brain barrier, and long-term cognitive and neurodegenerative defects. These findings highlight the interaction between the APOE4 genotype and SARS-CoV-2 neuropathogenesis, offering potential pathways for therapies to mitigate cognitive decline and neurodegeneration in vulnerable populations. Supported by NIH / NIA (RF1AG077772; AG056976; AG058081).
38. Induction of differentially spliced OPRM isoform MOR-1X by opioids and HIV-1 and its role in downstream signaling and dependence. Swingler, M^1^, Donadoni, M^1^, Cakir, S^1^, Kumar, V^2^, Bishir, M^3^, Huang, W^3^, Chang, SL^3^, Sariyer, IK^1^; ^1^Department of Microbiology, Immunology, and Inflammation, Center for Neurovirology and Gene Editing, Temple University Lewis Katz School of Medicine, Philadelphia, PA 19140 ^2^Lewis Katz School of Medicine, Temple University, Philadelphia, PA 19140 ^3^Institute of NeuroImmune Pharmacology, Seton Hall University, South Orange, NJ 07079.
  Clinically used opioids, such as morphine, are known to act on the mu opioid receptor (MOR) coded by the Opioid Receptor Mu 1 (OPRM1) gene which undergoes extensive alternative splicing. Our previous studies have shown that the MOR-1X isoform is induced in response to morphine in a neuroblastoma cell line. With the development of 3D model systems such as human cerebral organoids (hCO), we have access to more comprehensive and representative in vitro models of the human brain. Here, we investigate the effects of opioids and HIV-1 infection on OPRM1 pre-mRNA splicing in a hCO model and examined the downstream effects of morphine in HEK293 cells expressing either MOR-1 or MOR-1X. Our results show that acute morphine treatment induces the MOR-1X isoform in hCOs in a dose dependent manner. Interestingly, HIV-1 infection alone induces MOR-1X, with a further induction following morphine treatment in addition to infection. Our cell-based assay revealed that both MOR-1 and MOR-1X significantly reduce cAMP signaling to the same extent following acute morphine treatment. However, following withdrawal from morphine, MOR-1X shows a significant increase in cAMP superactivation, a hallmark of opioid dependence. Lastly, our RNAseq data and IPA analysis revealed a variety of differentially regulated genes when comparing the activation of MOR-1 and MOR-1X by morphine, suggesting that these isoforms have different downstream effects. Taken together, this data suggests that the inducible MOR-1X isoform may play a role in dependence by having differential downstream effects compared to MOR-1.
39. Alcohol and e-cigarette exposure induce release of extracellular vesicles and their plasminogen urokinase content promoting Blood Brain Barrier injury (BBB). Mekala, NK, PhD^1^, Togre, NS, PhD^1^, Rom, S, PhD^1^, Sriram, U, PhD^1^, Persidsky, Y, MD, PhD^1^; ^1^Department of Pathology and Laboratory Medicine, Lewis Katz School of Medicine, Temple University, Philadelphia, PA 19140.
  Previously, we demonstrated that chronic exposure to alcohol (EtOH) and e-Cig stimulates P2X7receptor (P2X7r) leading to extracellular vesicle (EV) release from human pulmonary alveolar epithelial cells (PAEC). EVs amount and content depended on the stimulants. Here, we assessed EV content by proteomic analysis, evaluated EV paracrine signaling in human brain microvascular endothelial cells (BMVEC) and correlated with changes in isolated brain microvessels from mice chronically exposed to alcohol (EtOH). EVs were isolated from media of PAEC exposed to EtOH (100mM), aldehyde ALD (100µM), and e-Cig conditioned media (1.8% nicotine). Proteomic analysis demonstrated a significant increase in several proteins, notably in plasminogen urokinase activator (PLAU), known to break the extracellular matrix (ECM). Paracrine signaling capability of EVs was shown in BMVEC by decreased trans-endothelial resistance (TER, measuring barrier function) and intracellular Ca2+ release. Pretreatment of PAEC with P2X7r inhibitor led to diminution of PLAU content in EVs, TER amelioration and diminished Ca2+ release in BMVEC. A significant increase of PLAU, MMP 2, MMP9 gene expression and lower TIMP1 expression in microvessels confirmed BBB damage in vivo. Animal treatment with P2X7r inhibitor resulted in normalization of PLAU, MMP2, MMP9 and TIMP1 gene expression in isolated brain microvessels. In toto PLAU transported by PAEC derived EVs can act as paracrine signal and negatively affect the BBB integrity confirming lung-brain cross talk during EtOH and e-Cig exposure and regulatory role of P2X7r. Supported by NIAAA; 5R01AA030841-02.

## Investigator/General Abstracts

### 1 HIV infection of human iPSC-derived cortical organoids reveals a robust inflammatory response and neurodegenerative phenotype

Agarwal, Y, BS^1^, Ogbolu, V, MS^2^, Montilla, J, BS^3^, Kist, T, BS^1^, Matt, S, PhD^1^, Qiang, L, MD, PhD^2^, Gaskill, PJ, PhD^1^; ^1^Pharmacology & Physiology, Drexel University College of Medicine, Philadelphia, PA, 19102 United States. ^2^Neurobiology & Anatomy, Drexel University College of Medicine, Philadelphia, PA, 19129 United States. ^3^Interdisciplinary Health Sciences, Drexel University Graduate School of Biomedical Sciences and Professional Studies, Philadelphia, PA, 19104 United States.

HIV enters the CNS rapidly, establishing a viral reservoir in myeloid cells such as macrophages and microglia. Persistent inflammation associated with low levels of HIV replication drive neurodegenerative processes in the brain, such as decreased synapse and dendritic integrity. These effects can be altered and amplified in the presence of addictive substances and neurotransmitter dysregulation. Examination of these processes is limited due to challenges associated with modeling HIV infection in the human CNS. Here, we build on our prior data showing in vitro infection of human induced pluripotent stem cell (iPSC)-derived microglia (iMg) by developing a tractable iPSC-derived model of cortical organoids that incorporates our iMg. Our data show that iMg integrate into cortical organoids, which also contain astrocytes and glutamatergic neurons, and remain within organoids for over two months. Analysis of p24Gag staining and secretion show that the organoids support infection, which is restricted to iMg. Viral replication increases significantly over the first 8–11 days of infection, decreases by day 20, and then stabilizes for the remainder of the experiment. Infection correlates with increases in cytokine secretion (CXCL10, IL-6), changes in microglial morphology, and changes in dendrites and synapses. This indicates that inflammation and neurodegeneration persist despite low levels of replication, as observed in vivo. Future studies will examine the effects of neuropsychiatric agents and neurotransmitters on viral kinetics, inflammation, and associated neurodegeneration.

### 2 CCR5 Receptor Targeted Lipid Nanoparticles for HIV-1 Proviral DNA Elimination

Ali, MU, BS^1^, Panja, S, PhD^1^, Chaudhary, BN, MS^1^, Dey, SS, MS^1^, Zaman, LA, PhD^1^, Patel , MK, PhD^1^, Gendelman, HE, MD^1^; ^1^Pharmacology and Experimental Neuroscience, University of Nebraska Medical Center, Omaha, NE, 68198 United States.

In the therapeutic arena of HIV-1, the development of antiretroviral therapy (ART) has significantly reduced disease mortality. However, the goal of achieving a functional cure has not yet been realized, as ART cannot eradicate the multiple latent viral reservoirs that exist across various anatomical locations. Latency development in HIV-1 infection is associated with an exclusive increase in CCR5 receptor expression in the latently infected cells. As a result, the CCR5 receptor presents as a promising target for delivering CRISPR-Cas9 mRNAs to excise HIV-1 proviral DNA. To this end, CCR5-targeted lipid nanoparticles (R5-LNPs) were formulated through microfluidic mixing. These nanoparticles demonstrated an mRNA encapsulation efficiency greater than 90%. The R5-LNPs were shown to be safe at doses of up to 10 µg/ millions of monocyte-derived macrophages (MDMs). The R5-LNPs showed a CCR5 receptor-mediated enhanced mRNA translation in MDMs than untargeted control LNPs (C-LNPs). Additionally, R5-LNPs successfully excised more than 90% of proviral DNA in the infected MDMs and CD4+ T-cells, which was higher than C-LNPs. Overall, this study demonstrates the potential of CCR5-targeted CRISPR-Cas9 delivery as a strategy for HIV-1 DNA elimination, providing a promising avenue toward a functional cure.

### 3 The Effect of Cannabinoids on Blood-Brain Barrier ABC Efflux Transporter Activity and Expression in the Context of HIV Antiretroviral Therapy

Alshakarchi, Z, MS^1^, Andrews, A, PhD^1^, Ramirez, S, PhD^1^; ^1^Department of Pathology, Immunology, & Laboratory Medicine, College of Medicine, University of Florida , Gainesville, FL, 32611 United States.

The blood brain barrier (BBB) not only limits entry of blood solutes and xenobiotics into the CNS parenchyma by virtue of its tight junctions but also by specialized efflux transmembrane proteins. This transport barrier is formed by the high expression of ATP binding cassette (ABC) efflux transporter proteins such as P-gp/ABCB1/MDR, MRP1/ABCC1 and BCRP/ABCG2/MXR. Efflux transporters in the BBB poses a major problem for drug delivery of therapeutics to the CNS, restricting 98% of small molecules and nearly all large therapeutic molecules from entering the brain. People living with HIV (PLWH) rely on the use of antiretroviral therapy (ART) for lifelong management of HIV infection. Most ART drugs do not readily enter the CNS unless loss of BBB integrity is present. Various conditions can cause a breach to the BBB, including neuroinflammation and use/misuse of controlled substances. Some ART medications have adverse effects on neuronal function which could manifest if BBB function is compromised. Here we tested whether commonly used cannabinoids such as cannabidiol and tetrahydrocannabinol often used by PLWH to manage pain, appetite, and inflammation impacted the transporter barrier. Experiments with primary human brain microvascular endothelial cells with and without cannabinoids were performed for evaluation of transporter gene expression. Our results showed significant effects on certain MDR genes but not others. Overall, these studies contribute to our understanding of how cannabinoids may modulate ABC transporters expression and function and impact ART CNS penetration.

### 4 Pancreatic Stellate Cells as A Novel Reservoir

Argeles, EA^1^, Cruz, PC, PhD^1^, Andrews, AA, PhD^1^, Ramirez, SR, PhD^1^; ^1^Department of Pathology, Immunology, & Laboratory Medicine, College of Medicine, University of Florida, Gainesville, FL, 32611 United States.

People with HIV (PWH) are living longer due to the effectiveness of anti-retroviral therapy. Although viremia can be managed, PWH are at greater risk of developing comorbidities including metabolic syndrome and diabetes. The pancreas is a critical endocrine organ, and little is known about HIV in the pancreas. Using RNASeq data through the Human Pancreas Analysis Program (HPAP), computational analysis was performed to detect the expression of HIV receptors (CD4, CXCR4 and CCR5) in 200,000 cells isolated from human islets. This analysis identified small subsets of cells expressing both CD4 and CXCR4. Further analysis revealed expression of both CD4 and CXCR4 in immune, endothelial and stellate cells. Stellate cells are a rare cell type that is understudied but can be found surrounding pancreatic ducts, acini and blood vessels. Immunohistochemistry of human pancreatic stellate cells in culture confirmed the expression of CD4 and CXCR4 but also unexpectedly CCR5. Further evidence of HIV receptor expression was gathered using flow cytometry and digital PCR. Stellate cells were then evaluated for HIV permissivity by infection with HIV strains (IIB, JR-FL 89.6) and immunostained for p24 at 3- and 6-days post infection. The results showed productive infection of stellate cells by HIV. Ongoing experiments will inform on cell inflammatory status, the rate of viral replication and viral latency. To our knowledge, these are the first findings that implicate stellate cells as a possible reservoir for HIV in the pancreas with implications for endocrine dysfunction in PWH. Supported by Internal UF Funds

### 5 Amyloid Beta-Induced Phase Separation of miRNA-Bound Ago2 to RNA Processing Bodies

Bhattacharyya, SN, PhD^1^, Mukherjee, K, PhD^2^; ^1^Dept. of Pharmacology and Experimental Neuroscience, University of Nebraska Medical Center, Omaha, NE, 68106 United States. ^2^Dept of Anesthesiology, University of Nebraska Medical Center, Omaha, NE, 68106 United States.

Phase separation into membrane-less organelles modulates protein activity in eukaryotic cells. miRNA-repressed mRNAs alongside Ago proteins congregate in RNA-processing bodies (P-bodies), subcellular structures formed by various RNA-binding and regulatory proteins within eukaryotic cells. Ago2, a crucial miRNA-binding protein, pairs with miRNAs to inhibit protein synthesis by attaching to mRNAs and directing them to P-bodies. Nonetheless, the mechanisms governing Ago2 and miRNA-repressed mRNA compartmentalization within P-bodies remain not entirely clear. We established a detergent-permeabilized cell-based assay system to examine the phase separation of externally introduced Ago2 into P-bodies in vitro. Our findings indicate that miRNA binding to Ago2 is crucial for its localization to P-bodies and that this process is ATP-dependent. Oscillations in osmolarity and salt concentration also influence Ago2 compartmentalization into P-bodies. Moreover, amyloid beta oligomers facilitate Ago2 recruitment to P-bodies by hindering cellular Ago2 dynamics and inhibiting mTORC1 activity. Conversely, the RNA-binding protein HuR interrupts P-body recruitment by “sponging” away Ago2-associated miRNAs. This is an novel mechanism of regulation of miRNA activity by amyloid protein in neuronal and non-neuronal cells. Supported by UNMC Start UP Fund and Lieberman Research Award Fund

### 6 HIV-1 Infection Accelerates Alzheimer’s disease pathology in humanized APP knock-in mice

Bhattarai, S, MS^1^, Yeapuri, P, PhD^1^, Foster, EG, BS^1^, Kadry, R, MS^1^, Lu, Y, BS^1^, Sapkota, R, BS^1^, Kumar, M, MS^1^, Mitra, A, PhD^2^, Pathak, H, PhD^2^, Dash, P, PhD^1^, Gorantla, S, PhD^1^, Poluektova, LY, MD, PhD^1^, Mosley, RL, PhD^1^, Gendelman, HE, MD^1^; ^1^Pharmacology and Experimental Neuroscience, University of Nebrask Medical Center , Omaha , NE, 68198 United States. ^2^Pathology and Laboratory Medicine , University of Kansas Medical Center , Kansas City , KS, 66160 United States.

The prevalence of age-associated Alzheimer’s-like disease is rising among people living with HIV-1 (PLWH). The absence of suitable animal models limits research into the simultaneous study of HIV and AD comorbidities. We developed a novel humanized AD mouse model susceptible to HIV-1 infection on an immunocompromised NOG background. We knocked in the Swedish mutation of the human amyloid precursor protein (APP) using CRISPR-Cas9 technology (APP-KI). To allow the development of human-like microglia in the brain, APP-KI mice were further crossed with transgenic NOG/hIL34 [NOG/Tg (CMV-IL34)], to develop NOG/APP-KI/IL-34 (NAIL) mice. Four-month-old, humanized NAIL mice were infected with HIV-1ADA strain and sacrificed 8 weeks post-infection. Plasma viral load and immunohistochemistry confirmed productive HIV-1 infection in the brain and periphery. ELISA quantification of amyloid-beta (Aβ42) reveled significantly increases amyloid load compared to uninfected controls. Spatial transcriptomics revealed that neurons were the most affected cell type during HIV infection, compared to glial cells. Notably, the top neuronal upstream regulator genes activated by HIV-1 infection in the AD context included APP, presenilin-1, microtubule-associated protein tau (MAPT), and mammalian target of rapamycin (mTOR). Additionally, synaptogenesis and GABAergic receptor signaling pathways emerged as key mechanisms driving the exacerbation of AD pathology in response to HIV infection. Altogether, we observed that HIV-1 infection accelerates AD pathogenesis in a novel humanized AD mouse model. Supported by NIH P01 DA028555, R01NS034239-28, R01 AG043540

### 7 HIV-1Tg Rats Exhibit Greater in Opioid Tolerance and Withdrawal

Bishir, M, MS^1^, Abdul Azeeze, MST, PhD^1^, Jawadi, SA^1^, Huang, W, MS^1^, Chidambaram, SB, PhD^2^, Sariyer, IK, PhD^3^, Chang, SL, PhD^1^; ^1^Institute of NeuroImmune Pharmacology, Seton Hall University, South Orange, NJ, 07079 United States. ^2^Department of Pharmacology, JSS College of Pharmacy, JSS Academy of Higher Education and Research, Mysuru, Karnataka, 570015 India. ^3^Department of Microbiology, Immunology, and Inflammation, Center for Neurovirology and Gene Editing, Temple University Lewis Katz School of Medicine, Philadelphia, PA, 19140 United States.

We previously reported greater incidence of opioid use among the people living with HIV (PLH), but the role of HIV-1 viral proteins in development of opioid tolerance and dependence in PLH remains unclear. Our research was the first to show an increase in mu opioid receptor expression in the presence of HIV viral proteins. We also found overlapping signaling pathways mediated by HIV-1 viral protein and morphine, resulting in increased opioid dependence in PLH. Using HIV-1Tg rats, model to mimic PLH on cART, we compared morphine tolerance and naloxone induced withdrawal between female F344 and HIV-1Tg rats. On Day 1, rats received 2 morphine (75 mg) pellets or placebo, followed by 4 pellets on Day 2 subcutaneously. Tail flick latency was recorded before pelleting, at 2- and 8-hours following pelleting on Days 1 and 2, and once daily on Days 3–7. On Day 7, naloxone (2 mg/kg, i.p.) was injected to induce withdrawal. Both groups given morphine showed increased latency within 2 hours of pelleting. However, by Day 3, HIV-1Tg rats showed a decrease in the latency, whereas F344 rats showed a decrease on Day 4. By Day 7, F344 rats returned to baseline latency, but HIV-1Tg rats failed to recover completely, suggesting greater tolerance in HIV-1Tg rats. Naloxone induced physical withdrawal symptoms including tooth chattering, paw tremor, diarrhea, squeaking and orbital tightening. Episodes of paw tremor were significantly higher in HIV-1Tg compared to F344 rats received morphine pellets. These findings reveal that HIV-1Tg rats develop greater morphine tolerance and withdrawal symptoms. Supported by NIH DA046258 to SLC and DA052284 to IKS and SLC

### 8 Piperazine based Lipid Nanoparticles facilitate enhanced endosomal escape and CRISPR-Cas9 mediated HIV-1 proviral DNA excision

Chaudhary, BN, MS^1^, Panja, S, PhD^1^, Ali, MU, BS^1^, Dey, SS, MS^1^, Patel, MK, PhD^1^, Zaman, LA, PhD^1^, Gendelman, HE, MD^1^; ^1^Pharmacology and Experimental Neuroscience, University of Nebraska Medical Center, Omaha, NE, 68198 United States.

Despite antiretroviral therapy (ART), the persistence of latent HIV-1 proviral DNA in CD4+ T cells remains a major obstacle to achieving a functional cure. Therefore, CRISPR-Cas9-mediated viral DNA excision is essential. To this end, our laboratory has developed compositionally unique lipid nanoparticles (LNPs) for CRISPR-Cas9/guide RNA (gRNA) delivery to HIV-1 reservoirs for viral elimination. The gRNAs target structurally conserved regions (tat, rev, and gp41) of the HIV-1 genome and excise them. However, the effectiveness of LNP-based CRISPR therapy is limited by insufficient endosomal escape and reduced mRNA translation efficiency. To address this issue, we synthesized a library of piperazine-based biodegradable ionizable lipids and evaluated their mRNA translation efficacy. Our lead ionizable lipid-based LNPs (Bpip-LNP) achieved significantly higher mRNA translation efficiency than LNPs formulated with the gold-standard ionizable lipids that are D-Lin-MC3-DMA and ALC-0315. In BALB/c mice, Bpip-LNPs demonstrated a 2-fold higher mRNA translation compared to MC3-LNPs. Furthermore, a single dose of Bpip-LNPs with an optimal CRISPR-Cas9 mRNA/gRNA ratio resulted in approximately 62% elimination of HIV-1 proviral DNA from the latently infected T-lymphocytic cell line. Overall, the piperazine head group in the ionizable lipid demonstrated an improved endosomal escape and enhanced CRISPR-Cas9 delivery to HIV-1 reservoir cells.

### 9 Impact of cannabidiol (CBD) on HIV infection and methamphetamine abuse associated neuroinflammation

Clemencia, Shanee, BS^1^, Fox, Howard S., MD, PhD^2^, Chand, Subhash, PhD^1^; ^1^Department of Anesthesiology, University of Nebraska Medical Center, Omaha, NE, 68198 United States. ^2^Department of Neurological Sciences, University of Nebraska Medical Center, Omaha, NE, 68198 United States.

Although there is an 8% decrease in HIV infection in the U.S., the prevalence of people with HIV (PWH) has increased due to effective combinational antiretroviral therapy (cART). PWH are prone to substance abuse such as methamphetamine (METH), opioids, cannabis, and alcohol. HIV and METH increase neuroinflammation, whereas cannabidiol (CBD), a component of cannabis, is known to attenuate inflammation; however, their collective impact is yet to be elucidated. Here, we sought to understand the impact of CBD on HIV infection and METH abuse-associated neuroinflammation. NLRP3 inflammasome assembly is one of the signaling pathways requiring pNF-kB, caspase-1, ASC, and NLRP3 genes to activate and function NLRP3 inflammasome. Iba-1 is a marker for activated microglia. We evaluated the effect of CBD in modulating NLRP3 inflammasome activation using monocyte-derived microglia (MDMi) and the U1 pro-monocytic cell line. U1 and MDMi were infected with HIV-1 and treated with METH (10 µM) and CBD (10 µM) over 24 hr. Our western blot analysis revealed that microglial activation markers such as Iba-1, TNF-a, and IFN-g were upregulated in the HIV-infected and METH-treated groups. NLRP3 inflammasome modulatory genes such as caspase-1, ASC, NLRP3, and pNF-kB were upregulated in HIV-infected and METH-treated MDMi compared to control. On the other hand, CBD treatment significantly downregulated these NLRP3 inflammasome proteins. Moreover, Iba-1 expression was reduced considerably after CBD treatment in the HIV-infected and METH-treated MDMi, indicating CBD can attenuate microglial activation. Supported by NIH/NIDA/R03DA060076

### 10 Identifying Asp316, Glu322, and Asp569 of human serotonin transporter as the recognition binding sites for their potential interactions with HIV-1 Tat protein and their effects on serotonin transport

DeLaney, J, BS^1^, Fengying, G, MD, PhD^1^, Jimenez Torres, A, PhD^1^, Chang-Guo , Z, PhD^2^, Zhu, J, MD, PhD^1^; ^1^Department of Drug Discovery and Biomedical Sciences, College of Pharmacy, University of South Carolina, Columbia, SC, 29208 United States. ^2^Department of Pharmaceutical Sciences, College of Pharmacy, University of Kentucky, Lexington , KY, 40503 United States.

The lifetime prevalence of depression in people living with HIV is twice as high as in the general population despite the widespread use of cART. Dysregulation of serotonin (5-HT) transmission has been implicated as a factor in the development of depression. We have demonstrated that HIV-1 Tat protein in vitro and in vivo inhibits reuptake of 5-HT via serotonin transporter (SERT). This study explored how human SERT (hSERT) interacts with Tat through the recognition binding sites and determined the mutational effects on 5-HT uptake via hSERT. Computational modeling revealed a direct hSERT-Tat complex and predicted Asp319, Glu322, and Asp569 as strong hydrogen bond with Tat. We determined the effects of the mutated hSERT residues on basal 5-HT uptake and Tat-inhibited SERT function in the hSERT mutants, N316A, E322A and N569A. Compared to WT hSERT, the IC50 values for 5-HT competing [3H]5-HT uptake showed no difference in N316A, E322A and N569A, while the IC50 values for cocaine or paroxetine (a selective SERT inhibitor) inhibiting [3H]5-HT uptake were significantly decreased in N316A but not in E322A and N569A. We also determined the IC50 values for cocaine or paroxetine inhibiting [3H]Citalopram (a selective SERT inhibitor) binding, which were increased in N316A but unchanged in E322A and N569A relative to WT hSERT. Ongoing project will test whether these mutants attenuate Tat-SERT interaction. These results provide mechanistic insights into developing allosteric modulators as a therapeutic strategy for attenuating Tat-induced dysregulation of serotonin transmission. Supported by NIH grants DA035714, DA047924 and DA057866

### 11 Fentanyl increases HIV infection/dissemination elevating CCR5 and neuroinflammation in a humanized mouse model of brain infection

Dutta, D, PhD^1^, Makarov, E, MS^1^, Thiele, M, BS^1^, Fernandes, A, BS^1^, Gorantla, S, PhD^1^; ^1^Pharmacology and Experimental Neuroscience, University of Nebraska Medical Center, Omaha, NE, 68198 United States.

Drug abuse exacerbates central nervous system (CNS) HIV infection and HIV-associated neurocognitive disorders (HAND). Fentanyl (Fent), a potent opioid, may increase HIV risk and impact CNS viral spread and HAND. We studied the effect of Fent on HIV infection dynamics and tissue/brain dissemination in a novel humanized glial mouse model reconstituted with human immune system and microglia. Mice received Fent (1 mg/kg/day) for one week before HIV-1ADA infection (2×10^5^ TCID_50_). Plasma HIV RNA was measured by ddPCR. Four weeks post-infection, using flow cytometry, immunophenotyping was done on blood, spleen, and bone marrow (BM). Viral RNA/DNA was measured in the spleen, lymph nodes (LN), BM, and brain. Fent significantly increased plasma HIV RNA (>3 log, p3 log, p< 0.05) in Fent-treated animals. Immunohistology confirmed CCR5 upregulation in the spleen with Fent. Magnetic resonance imaging and spectroscopy (MRI/MRS) revealed increased polyamines and decreased Nuclear Overhauser effect (NOE) with Fent, indicating neuroinflammation. A humanized mouse model of HIV brain infection demonstrated that Fent augments HIV infection as well as viral dissemination to the brain by elevating CCR5 and neuroinflammation. Thus, the study depicts the significant impact of Fent abuse on HIV pathogenesis. Supported by 5R01DA054535-04

### 12 Attenuation of parkinsonian pathologies by inhibition of Bach1 in the α-synuclein preformed fibril-induced mouse model of Parkinson’s disease.

Dutta, D, PhD^1^, Kaidery, NA, PhD^1^, Soni, P, PhD^1^, Muralidharan, P, MS^1^, Richardson, R, BS^1^, Chakraborty, S, PhD^4^, Tripathi, S, PhD^4^, Sokratian, A, PhD^5^, Viverette, E, PhD^5^, Attucks, OC, PhD^6^, Valcarce, C, PhD^6^, Matsumoto, M, PhD^7^, Igarashi, K, PhD^7^, Paul, B, PhD^4^, West, AB, PhD^5^, Sharma, S, PhD^3^, Thomas, B, PhD^2^; ^1^Darby Children’s Research Institute, Departments of Pediatrics, Medical University of South Carolina, Charleston, SC, 29425 United States. ^2^Darby Children’s Research Institute, Departments of Pediatrics, Neuroscience, Drug Discovery, Medical University of South Carolina, Charleston, SC, 29425 United States. ^3^Department of Biochemistry, Hollings Cancer Center, Medical University of South Carolina, Charleston, SC, 29425 United States. ^4^Department of Pharmacology, , Johns Hopkins University School of Medicine, Baltimore, MD, 21205 United States. ^5^Department of Pharmacology and Cancer Biology, Duke University, Durham, NC, 27710 United States. ^6^vTv Therapeutics LLC, vTv Therapeutics LLC, High Point, NC, 27265 United States. ^7^Department of Biochemistry, Tohoku University, Graduate School of Medicine, Sendai, Japan.

Nuclear-factor-erythroid 2-related factor 2 (Nrf2) is a key transcription factor orchestrating a multifaceted response to confer neuroprotection in Parkinson’s Disease (PD). Unfortunately, FDA-approved electrophilic Nrf2 activators cause irreversible alkylation of cysteine residues in various cellular proteins, resulting in undesirable side effects. Moreover, Nrf2 stabilization and its activation induce the expression of the transcriptional repressors of Nrf2 via a negative feedback mechanism. Expression of BTB and CNC homology 1 (Bach1), a known transcriptional repressor of the Nrf2 pathway, is upregulated in PD brains, suggesting that Bach1 inhibition might be neuroprotective. In this study, we established the mouse model of PD by injecting preformed fibrils (PFF) of α-synuclein into the striatum, and 6-months-post-injection documented phospho-α-synuclein pathology, markers of neuroinflammation, oxidative damage, dopaminergic neuronal loss in SNpc and motor impairments. Interestingly, genetic deletion of Bach1 and pharmacological inhibition by the newly identified non-electrophilic inhibitor (HPPE) significantly attenuated these pathological PD hallmarks in the PFF-injected PD mice. Further transcriptomic analysis of the differentially expressed genes common to Bach1-/- PFF-injected mice and HPPE-treated PFF-injected mice revealed that the complement and coagulation cascades were enriched in the PD mice that were markedly reduced by Bach1 deletion or inhibition. This study underscores the importance of Bach1-mediated complement cascades as a therapeutic target in PD.

Supported by NIH grant R01NS101967 and the Department of Defense grant HT94252310443.

### 13 Effects of cannabis on metabolic phenotype in monocyte derived macrophages from people living with HIV: Implications for HIV-related neuropathogenesis

Ford, Mary^1^, Avalos-Leyva, B, PhD^1^, Laird, A^1^, Crescini, M^1^, Iudicello , J, PhD^1^, Fields, J, PhD^1^; ^1^Department of Psychiatry, University of California, San Diego, La Jolla, CA, 92093 United States.

Despite antiretroviral therapies (ART), a high prevalence of neurocognitive impairment (NCI) persists among people with HIV (PWH). Immunometabolic mechanisms in brain macrophages may provide therapeutic targets to reduce brain inflammation in PWH. Cannabis use is associated with reduced NCI and inflammation of PWH, but effects on monocyte-derived macrophage (MDMs) immunometabolic phenotype are not fully understood. This study examines the impact of cannabis on metabolic activity in MDMs from PWH and people without HIV (PWoH). MDMs were generated from cannabis-using and cannabis-naïve PWH (n=4 and n=6, respectively) and PWoH (n=4 and n=6). MDMs were treated with delta-9-tetrahydrocannabinol (THC), cannabidiol (CBD) or vehicle for 1 hour, followed by IL-1β co-stimulation for 24 hours. Mitochondrial activity was measured using MitoTracker (MT). Glycolytic and mitochondrial MDM metabolism was assessed with Seahorse XF Mito Stress Test. Cannabis use was associated with higher MT intensity in MDMs of PWH incubated with IL-1β and THC compared to cannabis-naïve users. In MDMs of PWH incubated with IL-1β and THC, cannabis users demonstrated higher MT activity relative to naïve users. Additionally, cannabis use diminished IL-1β-induced increases in extracellular acidification rate (ECAR) and oxygen consumption rate (OCR), markedly in PWH. These findings suggest cannabis may modulate mitochondrial function in MDMs, potentially counteracting HIV-related metabolic dysregulation. Future research will explore the mechanistic pathways linking cannabis and cellular metabolism in PWH on ART.

Supported by NIH NIDA | Grant 5R01DA053052-02

### 14 APP and PS1 knock-in mouse models on an immunodeficient NOG background develop intraneuronal amyloid pathology, microgliosis and extensive neuronal loss

Foster, EG, BS^1^, Yeapuri, P, PhD^1^, Machhi, J, PhD^1^, Kadry, R, MS^1^, Bhattarai, S, MS^1^, Lu, Y, BS^1^, Sil, S, PhD^1^, Sapkota, R, BS^1^, Srivastava, S, BS^1^, Kumar, M, BS^1^, Ikezu, T, MD, PhD^2^, Poluektova, LY, MD, PhD^1^, Gendelman, HE, MD^1^, Mosley, RL, PhD^1^; ^1^Department of Pharmacology and Experimental Neuroscience, University of Nebraska Medical Center, Omaha, NE, 68198 United States. ^2^Department of Neuroscience, Mayo Clinic Florida, Jacksonville, FL, 32246 United States.

Transgenic mice overexpressing familial Alzheimer’s disease (AD) mutations (FAD) show non-physiological traits, and their immunocompetent backgrounds limit their use in AD immunotherapy research. Preclinical models that reflect human immune responses in AD are needed. Using CRISPR-Cas9, we developed single (NA) and double (NAPS) knock-in (KI) amyloid precursor protein (APP, Swedish) and presenilin 1 (PS1, M146V) mice on an immunodeficient NOG background. KI was confirmed by Sanger sequencing and mice were evaluated for AD-like pathology. Both NA and NAPS mice developed pathology without overexpression artifacts. Intraneuronal human Aβ deposits and amyloid-associated microgliosis were observed as early as 3 months and increased with age. Addition of the PS1 mutation doubled amyloid load. Notably, intraneuronal amyloid deposition resulted in amyloid associated microgliosis and broad neuronal loss, resulting in brain atrophy in aged mice. HSC human immune reconstitution of the mice showed the development of a human adaptive immune system but did not alter amyloid load at 6 months compared to non-humanized mice. These novel KI models replicate intraneuronal amyloid pathology, amyloid associated microgliosis and neuronal loss. Broad neuronal loss observed in these mice is a unique AD phenotype not generally replicated in transgenic or KI mouse models of AD. The human immune reconstitution potential of these mice, for the first time, enables novel studies of human immune responses and immunotherapies, in addition to the role of infections such as HIV in AD.

### 15 The Role of HIV Proteins in Psychostimulant Abuse

Galli, A, ^1^Department of Surgery, UAB, Birmingham, AL, 35233 United States.

The use of amphetamine (AMPH) has been linked to human immunodeficiency virus (HIV) transmission, as HIV can spread through heightened unprotected sexual activity that is associated with AMPH use disorder (AUD). For these reasons, AUD and HIV infection have been termed a double epidemic. In this relationship, the role played by AUD in HIV dependent neurodegeneration is well documented. Furthermore, in humans, HIV infection increases AMPH frequency of use, frequency and duration of binging, as well as amount. This is important since the DSM-5 criteria for addiction includes a progressive intensification in drug use (i.e. escalation). To date, no examples exist of verified mechanisms of how HIV/HIV proteins alter AMPH actions and/or behavioral expressions. AMPH behaviors stem from its ability to reverse the function of the DA transporter (DAT), causing non-vesicular DA release (NVDR), resulting in an increase in extracellular DA levels. To gain a deeper understanding of NVDR, we provided the first evidence that specific domains of human DAT (hDAT) engage in direct association with the plasma membrane lipid phosphatidylinositol 4,5-bisphosphate (PIP2). These interactions are essential for AMPH to promote NVDR and specific behaviors”. The HIV trans-activator of transcription regulatory protein Tat1-86 (Tat) is a protein that binds PIP2 with high affinity through its basic domains. Consistent with Tat high affinity for PIP2, we demonstrated that Tat impairs NVDR. We also demonstrated that Tat, by blunting AMPH-induced DA release (i.e. NVDR), promotes AMPH escalation.

### 16 Development of an Ultralong-Acting Dolutegravir Prodrug Homodimer

Bhoomika Gowda^1^, Suyash Deodhar^1^, Samiksha Raut^1^, Le Nam Thai Hoang^1^, Brandon Hanson^1^, Brady Sillman^1^, Brian P. Kearney^2^, Alborz Yazdi^2^, Howard E. Gendelman^1^, and Benson Edagwa^1^. ^1^Pharmacology and Experimental Neuroscience, University of Nebraska Medical Center, Omaha, NE, USA. ^2^Exavir Therapeutics, Inc., San Francisco, CA, USA.

Dolutegravir (DTG) is an effective integrase strand transfer inhibitor with a high barrier to resistance. It is a part of the recommended first-line treatment for adults and children living with HIV-1 infection. However, suboptimal adherence to the existing daily oral formulations of DTG limit its impact. To these ends, ultra-long-acting (ULA) formulations of DTG are at various stages of preclinical development. We have previously identified monomeric ester prodrug of DTG formulation (NM2DTG) with superior pharmacokinetic (PK) profiles compared to the native drug [Nat. Commun., 3226 (2022)]. To further improve NM2DTG’s PK and injection volume profiles, DTG prodrug homodimers were synthesized and screened. An ULA DTG injectable dimer prodrug formulation (NM7DTG) was then identified. NM7DTG formulation exhibited enhanced intracellular drug uptake, retention, and antiretroviral activities compared to parent DTG (NDTG). NDTG demonstrated a suboptimal PK profile at four months, with plasma DTG levels declining rapidly below 4x the PA-IC_90_ at four weeks. In contrast, a single dose of NM7DTG sustained therapeutic DTG levels above 4x PA-IC_90_ for > four months and was tolerated well. Loading more DTG molecules per prodrug unit mass in NM7DTG could potentially reduce drug injection volumes.

### 17 Bovine colostrum extracellular vesicles (EVs) administered to methyl-4-phenyl-1,2,3,6-tetrahydropyridine (MPTP) intoxicated mice demonstrate potent anti-inflammatory and neuroprotective activities.

Hollingsworth, DH, BS^1^, Srivastava, SS, BS^1^, Dey, SSD, MS^1^, Panja, SP, PhD^1^, Akter, SA, MS^1^, Kumar, MK, MS^1^, Du, XD, MS^1^, Saha, AS, BS^1^, Yeapuri, PY, PhD^1^, Bhattarai, SB, MS^1^, Foster, EF, BS^1^, Kadry, RK, MS^1^, Oludipe, DO, BS^1^, Ehrenkranz, EE^1^, Hu, GH, PhD^1^, Oehlerking, JO^2^, Swarts, KS^2^, Sil, SS, PhD^1^, Gendelman, HG, MD^1^; ^1^Department of Pharmacology and Experimental Neuroscience, University of Nebraska Medical Center, Omaha, NE, 68349 United States. ^2^Oehlerking Farm, Oehlerking Farm, Elmwood, NE, 68349 United States.

EV-treated MPTP-intoxicated animals reduce microglial activation and halt neurodegeneration. Pro-inflammatory cytokine, and chemokine expression are decreased in EV-treated MPTP mice. Concomitantly, adaptive immune responses with increased Treg numbers, in the blood and spleen, of EV-treated MPTP mice support the links between neuroinflammation and neuroprotection. These were associated with significant increases in the numbers of nigral dopaminergic neurons. The results, taken together, demonstrate therapeutic activities of colostrum EVs for Parkinson’s disease which will be discussed.

Supported by National Institute of Health

### 18 The HIV-1 Transgenic Rat Brain Exhibits Disrupted Myelin Lipids

Jeffries, MA^1^, Cruz-Berríos, M^2^, Singhal, A^1^, Romer, MA^1^, Jordan-Sciutto, KL^2^, Grinspan, JB^1^; ^1^Division of Neurology, Children’s Hospital of Philadelphia, Philadelphia, PA, 19104 United States. ^2^Department of Oral Medicine, University of Pennsylvania, Philadelphia, PA, 19104 United States.

Human immunodeficiency virus (HIV)-associated neurocognitive disorders affect 30–50% of people with HIV (PWH) and associate with white matter pathology. The noninfectious HIV-1 transgenic (Tg) rat has an altered transcriptome suggestive of deficient myelin, but no studies have examined mechanisms of myelination in this model. Myelin is lipid-rich, which is critical for its structure and function. Disrupted brain lipid metabolism results in myelin abnormalities and predicts cognitive decline in HIV. We hypothesized that HIV-1 disrupts glial lipid metabolism, impairing myelin integrity and function. First, we examined myelin proteins for changes that could indicate myelin pathology. Immunoblotting in striatum, cortex, hippocampus, and callosa indicated no changes in myelin proteins MBP, CNP, MAG, or MOG at 3 or 9 weeks in HIV-1 Tg brains. However, at 6 months we observed significantly increased myelin proteins in HIV-1 Tg brain regions, suggestive of excessive myelin or disrupted composition. Next, we examined lipid synthesis enzymes. Expression of FASN was significantly decreased in HIV-1 Tg cortex and striatum at 3 and 9 weeks. However, at 6 months we observed increased FASN in cortex and striatum, and increased ACC1 in striatum. Lipidomics on myelin extracted at 9 weeks or 6 months showed significant increases across lipid classes in HIV-1 Tg myelin, particularly phospholipids and sphingomyelin. If changes in myelin lipids impair myelin structure and function, lipid metabolism may be a therapeutic target to improve white matter integrity and cognitive function in PWH. Supported by F32 MH135724

### 19 Novel oligomeric amyloid-β targeting antibody therapy for Alzheimer’s disease

Kadry, R, MS^1^, Bhattarai, S, MS^1^, Foster, E, BS^1^, Lu, Y, BS^1^, Srivastava, Shefali , BS^1^, Mosley, R. Lee , PhD^1^, Gendelman, H, MD^1^, Yeapuri, P, MD^1^; ^1^Pharamcology and Experimental neuroscience , University of Nebraska Medical Center , Omaha, NE, 68198 United States.

Background: Alzheimer’s disease (AD) is the most common type of dementia. AD is characterized by deposition of misfolded Amyloid-beta protein (Aβ) which form toxic aggregates called Aβ-plaque. While the non-aggregated forms of Aβ are involved in regular brain function, the Aβ-plaque aggregates cause neuroinflammation in the brain, which leads to irreversible neuronal death and memory loss. Antibodies targeting the toxic aggregates of Aβ could help clear the plaques and improve brain function. Methods: We used molecular modeling to identify a unique epitope to the toxic oligomeric Aβ aggregates. The identified epitope sequence was used to develop an antibody which exclusively binds to the toxic forms of Aβ. Mice were immunized with the epitope, and splenocytes were isolated and fused with P3/NSI/1-AG4-1 mouse plasmacytoma cells. This generated immortalized hybridomas producing antibody. These hybridomas were screened for monoclonal Abs that preferentially binds to the oligomeric and fibrillar Aβ isoform by immunoprecipitation assays (IP) and Surface plasmon resonance (SPR). Results: Comprehensive characterization by utilizing IP and SPR was performed between our novel oAβ-mAb against Aducanumab and Lacanemab showed selective binding of our novel mAb to oAβ and Aβ fibrils over monomers. Further, we plan to test our novel antibody for plaque clearing efficacy in an AD mouse model. In addition, we will also evaluate the reduction in brain inflammation and improvement in memory function after antibody treatment.

### 20 Development of CXCR4 Targeted Polymeric Polyplexes for Liver Delivery of MicroRNA Therapeutics in Alcohol Associated Liver Disease Treatment

Kalpana, MA, MS^1^, Zhang, C, PhD^1^, McVicker, B, PhD^1^, Bennett, R, PhD^1^, Ding, L, PhD^1^, Siddhanta, K, MS^1^, Oupicky, D, PhD^1^; ^1^Genetics, Cell Biology and Anatomy, University of Nebraska Medical Center, Omaha, NE, 68198-5040 United States.

Alcohol-associated liver disease (AALD) is a major health concern with limited therapeutic modalities. The overexpressed CXCR4 on stellate cells (HSC) and microRNA-155 in Kupffer cells (KC), are the two key players in AALD. We developed a polymer-based microRNA delivery platform for liver delivery of RNAi therapeutics. We hypothesized that the CXCR4 antagonizing polymer used in this DDS, can efficiently deliver therapeutic anti-miR155 to KC and inhibit CXCR4 on HSC in parallel and reverse liver fibrosis. The polymer PAMD is synthesized from AMD3100 and is modified with cholesterol and PEG. The nucleic acid gel retardation and RibroGreen assay demonstrate that the polymer/copolymers bind to anti-miR155 via electrostatic interaction and form polyplexes at w/w ratios ≥1.5 with encapsulation efficiency >98%. DLS and TEM demonstrate that cholesterol modification of PAMD forms cationic and nearly spherical polyplexes suitable for entry through liver fenestrae. The capping effect of cholesterol demonstrates improved colloidal stability of polyplexes in PBS and stability at 4°C. Cholesterol or PEG conjugation demonstrates improved polyplex integrity in vivo when incubated in human blood plasma at 37°C or in increasing concentration of polyanion heparin. All polyplexes retain >95% cell viability in CTB assay and demonstrate RNA transfection efficiency in RT-PCR with the inhibition of miR155 expression in LPS-induced miR155 overexpressed and polyplex-treated cells. Overall, we developed the potential delivery platform for liver delivery of microRNA therapeutics in AALD treatment.

### 21 Physical Properties of Stress Granules and the Impact of HIV Tat on Stress Granules’ Dynamics

Korunova, E S^1^, Sikirzhytski, V, PhD, Sikirzhytskaya, A, PhD, Wyatt, M, PhD, Twiss, J, PhD, Vasquez, P, PhD^3^, Shtutman, M, PhD^1^; ^1^Drug Discovery and Biomedical Sciences, University of South Carolina, Columbia, SC, 29208 United States. ^2^Biological Sciences, University of South Carolina, Columbia, SC, 29208 United States. ^3^Department of Mathematics , University of South Carolina, Columbia, SC, 29208 United States.

Stress granules (SGs) are non-membrane-bound RNA-protein assemblies essential for stress responses to factors like temperature fluctuations, oxidative stress, and nutrient deprivation. Impaired SG formation is linked to cancer, neurodegeneration, and chronic stress. However, their dynamic nature makes characterizing SGs’ physical properties challenging. To explore SG structure and its effects on macromolecules, we employed 40-nm Genetically Encoded Nanoparticles (GEMs) for single-particle tracking (SPT) in U2OS cells. First, we characterized the cytoplasm’s microrheology in cells expressing GEMs by extracting diffusion coefficients and motion types. GEMs exhibited near-Brownian motion, consistent with a larger cytoskeletal mesh in U2OS cells compared to other cell lines. Next, using U2OS cells expressing G3BP1-mCherry, a core SG component, and GEMs, we conducted live imaging before and after SG formation. Ultra-fast SPT using resonance confocal scanning and TrueSight super-resolution revealed GEMs intercalating into SGs. Diffusion coefficients from SPT data showed SG formation reduced GEM mobility, with two behaviors observed: a) bouncing on SG boundaries and b) entering and diffusing within SGs, with the potential to exit. We found that Tat HIV treatment can influence SG formation. We aim to assess this effect by analyzing SG formation/disassembly rates and changes in spatial viscosity. These findings suggest SGs form a heterogeneous mesh, raising questions about how SG formation affects macromolecular mobility and enzymatic reactions. Supported by R21DA058586

### 22 Control of HIV-1 Accelerated Alzheimer’s Disease by Antiretroviral Drugs in a Novel “NAIL” Humanized Mouse

Kumar, MK, MS^1^, Du, XD, BS^1^, Zhang, CZ, BS^1^, Foster, EF, BS^1^, Bhattrai, SB, MS^1^, Yeapuri, PY, PhD^1^, Kadry, RK, MS^1^, Gorantla, SG, PhD^1^, Sil, S, PhD^1^, Gendelman, HE, MD^1^; ^1^Department of Pharmacology and Experimental Neuroscience, University of Nebraska Medical Center, Omaha, NE, 68131 United States.

Recent evidence supports the notion that neuroinflammation driven by HIV-1 infection and aging affects the onset and progression of Alzheimer’s disease (AD). However, the mechanisms underlying these effects and their control remain poorly understood. To address this, we developed an AD knock-in (KI) mouse model carrying human amyloid precursor protein (APP)KM670,671NL Swedish mutation. The resulting founder mice were crossed with immunodeficient NOG (NOD.Cg-PrkdcscidIl2rgtm1SugTg(CMV-IL-34)1/Jic) to generate NOGAPPKM670,671N/ IL-34 (NAIL) mouse, enabling studies of progressive HIV-1 infection in the setting of antiretroviral therapy (ART) and AD. The hypothesis rested that HIV-1 infection affects the crosstalk between innate and adaptive immunity in AD and control by ART restricts those responses. HIV-1-infected NAIL mice, reconstituted with human hematopoietic stem cells, were infected at six months with HIV-1ADA (a macrophage-tropic viral strain) to facilitate microglial infection. Following productive viral infection, animals were treated with emtricitabine, tenofovir, and dolutegravir combinations in the animal feed. ART induced a threefold reduction in plasma HIV-1 RNA and restored CD4+ and CD8+ T-cell counts. Notably, HIV-1-infected NAIL mice demonstrated little changes in soluble and insoluble amyloid-β levels in the cortex compared to uninfected mice. Immunofluorescence revealed limited microglial and astroglial reactions with a parallel senescence-associated phenotype. These findings highlight the complex interactions between HIV-1, ART, and AD pathology. Supported by This work was supported by the National Institutes of Health Grants P01 DA028555, R01 NS36126, P01 NS31492, and P01 MH64570

### 23 Novel drug delivery modality to suppress HIV neuropathogenesis

Kumar, Santosh, PhD^1^, Zhou, Lina, PhD^1^, Godse, Sandip, MS^1^, Sinha, Namita, BS^1^, Kochat, Harry, PhD^1^; ^1^Pharmaceutical Sciences, The University of Tennessee Health Science Center, Memphis, TN, 38163 United States.

Despite significant advances in HIV treatment, neuroHIV and HIV-associated neurocognitive disorder (HAND) remain persistent challenges. A major issue is that most antiretroviral regimens fail to achieve therapeutic concentrations in the brain, allowing HIV to continue within brain and cause neuroinflammation. Therefore, finding a novel drug delivery modality to treat neuroHIV and HAND is critically important. Our laboratory has been developing synthetic lipids and extracellular vesicle-based (EV-Lip) hybrid drug delivery system for antiretroviral drugs using intranasal route effectively and suppress HIV-associated neuropathogenesis. We have developed and characterized EV-Lip-based drug delivery system for the delivery of antiretroviral therapy (ART) drugs in brain. Our study has shown that an ART drug when encapsulated in EV-Lip system using intranasal delivery can significantly increase the brain drug concentration and reduce off-target effects in liver, plasma, and lungs in wild-type mice. Our findings also showed that EV-Lip-ART drug can suppress HIV-associated neuroinflammation, oxidative DNA damage, and neuronal damage. Finally, we have shown that the formulation significantly reduces HIV-associated motor and cognitive impairments in EcoHIV mice, and a nutraceutical adjuvant reduced HIV-associated neuroinflammation and cognitive impairments. We conclude that EV-Lip hybrid system using intranasal route could be a better alternative to treat HIV-associated neuropathology. Supported by NIH grants AG081140 and MH125670

### 24 Determining the recognition residues of human vesicular monoamine transporter2 (VMAT2) for their interactions with HIV-1 Tat protein, methamphetamine, and inhibitors and their effects on VMAT2-mediated dopamine transport

Laws, JL, MS^1^, Porter, KP, PhD^1^, Jiménez-Torres, AJ, MD, PhD^1^, Cirincione, AC, PhD^1^, Zhan, CZ, PhD^2^, Zhu, JZ, MD, PhD^1^; ^1^Drug Discovery and Biomedical Sciences, College of Pharmacy, University of South Carolina, Columbia, SC, 29208 United States. ^2^Department of Pharmaceutical Sciences, College of Pharmacy, University of Kentucky, Lexington, KY, 40508 United States.

Dopamine (DA) transporter (DAT) transports the extracellular DA into cytosolic space of the synaptic terminals, whereas the VMAT2 repackages the cytosolic DA into presynaptic vesicles for storage, which are critical for normal DA homeostasis. We have demonstrated a synergistic inhibitory effect of the combined methamphetamine (METH) and HIV-1 Tat protein on VMAT2-mediated DA uptake in inducible Tat transgenic mice. This study determined the recognition residues of human VMAT2 (hVMAT2) for their interactions with HIV-1 Tat protein. We used computational modeling and simulation to create initial hVMAT2-Tat binding mode, which reveals four favorable intermolecular hydrogen bonds with K41, S46, Y49, and E321 of hVMAT2. Through HEK-293T cells transfected these mutated hVMAT2, we determined whether the designed mutants attenuate Tat-inhibited VMAT2-mediated DA uptake. In a separate study, we optimized recombinant Tat1-86 (rTat1-86) concentration-dependently inhibits VMAT2-mediated DA uptake in isolated mouse whole brain vesicles with a 5.4 nM Ki value. Further, in vitro rTat1-86-induced a similar reduction of DA transport via DAT (29%) or VMAT2 (23%) in the isolated mouse whole brain vesicles, indicating that Tat may alter extracellular DA by inhibition of both DAT and VMAT2. Importantly, we found that in vitro applied 1 nM SRI-32743, a novel allosteric modulator or 10 nM GZ-793A, a non-competitive VMAT2 inhibitor, attenuated Tat-inhibited DA uptake via VMAT2. These findings impact the molecular mechanism of dysregulation of VMAT2-mediated DA transmission induced by Tat protein. Supported by This work was supported by NIH grants DA035714, DA047924 and DA057866

### 25 Peromyscus: establishing an innovative model for NeuroHIV

Li, HL, MD, PhD^1^, Mactutus, CFM, PhD^1^, Jaeger, CJ, PhD^2^, Sikirzhytskaya, AS, PhD^2^, Aksenova, MA, PhD^2^, Sikirzhytski, VS, PhD^2^, Hippocratis, KH, PhD^2^, Shtutman, MS, PhD^2^, Albrecht, HA, PhD^3^, Booze, RMB, PhD^1^; ^1^University of South Carolina/Cognitive and Neural Science Program, Psychology, Columbia, SC, 29208 United States. ^2^University of South Carolina/Department of Drug Discovery and Biomedical Sciences, College of Pharmacy, Columbia, SC, 29208 United States. ^3^University of South Carolina/Division of USC Immunology Center, School of Medicine/Internal Medicine, Columbia, SC, 29208 United States.

HIV-associated neurocognitive disorders have become clinical concerns in the older HIV-1 seropositive population. Traditional models of rodent HIV infection may not fully represent the genetic and phenotypic diversity characteristics in human populations. Peromyscus (aka deer mice) diverged over 25 million years ago from mice and rats and thus represents a distinct and ancient genetic lineage. In the current study, we developed a new, non-traditional model for NeuroHIV based on Peromyscus through EcoHIV infusion. First, in vitro, primary cortical cells treated with mScarlet-labeled EcoHIV for 48 hours, showed microglial EcoHIV expression. Second, in vivo, Peromyscus injected with EcoHIV-EGFP presented with a dramatic distribution of EcoHIV in the prefrontal cortex area after 2 weeks and combined with Iba1 staining further established microglial EcoHIV infection in Peromyscus. Next, we observed synaptodendritic alterations (pyramidal neurons from layers II-III of medial prefrontal cortex) through DiOlistic labeling technique. Significant alterations in neuronal dendritic arbors were found in the mPFC pyramidal neurons of Peromyscus following EcoHIV infection, relative to control. The complexity and extent of dendritic arbor alterations were indicated by a shift in dendrites closer to the cell body and loss of dendrites extending to more distal connections in the EcoHIV-infected Peromyscus. Our data suggest that Peromyscus could be a valuable model for studying HIV-1 infection dynamics in producing NeuroHIV. Supported by AG082539, DA059310, MH106392, NS100624

### 26 Serum and CSF Cytokine Profiles Linked to Neuropsychiatric Symptoms and Brain Diffusivity in Long COVID

Liang, H, Ryan, MR^2^, Ernst, T^1^, Oishi, K^3^, Cunningham, EC^1^, Kottilil, S^4^, Chang, L^1,4^; ^1^Diagnostic Radiology and Nuclear Medicine, University of Maryland School of Medicine, Baltimroe, MD, 21201 United States. ^2^Program in Neuroscience, University of Maryland School of Medicine, Baltimore, MD, 21201 United States. ^3^Department of Radiology, Johns Hopkins University School of Medicine, Baltimore, MD, 21218 United States. ^4^Institute of Human Virology, Department of Medicine, Division of Infectious Disease, University of Maryland School of Medicine, Baltimore, MD, 21201 United States.

Background The relationship between immune dysfunction and neuropsychiatric symptoms in long COVID remains unclear. This study extends on our previous findings of persistent neuropsychiatric symptoms and abnormal brain diffusivities in long COVID by evaluating possible relationship with serum and CSF inflammatory markers. Methods We compared 30 long COVID participants (10 men, ∼6 months post COVID) to 27 never-COVID controls (11 men), analyzing cytokine/chemokine markers (IL2, IL4, IL6, IL8, IL10, IL13, CCL11, IP10) in serum and CSF. Marker levels were correlated with neuropsychiatric symptoms (NIH Toolbox® and PROMIS®) and abnormal brain diffusivities (diffusion tensor imaging). Results Compared to controls, long COVID participants had lower IL10 (p=0.023) and trends for lower CCL11 and IL2 in plasma, but similar CSF marker levels. Older age correlated with higher CSF IL8, CCL11, and IL10 levels (p<0.001–0.02). Lower plasma IL2 correlated with greater anger (r=-0.36, p=0.017), negative affect (r=-0.3, p=0.05), and lower life satisfaction (r=0.4, p=0.007). Lower CSF IL10 correlated with greater pain intensity (r=-0.38, p=0.048) and lower self-efficacy (r=0.44, p=0.02). Lower plasma IL6 correlated with higher fractional anisotropy (r=−0.31, p=0.037) but lower radial diffusivity (r=0.34, p=0.031) in the right superior longitudinal fasciculus. Lower CSF CCL11 also correlated with higher mean diffusivity in left amygdala (r=−0.48, p=0.024). Conclusions Cytokine deficiencies, particularly IL2 and IL10, may contribute to persistent neuropsychiatric or pain symptoms in long COVID. Supported by NIH grant R21-NS121615

### 27 Methamphetamine enhancement of gp120-induced membrane hyperpolarization via NMDAR-mediated K+ efflux

Liu, J, MD, PhD^1^, Xia, JX, PhD^1^, Xiong, H, MD, PhD^1^; ^1^University of Nebraska Medical Center, College of Medicine, Omaha, NE, 68198-5880 United States.

Methamphetamine (Meth) abuse exacerbates neurocognitive deficits in infected individuals and gp120 transgenic animals. The underlying mechanisms are not fully understood. The continually evolving role of NMDA receptors (NMDARs) in the pathogenesis of neurocognitive deficits has become increasingly evident. Gp120 and Meth have been shown to interact with NMDARs resulting in neuronal dysfunction and injury. Accumulating evidence suggests that NMDARs mediate K+ efflux and neuronal apoptosis. The aim of this study was to investigate if Meth enhances gp120-induced neuronal dysfunction by hyperpolarizing neuronal cell membrane via NMDAR-mediated K+ efflux. Experiments were conducted on hippocampal brain slices prepared from SD rats. Membrane potential was recorded on the CA1 neurons using conventional patch-clamping technique. Bath application of Meth (10µM) and gp120 (200pM) each alone had no significant effects on resting membrane potential. In contrast, Meth enhanced gp120-associated membrane hyperpolarization and suppressed spontaneous firing when applied in combination. The Meth/gp120-mediated membrane hyperpolarization was blocked by bath application of either specific NMDAR antagonists (MK-801, APV) or by BD1047, a Sigma-1 receptor antagonist. These results demonstrated that Meth enhanced gp120-associated membrane hyperpolarization via increasing NMDAR-mediated K+ efflux, leading to neuronal membrane hyperpolarization, which may underlie Meth exacerbation of neurocognitive deficits observed clinically in HIV-1-infected patients.Supported by NIH NIDA R01DA050540

### 28 Developing broadly neutralizing antibody-producing glial progenitors to eradicate Human immunodeficiency virus from the brain.

McDougall, Lorissa, MS^1^, Walczak, P, MD, PhD^1^, Janowski, M, MD, PhD^1^, Chang, L, MD,^1,2^, Heredia, A, PhD^2^, Chu C, MD^1^, Alavi, A, MD^2^, Mohammad, S, PhD^2^, Liang, Y MB, PhD^1^, Atkinson, B, PhD^2^, Kalkowski, L, PhD^1^; ^1^Diagnostic Radiology and Nuclear Medicine, University of Maryland, Baltimore, Baltimore, MD, 21201 United States. ^2^Institute of Human Virology, University of Maryland, Baltimore, MD, 21201 United States.

Antiretroviral therapies (ART) are effective in suppressing viral replication; however, due to their poor penetrance across the blood-brain barrier, clearance of HIV from the brain is limited. Thus, to reduce resurgence of virus in the body, people with HIV require lifetime ART, which may have side effects that damage the liver, kidney and GI tract. Therefore, exploring new therapeutic strategies to eliminate HIV from the brain is urgently needed. This study takes advantage of the intrinsic white matter repair properties of glial progenitor cells (GRPs) in repairing HIV-induced neurological damage, and broadens the therapeutic capacity via genetic engineering, by producing broadly neutralizing antibodies (bNAb) for viral clearance through binding HIV glycoprotein gp120. Integration of the lentiviral bNAb constructs (CII and CIII) into the GRP genome was verified by flow cytometry (GFP reporter) and bNAb staining co-expression in 91.6% of the CII-bNAb-mGRPs and 89.9% of the CIII-bNAb-mGRPs. To measure functionality, a neutralization assay of the purified bNAbs showed >50% viral neutralization by 1.2 ug/mL of CII-bNAb and 0.4 ug/mL of CIII-bNAb. Lastly, in vivo intracerebral injection of the GRPs alongside gp120 expressing RAJI cells found that CII-bNAb bound 61.7% of cells while CIII-bNAb bound 21.6%. To achieve global brain targeting, we used MRI-guided intra-arterial delivery of GRPs to identify cell distribution in the brain. Ongoing experiments will indicate if this strategy allows for clearance of the viral reservoir in the brain. Supported by Grant Support from NIDA/NIH (R01DA056739; MPI: Walczak, Heredia)

### 29 DMF ameliorates deficits of cognition, mood, and opioid abuse in mice exposed to HIV-1-Tat protein and/or morphine.

McLaughlin, JP, PhD^1^, Hammond, HR, BS^1^, Eans, SO, MS^1^, Augustin, AM^1^, Cirino, TJ, PhD^1^, Thangavel, S, PhD^2^, Kaufman, MJ, PhD^3^; ^1^Dept. of Pharmacodynamics, University of Florida, Gainesville, FL, 32610 United States. ^2^Pharmaceutical Sciences, Texas A&M University, College Station, TX, 77845 United States. ^3^McLean Imaging Center, McLean Hospital/Harvard University, Belmont, MA, 02478 United States.

We hypothesize that conditional expression of the HIV-1 protein transactivator of transcription (Tat) and exposure to opioids in animal models synergistically induces changes analogous to HAND, including deficits in neurophysiology (increased reactive oxygen species (ROS), and reduced Brain-Derived Neurotropic Factor (BDNF) and mitochondrial biogenesis) and behavior (increased drug-seeking and depression-like behaviors, while impairing learning and memory performance). Testing this, we used the iTat-tg mouse model and Tat-null control mice to demonstrate that a 7-day expression of Tat protein increases morphine-seeking behavior, with a 2.5-fold potentiation of morphine conditioned place preference and increased morphine consumption in a two-bottle choice (TBC) assay. Moreover, exposure to morphine and Tat, alone and together, produce depression-like increased immobility in a tail suspension test and reduced voluntary consumption of saccharine in a TBC assay, and further reduced novel object recognition to demonstrate impaired learning and memory performance. Notably, oral administration of Dimethyl Fumarate (DMF) dose-dependently ameliorated these effects to varying degrees in mice exposed to Tat protein and/or morphine. Brains isolated from studied mice were used to assess expression of NFE2L2, ROS, BDNF and mitochondrial function and biogenesis. Collectively, our findings may suggest new mechanistic insights into the dysfunction induced by exposure to HIV-Tat protein and opioids, while demonstrating that DMF might benefit HIV-1 and opioid use disorder patients. Supported by R01- DA055568 from NIH/NIDA

### 30 Decoding Neural Mechanisms in Depression Associated with HIV-1 and Opioid Use

Moidunny, S, PhD^1^, Amerzhanov, D, PhD^3^, Bartmanski, BJ, PhD^3^, Tao, J, PhD^1^, Volmar, CH, PhD^2^, Kogadeeva, MZ, PhD^3^, Beurel, E, PhD^2^, Roy, S, PhD^1^; ^1^Department of Surgery, University of Miami School of Medicine, Miami, FL, 33136 United States. ^2^Department of Psychiatry and Behavioral Sciences, University of Miami School of Medicine, Miami, FL, 33136 United States. ^3^Genome Biology Unit, European Molecular Biology Laboratory, Heidelberg, Heidelberg, 69117 Germany.

Major Depression is prevalent in HIV-patients using opioids. Depression in HIV-patients is associated with higher mortality rates and poor adherence to antiretroviral therapy. Recent studies indicate that opioid use exacerbates HIV-induced neuronal damage and neurocognitive impairments; however, the neural mechanisms underlying depression associated with HIV-1 and opioid use are largely unknown. To address this gap in knowledge, we systematically investigated the effects of opioid treatment on development of anxiety-like and depression-like behaviors in wild-type and HIV-1 transgenic mice (Tg26). Mice were chronically treated with morphine, spontaneously withdrawn and subjected to a battery of behavioral tests: open-field test, sociability test and tail suspension test. At the end of behavioral testing, mice were humanely euthanized and brain tissues collected for RNA seq analysis. We observed that spontaneous and protracted morphine withdrawal induces anxiety-like and depression-like behaviors more severely in Tg26 mice, compared to wild-type controls. Consistently, we observed significant transcriptional changes in pathways and neural circuits associated with mood disorders, including neuronal synaptic transmission (glutamatergic and GABAergic), serotonergic and dopaminergic systems, mitochondrial dysfunction and glial activation. In summary, this study deepens our understanding of the neural mechanisms involved and provides valuable insights that could guide the development of therapies for anxiety and depression in HIV-patients in the future. Supported by The National Institute on Drug Abuse (NIDA), Miami Center for AIDS Research (CFAR)

### 31 Development of an In Vitro Assay to Follow miRNA Extracellular Export

Mukherjee, K, PhD^1^, Bhattacharyya, SN, PhD^2^; ^1^Dept of Anesthesiology, University of Nebraska Medical Center, Omaha, NE, 68106 United States. ^2^Dept of Pharmacology and Experimental Neuroscience, University of Nebraska Medical Center, Omaha, NE, 68106 United States.

The exchange of miRNA cargos mediated by extracellular vesicles among various mammalian cells is a key mechanism for controlling cellular miRNA levels and activity, thereby regulating the expression of miRNA-target genes in donor and recipient cells. Despite considerable excitement surrounding extracellular vesicle-associated miRNAs as potential biomarkers or therapeutic agents, the mechanism of selective packaging of miRNAs into endosomes and multivesicular bodies for subsequent extracellular export remains an underexplored area due to the lack of an assay system for studying such processes in vitro. We have developed an in vitro assay using endosomes isolated from mammalian macrophage cells to investigate miRNA packaging into endocytic organelles. The synthetic miRNAs employed in the assay are imported into the isolated endosomes during the in vitro reaction and become protected from RNase in a time- and concentration-dependent manner. The selective accumulation of miRNAs within endosomes requires both ATP and GTP hydrolysis and the miRNA-binding protein HuR. The HuR-miRNA complex binds to and activates the endosomal RalA GTPase, facilitating the import of miRNAs into endosomes and their subsequent export as part of extracellular vesicles. We have explored the importance of amyloid beta oligomers in regulating miRNA export using this unique assay system. Supported by Lieberman Research Award Fund (KM) and UNMC Start UP Fund (SNB)

### 32 Chandipura Virus Epidemiology: Addressing an Emerging Arboviral Encephalitis Syndrome in India

Parida, Mr, BS^1^; ^1^Department of Biological Sciences, Indian Institute of Science Education and Research Bhopal, Bhopal, Madhya Pradesh, 462066 India.

Chandipura virus (CHPV), an emerging arbovirus, has gained increasing attention in India due to its role in sporadic outbreaks of acute encephalitis syndrome (AES), predominantly affecting children. Characterized by a high fatality rate and rapid clinical progression, CHPV poses significant public health challenges in endemic regions. Despite its discovery in the 1960s, the epidemiology of CHPV remains underexplored, with limited surveillance systems and diagnostic tools hindering early detection and response efforts. This study aims to elucidate the epidemiological trends, transmission dynamics, and ecological factors contributing to the emergence of CHPV in India. It explores the interplay of vector biology, climate change, and socio-environmental determinants in driving outbreaks. Special attention is given to the vulnerability of pediatric populations, whose immature immune systems exacerbate disease severity. Furthermore, the paper discusses diagnostic and therapeutic gaps, highlighting the urgent need for innovative vaccine development and integrated vector management strategies. By addressing the epidemiological challenges of CHPV, this research underscores the importance of strengthening public health infrastructure and fostering regional and global collaborations to mitigate the impact of this emerging encephalitis syndrome in India.

### 33 Use of Biocompatible Ionic Liquid-coated Nanoparticles to Deliver ART to the HIV Reservoir within the CNS

Paris, JJ, PhD^1^, Mahdi, F, MS^1^, Hamadani, CM, MS^2^, Hu, D, PhD^2^, Vashisth, P, MS^2^, Jayalath, I, BS^2^, Sundaresan, V, PhD^2^, Braun, S, PhD^3^, Traina-Dorge, V, PhD^3^, Tanner, EEL, PhD^2^; ^1^Department of BioMolecular Sciences, University of Mississippi, University, MS, 38677 United States. ^2^Department of Chemistry and Biochemistry, University of Mississippi, University, MS, 38677 United States. ^3^Tulane National Primate Research Center, Tulane University, Covington, LA, 70433 United States.

Despite the success of antiretroviral therapeutics (ARTs), HIV cannot be eradicated from reservoirs within the body, particularly those in the central nervous system (CNS). We have used biocompatible ionic liquids (ILs), molten salts comprised of asymmetric cations and anions, that can ‘tune’ the affinity of nanoparticles to different cell types. We have developed two ILs with affinity for erythrocytes and microglia that promote nanoparticle ‘hitchhiking’ on blood components for delivery to the brain of inoculated animals and with cell-selective targeting of microglia. Preliminary data in rats demonstrate ∼48% of injected nanoparticles accumulating in the brain within 6 h. Nanoparticles were colocalized in 47% of CD11b(+) microglia. Further, we have loaded IL-nanoparticles with ART (abacavir, dolutegravir, lamivudine) and demonstrate retained antiviremic efficacy when IL-nanoparticles were administered to HIV-infected human peripheral blood mononuclear cells, an infected microglial cell line, or infected primary human microglia. We hypothesize that we can further improve the tunable profile of our IL formulation to target additional cell types (including astrocytes) and optimize cargo delivery for full coverage of the brain reservoir. To assist in the latter, we have created nanogold-carrying nanoparticles and have demonstrated their accumulation in primary human microglia. We anticipate that this cargo delivery strategy will be safe and efficacious when translated to Ecotropic HIV-infected rodents and SIV-infected macaques.

Supported by NIDA R01DA056875

### 34 Ethanol Enhances HBV Replication by Dampening Innate Immunity

Pathania, ASP, PhD^1^, Osna, NO, MD, PhD^1^, Poluektova, LYP, MD, PhD^1^; ^1^Pharmacology and Experimental Neuroscience, University of Nebraska Medical Center, Omaha, NE, 68106 United States.

Chronic HBV infection can lead to severe liver disease, worsened by alcohol consumption. Our study explores how ethanol affects HBV replication and innate immune responses, shedding light on this complex interplay. We used cell culture models (HepAD38, HepG2.2.15) and primary human hepatocytes (PHH) to investigate ethanol effects. To simulate ethanol metabolism, we utilized an acetaldehyde-generating system (AGS) we have reported before. HBV replication was analyzed via viral RNA, DNA, and cccDNA levels. Innate immune responses were assessed by evaluating key signaling molecules (RIG-I, MAVS, cGAS, STING, IRF3) and interferon production. AGS exposure in HepAD38, HepG2.2.15 cells significantly increased HBV RNA, DNA, and cccDNA levels while suppressing STAT1 phosphorylation and antiviral proteins APOBEC3B and 3G. Similar effects were observed in HBV-infected PHH treated with ethanol, which elevated HBV RNA, DNA, and cccDNA levels. Ethanol and HBV infection downregulated RIG-I, MAVS, and cGAS expression in PHH and AGS-treated HepAD38 and HepG2.2.15 cells, disrupting key antiviral pathways. Furthermore, ethanol reduced IRF3 phosphorylation, impairing poly(I:C)-induced activation and IFN-β production in these cells. These findings suggest that ethanol metabolism promotes HBV replication by suppressing the innate immune response, including interferon-stimulated genes (ISGs), and by disrupting RIG-I/MAVS signaling leading to decreased IFNβ production. These findings highlight the detrimental impact of alcohol consumption on HBV infection. Supported by NIH P50AA030407-5129

### 35 An Ultra-long-acting Tenofovir Prodrug Nanosuspension for HBV Treatment

Samiksha S. Raut^1^, Srijanee Das^1^, Grace Bybee^1^, Haritha Chava^1^, Mojisola Ogunnaike^1^, Weimin Wang^1^, Anup Pathania^1^, Nam Thai Hoang Le^1^, Howard E. Gendelman^1^, Larisa Poluektova*^1^, Natalia Osna*^1^, Benson Edagwa*^1^ ^1^University of Nebraska Medical Center, Omaha, NE, 68198, USA

Treatment of hepatitis B (CHBV) infection requires adherence to lifelong daily antiviral therapies. These include oral tenofovir (TFV) prodrugs [TFV disoproxil-/TFV alafenamide-fumarate – TDF/TAF] or entecavir. Treatment discontinuation leads to viral rebound and disease progression. This underscores the need for long-acting formulations to improve treatment outcomes. Considering these needs, we developed a scalable amino acid-free hydrophobic and lipophilic crystalline phosphonate TFV prodrug (M5TFV). Surfactant-stabilized aqueous nanosuspensions (NM5TFV) were produced at a >300 mg/ml drug concentration and evaluated for efficacy in transgenic mice (Tg05). NM1TFV, an injectable TFV prodrug shown to be longer acting than TAF (Sci Adv, 2023), was used as a control. Notably, a single intramuscular injection of NM5TFV at either 200 or 400 mg/kg TFV equivalents sustained HBV DNA suppression by >2.5 log 10 reduction over two months. Similar observations were recorded for NM1TFV dosed at 200 mg/kg. However, unlike NM5TFV, NM1TFV induced the expression of APOBEG3G and activation of inflammasome markers, including NLRP3 and AIM2, and IL1β gene, in livers of transgenic mice, suggesting divergent antiviral mechanisms of action. These results were further supported by in vitro data in HepAD38 cells. Additionally, NM5TFV was well tolerated at the injection site. These promising findings support further development of NM5TFV as an ultra-long-acting formulation candidate.

### 36 Role of organellar stress responses in BACE-1 inhibitor drugs-induced neurotoxicity

Quansah, Darius N.K, MS^1^, Geiger , Jonathan , PhD^1^; ^1^Department of Biomedical Science , University of North Dakota , Grand Forks , ND, 58203 United States.

Alzheimer’s disease (AD) is pathologically characterized by extracellular plaques composed of amyloid beta (Aβ) proteins that are produced by amyloidogenic cleavage of Aβ-precursor protein (AβPP), a process controlled by the rate-limiting beta-site AβPP cleaving enzyme (BACE-1). BACE-1 inhibitor drugs significantly reduce Aβ levels but have neurotoxic effects by unclear mechanisms. BACE-1 resides in endolysosomes that contain sufficiently large stores to ferrous iron (Fe2+) to cause insult-induced increases in intracellular Fe2+, reactive oxygen species (ROS) and cell death. We tested here the extent to which BACE-1 inhibitors cause neurotoxicity by perturbing endolysosome stores of Fe2+. Using SH-SY5Y human neuroblastoma cells, we determined the effects of clinically relevant concentrations of three BACE-1 inhibitors (elenbecestat, lanabecestat and verubecestat) and two non-BACE-1 anti-AD drugs (memantine and galanthamine) on endolysosome and mitochondrial stress responses. The BACE-1 inhibitor drugs but not the non-BACE-1 anti-AD drugs de-acidified endolysosomes, decreased endolysosome Fe2+ levels, increased cytosolic Fe2+ and ROS levels, increased mitochondrial Fe2+ and ROS levels, and caused mitochondrial membrane depolarization. The neurotoxic effects of BACE-1 inhibitor drugs were blocked by the endocytosed endolysosome Fe2+ chelator deferoxamine. These findings emphasize the importance of endolysosome Fe2+ in the development of new therapeutics against AD. Supported by Research Funding: 2R01DA032444, P20GM139759, RO1NS065957

### 37 Evaluation of Brain SIV reservoirs for Targeting with a CAR/CXCR5 T-cell Immunotherapy in Rhesus macaque Model of HIV Persistence

Sempek, ES, MS^1^, Acharya, A, PhD^1^, Finholm, IW, PhD^2^, Berg, JM, MS^2^, Reynolds, M, PhD^3^, Yeung, ST, PhD^4^, Dudley, DM, PhD^5^, Becklin, K, PhD^2^, Pampusch, MS, PhD^2^, Ndhlovu, LC, MD, PhD^4^, Skinner, PJ, PhD^2^, Byrareddy, SN, PhD^1^; ^1^Department of Pharmacology and Experimental Neuroscience, University of Nebraska Medical Center, Omaha, NE, 68198 United States. ^2^Department of Veterinary and Biomedical Sciences , University of Minnesota , St. Paul, MN, 55455 United States. ^3^Department of Pathobiological Sciences, University of Wisconsin – Madison, Madison, WI, 53706 United States. ^4^Division of Infectious Diseases, Weill Cornell Medicine, New York, NY, 10065 United States. ^5^Wisconsin National Primate Research Center, University of Wisconsin – Madison, Madison, WI, 53715 United States.

Persistent latent HIV reservoirs in the central nervous system (CNS) hinder a functional cure, contributing to viral rebound and HIV-associated brain damage. Targeting these reservoirs is critical for durable remission. Chimeric antigen receptor (CAR) T cells offer a promising immunotherapy by directing effector cells to infected targets. In this study, we are utilizing autologous T cells engineered to express the SIV-specific CD4-MBL-CAR targeting the infected cells and CXCR5, a follicular homing molecule that will drive the effector T cells to the lymphoid follicles and potentially to the CNS, to eliminate viral reservoirs. In our initial studies, Rhesus macaques (RMs) underwent CD8 depletion to establish a robust CNS infection. Next, SIV-specific CD4-MBL-CAR/CXCR5 T cells were infused into CD8 depleted, chronically SIV infected, ART treated RMs with ART suppressed to control viral replication. Following the infusion, the RMs underwent an analytical treatment interruption (ATI) to assess the capability of the CAR-T based therapy in eliminating the viral reservoirs and in delaying viral rebound. Plasma viral load was monitored for 14 days for acute study and plans to monitor 4 months for chronic study. Brain tissues at necropsy were analyzed for SIV DNA/RNA. Control RMs without infusion showed SIV+ DNA/RNA in all major CNS sanctuaries 14 days post-ATI. These initial findings characterize the source of SIV viral rebound in the CNS and will allow for testing the efficacy of CAR/CXCR5 T cells for long-term remission of HIV/SIV without ART in our ongoing studies.

### 38 Targeting Neuroinflammation in HAND: The Dual Role of Itaconate in HIV and Cocaine-Induced Toxicity

Sikirzhytskaya, A, PhD^1^, Aksenova, M, PhD^1^, Li, H, PhD^2^, Cui, BC, PhD^1^, Odhiambo, D, MS^1^, Korunova, E, MS^1^, Sikirzhytski, V, PhD^1^, Ji, H, MS^1^, Wyatt, MD, PhD^1^, Frizzell, N, PhD^3^, Booze, R, PhD^2^, Shtutman, M, PhD^1^; ^1^College of Pharmacy, University of South Carolina, Columbia, SC, 29208 United States. ^2^Department of Psychology, University of South Carolina, Columbia, SC, 29208 United States. ^3^School of Medicine, University of South Carolina, Columbia, SC, 29208 United States.

HIV-associated neurological disorder (HAND) is a debilitating complication of HIV infection, significantly worsened by substance abuse, particularly cocaine use disorder (CUD). HAND manifests as cognitive, motor, and behavioral impairments that severely impact the quality of life. CUD further exacerbates HAND by promoting neuroinflammation and neurodegeneration, leading to worsened neurological outcomes. This study investigated the role of the mitochondrial metabolite itaconate and its synthesis enzyme, aconitate decarboxylase 1 (Acod1), in the context of HIV-induced neuroinflammation. Itaconate plays a dual role in HIV-associated neuroinflammation. HIV proteins upregulate Acod1, which helps protect microglia from HIV- and cocaine-induced toxicity. Treatment with itaconate, 4-octyl-itaconate (4OI), protects neurons from the combined toxicity of HIV and cocaine. 4OI treatment mitigates HIV-induced neuroinflammation by enhancing microglial survival, restoring their ramified morphology indicative of a homeostatic state, and suppressing the release of inflammatory cytokines. Mechanistic studies show that 4OI activates Nrf2 antioxidant pathways to counter oxidative stress while downregulating genes linked to cytoskeletal remodeling and inflammatory microglia. Acod1 inhibition disrupts HIV-induced microglia, whereas 4OI suppresses HIV and cocaine-induced neuroinflammation. These findings highlight the therapeutic potential of the itaconate pathway in HAND, offering a promising avenue for developing novel treatments for HAND and its exacerbation by substance abuse. Supported by NIH NIDA R21DA058586

### 39 Epigenetic Regulation of NLRP6 Inflammasome-Mediated Neuroinflammation in HIV Tat and Ethanol-Exposed Astrocytes

Singh, S, PhD^1^, Buch, S, PhD^1^, Periyasamy, P, PhD^1^; ^1^Department of Pharmacology and Experimental Neuroscience, University of Nebraska Medical Center, Omaha, NE, 68198, United States.

Nearly half of people living with HIV (PLWH) are alcohol abusers, placing them at a 2–3-fold higher risk of developing HIV-associated neurological disorders (HAND) which significantly impact public health. Our group has recently identified that the astrocyte-specific inflammasome NLRP6 plays a critical role in HIV Tat and ethanol-induced neuroinflammation. Based on the premise that alcohol use exacerbates HIV-associated neuroinflammation, we explored the epigenetic regulation of NLRP6-mediated neuroinflammation in HIV Tat and ethanol-exposed astrocytes. We demonstrated that exposure of mouse primary astrocytes (mPAs) to HIV Tat (50 ng/mL) and ethanol (50 mM) synergistically activated astrocyte markers and the NLRP6 inflammasome.This combined exposure also increased NLRP6 downstream signaling mediators-caspase-1, and proinflammatory cytokines- IL-1β and IL-18. Notably, silencing NLRP6 expression using siRNA inhibited these effects, confirming its pivotal role in HIV Tat and ethanol-mediated neuroinflammation. Additionally, our miRNA microarray study revealed significant downregulation of miR-339, which was confirmed to target the 3’-UTR of NLRP6 mRNA using TargetScan and argonaute immunoprecipitation assays. Overexpression of miR-339 in mPAs exposed to HIV Tat and ethanol validated its role in the epigenetic regulation of NLRP6 signaling. In summary, our findings provide novel insights into the miR-339-mediated epigenetic regulation of NLRP6 activation in HIV Tat and ethanol-exposed astrocytes, advancing our understanding of HAND pathogenesis in the context of alcohol use. Supported by Startup funding from the UNMC to PP, NIAAA (AA031444; P50AA030407-5126, Pilot Core grant) to SS. and NCSAR

### 40 Differential reactivity of astrocytes and neurons in brain organoids exposed to bacterial products from oral pathogens

Soto-Nieves, A^1^, Delgado-Berrios, V^2^, Torres, J, BS^3^, Lasalde, C, PhD^3^, Pérez-Santiago, J, PhD^3^, Cantres-Rosario, YM, PhD^3^; ^1^Cellular-Molecular Biology, University of Puerto Rico-Rio Piedras Campus, San Juan, PR, 00925 United States. ^2^Biology, University of Puerto Rico-Bayamon Campus, Bayamon, PR, 00959 United States. ^3^Medical Zoology and Microbiology, University of Puerto Rico-Medical Sciences Campus, San Juan, PR, 00936 United States.

Neurodegenerative diseases (NDs) are linked to inflammation and neuronal dysfunction. Growing evidence on the oral microbiome-brain interplay has shown microbial products from oral pathogens, such as Porphyromonas gingivalis lipopolysaccharide (Pg-LPS), in post-mortem brain tissue of ND patients. We hypothesize that these molecules potentiate neuronal dysfunction and inflammation, supporting the oral-microbiome-brain axis and periodontal disease in ND development. However, the molecular mechanisms remain unknown. In this study, human primary astrocytes were exposed to Pg-LPS for 24–48 hours at several concentrations. Astrocyte viability significantly increased (p=0.023), but no changes were observed in reactivity (GFAP levels). Moreover, inflammatory cytokines TNF-alpha and IL-6 slightly decreased upon exposure. To assess neuronal signaling, human iPSC-derived cortical brain organoids were treated with Pg-LPS, E. coli LPS, and lipoteichoic acid (LTA) for 24 hours. TNF-α levels increased nearly 5-fold in Pg-LPS-treated organoids compared to controls and other treatments. In contrast, IL-6 was undetectable in Pg-LPS-treated organoids and decreased in LTA-treated organoids. Since brain organoids are composed of astrocytes, neurons and oligodendrocytes, these preliminary findings suggest differential reactivity of brain-resident cells to bacterial products exposure. Further studies are being conducted to characterize neuronal function and identify inflammatory mechanisms. Supported by Título V RCM PO31S200104NIHMHD U54 MD007600, NINDS K22NS118975, 5R21NS131061-02, NIGMS PR-INBRE 5P20GM103475, and HiREC S21MD0.

### 41 Fucosylation improves Treg neuroprotective in a model of Parkinson’s disease

Srivastava, SS, BS^1^, Bhattarai, SB, MS^1^, Foster, EF, BS^1^, Kadry, RK, MS^1^, Lu, YL, MS^1^, Mosley, RLM, PhD^1^, Yeapuri, PY, PhD^1^, Ramakrishnan, GP, PhD^2^, Hu, GH, PhD^1^, Gendelman, HEG, MD^1^; ^1^Department of Pharmacology and Experimental Neuroscience, University of Nebraska Medical Center, Omaha, NE, 68198 United States. ^2^Targazyme. Inc, N/A, Carlsbad, CA, 92011 United States.

Our laboratories have pioneered a therapeutic role for regulatory T cells (Treg) for Parkinson’s disease (PD), amongst other neurodegenerative disorders. While each disease has independent pathologies, all are linked to genetic, environmental, and inflammatory pathways that accelerate neuronal damage. Over the past two decades, our laboratories have discovered the pharmacologic control of innate and adaptive immunity for the control of neurodegeneration. The therapeutic successes were linked to the control of intracellular α-synuclein aggregation and Lewy Body formation. Improving Treg function with interleukin-2 and granulocyte-macrophage colony-stimulating factor was associated with improved immune homeostasis and neuroprotection. However, long-term successful clinical outcomes were hindered by poor homing and reduced Treg survival. We proposed that fucosylation of Tregs and generating Fuco-Tregs can enhance cell trafficking to inflamed nigrostriatal sites, improve neuronal survival, and mitigate associated PD pathologies. To test this idea we isolated then fucosylated Tregs using Targazyme’s™ TZ101 fucosylating agent, and fucosylation was confirmed by CLA staining via flow cytometry. Fucosylation increased CLA expression on Tregs by 30%. Mice were MPTP intoxicated and 1 million Tregs or Fuco-Tregs were adoptively transferred. Dopaminergic neuronal survival was assessed following MPTP toxication by stereotactic analysis of tyrosine hydroxylase (Th1)-stained midbrain. Fuco-Tregs improved dopaminergic neuronal survival by 60% after MPTP intoxication.

### 42 Modulation of neuroinflammation by methamphetamine – implications for HIV-associated brain injury

Tayabally, S, MS^1^, Koury, J, PhD^1^, Maung, R, MS^1^, Yuan, N, PhD^1^, Kaul, M, PhD^1^; ^1^School of Medicine, Division of Biomedical Sciences, University of California, Riverside, Riverside, CA, 92521 United States.

Methamphetamine (METH) is a potent addictive substance with high abuse rates, particularly among people living with HIV (PLWH) on combination antiretroviral therapy (cART). The interaction between HIV and METH exacerbates HIV-associated neurocognitive impairment (NCI) and neuronal damage, potentially through inflammatory processes. Peripheral HIV-infected monocytes/macrophages infiltrate the brain, releasing neurotoxins and pro-inflammatory factors, while the virus also infects microglia. This study investigated the in vitro effects of METH on monocytic THP-1 cells, the human microglial cell line HMC3, and induced pluripotent stem cell-derived microglia. Stimulation with the HIV LTR-mimic ssRNA40 or HIV-1 infection revealed that METH elevated inflammatory enzymes such as MPO and MMP-9 while increasing infection levels. Concurrently, METH reduced the antiviral cytokine IFNβ, downregulated IFN-γ, and decreased CXCL10/IP-10 protein expression, impairing immune activation and antiviral responses. These findings suggest that METH diminishes neuroprotective and antiviral immune responses while promoting pro-inflammatory pathways, potentially accelerating HIV-associated neuropathology. Supported by NIH/R01 MH087332, R01 DA052209, P50 DA026306

### 43 The role of dopamine D1 receptors in methamphetamine-induced microglial activation and oxidative stress

Torres, J, BS^1^, Toews, G^1^, Gunubu, A^1^, Williams, C^1^, Chivero, E.T., PhD^1^; ^1^Department of Psychology, Neuroscience Program, University of Nebraska at Omaha, Omaha, NE, 68182 United States.

Methamphetamine (meth) is a potent psychostimulant with negative acute and chronic effects, that stem from the dysregulation of dopamine-receptor mediated homeostasis. Although D1-type dopamine receptors (D1DRs) have been implicated in meth-induced oxidative stress and microglial activation, the underlying mechanisms have yet to be clearly elucidated. Here we investigated the role of D1DR antagonism on cellular activation and oxidative stress in microglia exposed to meth by establishing a dose-curve and determining a time course. Then microglia were pre-treated with SCH23390, a D1DR inhibitor, to measure the levels of HO-1 and Iba-1 as markers of oxidative stress and cellular activation. Furthermore, siRNA knockdown of D1DR was used to confirm their role in meth-induced effects. We found dose- and time-dependent increases in the expression of these markers as well as decreases following D1DR antagonism and knockdown. The effects of meth were validated in the striatal brain region of mice administered with a binge-dose of meth for 14 days. Overall, these findings confirm the role of D1DR signaling in mediating oxidative stress in microglia in the context of meth. Future directions will determine the generalization of these findings to other cell types and brain regions in the context of psychostimulant use. Supported by UNO start-up fund to EC

### 44 Sex-dependent effects of chronic HIV-1 Nef exposure and acute cocaine administration on dendritic spines

Velazquez, B, BS^1^, Ufarry, A, MS^1^, Noel, R, PhD^1^; ^1^Department of Basic Sciences, Ponce Research Institute, Ponce Health Sciences University, Ponce, PR, 00716 United States.

Cocaine use is prevalent among individuals with Human Immunodeficiency Virus (HIV) and contributes to the development or progression of HIV- associated neurocognitive disorders (HAND). The accessory protein Nef plays a critical role in the viral toxicity and contributes to changes in plasticity hallmarks such as dendritic spines. Dendritic spines are dynamic structures that can alter functioning depending on the physiological stimuli. The viral protein Nef and cocaine alter synaptic plasticity in different regions such as hippocampus and nucleus accumbens (NAc). The combined effects of Nef and acute cocaine exposure could cause neuronal damage and disrupt synaptic plasticity. Here, we hypothesize that the combined effect of Nef and acute exposure to cocaine changes spine density and synaptic proteins in the hippocampus and NAc. We used Sprague Dawley male and female rats that received a lentiviral infusion of either Lenti-GFAP-SF2Nef-IRES-mCherry or Lenti-GFAP-IRES-mCherry and a single dose of cocaine or saline. Brain tissues were collected for Golgi staining to assess spine density and lysates for synaptic protein expression. Preliminary data showed sex differences in PSD95, Arc and GluR2 protein expression dependent of the brain region. In contrast, preliminary spine density data did not show any differences between treatment groups or sexes. We found that Nef appears to directly affect protein expression in female and male rats in the NAc but not in the Hip. These findings suggest distinct changes in synaptic integrity depending on the treatment and brain region.

Supported by U54MD007579

### 45 Insights into the increased risk for chronic kidney disease in people living with HIV: Role of the glycolysis and inflammation-induced byproduct, methylglyoxal

Venn, ZL^1^, Zachary, K, BS^1^, Tu, R^1^, Zhang, C, PhD^1^, Bidasee, SR^1^, Aaris, T, BS^1^, Fox, H, PhD^2^, Dash, PK, PhD^1^, Alomar, FA, PhD^1^, Edagwa, B, PhD^1^, Gorantla, S, PhD^1^, Bidasee, KR, PhD^1^; ^1^Pharmacology and Experimental Neuroscience, University of Nebraska Medical Center, Omaha, NE, 68198 United States. ^2^Neurological Sciences, University of Nebraska Medical Center, Omaha, NE, 68198 United States.

CKD is a major cause of morbidity in people with HIV-1 infection (PLWH). The causes for this remain poorly understood. Glycolysis is elevated in PLWH. In addition to metabolic substrates, glycolysis also generates a toxic byproduct methylglyoxal (MG). The associated increase in oxidative stress and inflammation also downregulates the MG-degrading enzyme glyoxalase1 (Glo1). Whether MG is contributing to renal disease in HIV-1 infection is not known. Hu-mice were infected with HIV-1. After 4 weeks, infected mice were divided into 2 groups and one group was treated with DTG/TDF/FTC for 12 weeks. Before euthanasia, BSA-FITC was intravenously injected into animals to assess perfused microvessels/ischemia. Kidneys were harvested and assessed for fibrosis, MG, Glo1 and the MG-generating enzyme vascular adhesion protein 1 (VAP-1). Autopsied human and monkey tissues were also stained for MG, Glo1 and VAP-1. After 16 weeks of infection, BSA-FITC was 3-fold higher in glomeruli over controls. Perfused microvessels around nephrons was reduced by 40% with microvascular leakage in the medullar. MG and VAP-1 were elevated 2-fold in tubules, Glo1 was reduced by 61% and fibrosis was increased 3-fold. DTG/TDF/FTC treatment minimally attenuated changes in glomerular hyper-perfusion, fibrosis, MG and VAP-1 and Glo1. Similar changes in MG, VAP-1 and fibrosis and Glo1 were also seen in autopsied kidneys from HIV/SIV-infected humans and monkeys Increases in MG arising from increased synthesis and reduced degradation is contribution to the renal damage seen in HIV-1 infection. Supported by NIH

### 46 Amyloid beta specific regulatory T cell therapy for Alzheimer’s disease

Yeapuri, PY, PhD^1^, Machhi, JM, PhD^1^, Lu, YL, BS^1^, Kadry, RK, MS^1^, Patel, MK, PhD^1^, Bhattarai, SB, MS^1^, Foster, EF, BS^1^, Mosley, RLM, PhD^1^, Gendelman, HE, MD^1^; ^1^Department of Pharmacology and Experimental Neuroscience, University of Nebraska Medical Center, Omaha, NE, 68198 United States.

Regulatory T cells (Tregs) maintain immune tolerance and exhibit neuroprotective effects. However, a lack of antigen specificity limits their therapeutic use in neurodegenerative diseases. This is particularly relevant for Alzheimer’s disease (AD), where precise immune modulation in the affected brain regions is critical. To address this, we engineered Tregs to express a transgenic T cell receptor (TCRAβ) derived from amyloid-beta (Aβ)-specific effector T cell clones, enabling precise antigen recognition and therapeutic targeting. TCRAβ-Tregs were generated via CRISPR-Cas9-mediated knockout of endogenous TCRs, followed by transgenic incorporation of Aβ-specific TCRAβ. Antigen specificity was confirmed using MHC-Aβ tetramers. These engineered Tregs were adoptively transferred into transgenic AD mice expressing mutant amyloid precursor protein (APP) and presenilin-1 (PS1). Behavioral, immune, and histopathological analyses assessed therapeutic efficacy. TCRAβ-Tregs specifically recognized Aβ and migrated to the brain, as evidenced by ^18^F-fluorodeoxyglucose tracking. Their adoptive transfer led to sustained immune regulation, reduced microgliosis, and decreased amyloid burden. Notably, these changes correlated with significant cognitive improvements. TCRAβ-Tregs promote immune homeostasis, reduce amyloid pathology, and enhance cognitive function, underscoring the potential of antigen-specific Treg immunotherapy for AD. This study establishes a foundation for. developing a clinically translatable CAR (chimeric antigen receptor) Treg therapies for neurodegenerative diseases. Supported by NIH, Nebraska Foundation,

### 47 HIV-1 gp120, a potential pruritogens for HIV associated itch in aging mice

Yuan, SY, MD, PhD^1^, Liew , JL, BS^1^, Sbei, SS, PhD^1^, Zheng, YZ, BS^1^, Green , DG, PhD^1^; ^1^Dept of Neurobiology, University of Texas Medical Branch , Galveston, TX, 77555 United States.

HIV associated chronic itch (HIV-ITCH) is a common comorbidity affecting 30–45% of the 39 million people living with HIV(PLWH). HIV-ITCH is resistant to routine anti-histamines and no FDA approved drugs due to unknown pruritogens and poorly understood pathogenesis. This study is to clarify if HIV viral proteins are pruritogens. gp120Bal, a coat protein of commonly transmitted HIV-1 strain, binds to CCR5 coreceptor on CD4 cells. With dosages of 150ng/20g body weight (gbw) on adult female mice, we unexpectedly found the itch scratch bouts of gp120 group did not significantly increase compared with vehicle group. Dose response test also showed similar scratch bouts as the vehicle group. Another gp120 subtype, IIIB and Tat, both did not increase scratch bouts on adult female mice. Considering 51% PLWH are aging patients, gp120Bal was tested on aging male mice. Interestingly, we found scratch bouts in gp120Bal group significantly increased. Most of the bouts occurred within 20min after gp120Bal injection and was persistent for three individual tests. Aging mice (10 months) with Mas-related G protein-couple receptor (Mrgpr) cluster gene knockout (KO) received gp120Bal 150ng, the scratch bouts significantly decreased in KO mice compared with wild type (WT) mice, indicating that Mrgpr pruriceptors mediated gp120 induced itch. Altogether, gp120Bal induced itch in aging mice, revealed its potential as a pruritogen. Next, we will evaluate weather gp120IIIB, Tat and Vpr are pruritogens on aging mice and will investigate the underlaying mechanisms. Supported by NINDS to S Yuan R01NS122571. Claude D. Pepper Older Americans Independence Center P30 AG024832 as financial support to S Yuan

### 48 Unique Molecular Signatures in Rebound Viruses from Antiretroviral Drug and CRISPR Treated HIV-1-Infected Humanized Mice

Zhang, C, PhD^1^, Li, HT, MS^2^, Poluektova, LY, MD, PhD^1^, Gendelman, HE, MD^1^, Dash, PK, PhD^1^; ^1^Department of Pharmacology and Experimental Neuroscience , University of Nebraska Medical Center, Omaha, NE, 68105 United States. ^2^School of Biological Sciences, University of Nebraska-Lincoln, Lincoln, NE, 68588 United States.

HIV-1 elimination from a subset of virus-infected humanized mice (hu-mice) is reported following sequential dual-treated long-acting (LA) antiretroviral (ART) and CRISPR-Cas9 therapies. However, viral rebound is observed in >50% of the dual-treated animals. The molecular signatures of the rebound virus, recovered from plasma, are now determined. The LA-ART treatment contains dolutegravir, lamivudine, abacavir, and rilpivirine combinations and HIV-1 excision treatment is CRISPR-Cas9 targeting the HIV-1-LTR-gag. One-step reverse transcriptase polymerase chain reaction, which avoids spontaneous preparatory mutations is performed on plasma-derived RNA. Sanger and Next-Generation Sequencing is employed targeting the HIV-1-gag, pol, and env genes. HIV-1env shows the most divergence. LA-ART, with or without CRISPR, is responsible for the new mutations. The major and accessory mutations are detected by deep sequencing. Viral evolution reflects changes in virus as reported from ART-treated and HIV-1 infected patients. No major CRISPR-specific mutations are observed. The molecular viral signatures demonstrate an accelerated HIV-1 drug resistance escape from ART rather than from the generation of CRISPR mutants. These define viral rebound in the dual-treated hu-mice. Taken together, the data underscores the limited role of CRISPR excision in the generation of these rebound HIV-1 mutants from dual-treated hu-mice.

### 49 The Hidden side of a β chemokine: CCL2 controls HIV-1 replication fitness by modulating ESCRT factors

Dina Mofed, Anjali Gowripalan, Anthony Morante, Kimberly Merani and Vinayaka R. Prasad

Department of Microbiology and Immunology, Albert Einstein College of Medicine, Bronx, NY 10461

Infiltration of mononuclear phagocytes (MPs) is a major pathway for HIV-1 to establish CNS infection. While it is well known that CCL2 plays a definitive role in recruiting MPs to CNS, its remarkable impact on virus replication fitness is poorly appreciated. We previously reported that blocking CCL2-signaling in macrophages strongly inhibits (10-fold) HIV-1 replication. The inhibition was at the stage of HIV virion budding and release. We showed that CCL2 mobilizes ALIX from F-actin to cytosol, while CCL2 signaling blockade sequesters ALIX to F-actin. Soluble ALIX is recruited to virus assembly sites on plasma membrane to facilitate virus bud neck cleavage and stimulation of HIV production. CCL2-mediated increase in HIV-1 production depended on LYPX late motif on Gag that binds ALIX (Ajasin et al. (2019) eLIFE). To understand the basis for a striking influence of CCL2 on HIV-1 replication, we examined the role of ALIX downstream players. The role of ALIX in HIV budding is to recruit another ESCRT factor, CHMP4b, which facilitates membrane fission at virus bud neck. We tested the consequences of CCL2 signaling blockade, which sequesters ALIX to F-actin, on mCherry-CHMP4b using CCL2/CCR2 CRISPR-Cas9 knockout HeLa cells. CHMP4b was equally distributed throughout the cell in HeLa cells, but predominantly localized to nucleus in CCL2/CCR2 KO cells. We hypothesized that nuclear CHMP4b localization occurs when ALIX is unavailable for CHMP4b binding due to ALIX sequestration on F-actin. We tested this notion *via* the use of: (i) ALIX knock-down HeLa cells; (ii) ALIX-binding defective CHMP4b mutants in HeLa cells and (iii) CHMP4b-binding-defective ALIX mutants in HeLa cells. In all of these circumstances, we observed that CHMP4b localizes to nucleus confirming that ALIX-binding is required to maintain CHMP4b in cytosol and thus for efficient virus budding. We next wished to investigate the pivotal role of F-actin-binding of ALIX in the regulation of HIV-1 budding by CCL2. We hypothesized that an F-actin-binding defective ALIX should allow HIV to overcome regulation by CCL2. Informed by the structure of ALIX-CHMP4b interaction interface, we created a panel of 14 mutations in seven selected residues in the proposed F-actin-binding domain of ALIX Bro I domain. We developed an assay in which, an ALIX-mGreenLantern fusion protein introduced into HeLa cells localizes to F-actin only in the presence of anti-CCL2, but not in the presence of CCL2. Several of the mutants tested were unable to associate with F-actin in the presence of anti-CCL2. Such mutant ALIX proteins, when introduced into HeLa cells silenced for ALIX expression, were constitutively localized to the cytosol and produced higher virus levels. Our results provide evidence that ALIX interaction with F-actin is a central aspect of CCL2-mediated regulation of HIV-1 budding and viral replication fitness. We propose that targeting this aspect of CCL2 action can serve as a countermeasure to reduce CNS viral reservoirs.

